# Risk assessment for mycotoxin contamination in fish feeds in Europe

**DOI:** 10.1007/s12550-019-00368-6

**Published:** 2019-07-26

**Authors:** Constanze Pietsch

**Affiliations:** Institute of Natural Resource Sciences (IUNR), Zurich University of Applied Sciences (ZHAW), Grüental, P.O. Box, 8820 Wädenswil, Switzerland

**Keywords:** Mycotoxin, Aquaculture, Mycotoxin effects, Safety factors, Bayesian modelling, Species sensitivity

## Abstract

Mycotoxins are difficult to monitor continuously, and a tool to assess the risk would help to judge if there is a particular risk due to the inclusion of certain feed ingredients. For this, the toxin contents of 97 commercial fish feeds have been estimated, and the most prominent toxins in fish feed are calculated to be deoxynivalenol, zearalenone, fumonisins and enniatins. These pose a risk to fish well-being, as can be calculated by the Bayesian models for determining the critical concentrations 5% (CC5) for the different toxins. Besides fishmeal, wheat, soybean products and corn are regularly used as fish feed ingredients. The calculated scenarios show that fish are at high risk of toxin contamination if feed ingredients of low quality are chosen for feed production. Due to this, specific maximum allowable levels for several mycotoxins in fish feeds should be established.

**Electronic supplementary material:**

The online version of this article (10.1007/s12550-019-00368-6) contains supplementary material, which is available to authorized users.

## Introduction

### Importance of mycotoxins and their occurrence in cereals and other feed ingredients

In the present study, contamination levels with several different mycotoxins have been considered since fish production in aquaculture is continuously increasing. To feed fish in aquaculture, increasing amounts of fish feeds are needed. Fishmeal, as an important ingredient of fish feeds, will not be available in sufficient amounts in the future. As a result, cereals are often used to replace at least a part of the fishmeal in fish feeds. However, the ingredients are often contaminated with several mycotoxins. The 10 most commonly and most problematic mycotoxins in cereals and other feed ingredients, that have been identified so far, have been included in this estimation. These were: aflatoxin B_1_ (AFB_1_), deoxynivalenol (DON) and nivalenol (NIV), zearalenone (ZEN), ochratoxin A (OTA), T-2 toxin (T-2), fumonisin B_1_ (FB_1_), moniliformin (MON), enniatins (ENN) and beauvericin (BEA). Since these toxins are produced by different fungi, their occurrence in feed ingredients also varies.

Aflatoxins are derivatives of difuranocumarin and, due to their natural fluorescence in blue or green, are classified as B_1_, B_2_, G_1_ and G_2_. Aflatoxins are typically produced by certain *Aspergillus* species (Vaamonde et al. 2003). In case of corn as a feed ingredient, aflatoxins are problematic because maize plants on the field can be infected by *Aspergillus flavus* and related species. It is thought that climate changes will lead to a broader distribution of *Aspergillus* species in Europe, e.g. in Hungary and Serbia, resulting in higher contamination in cereals (Tóth et al. 2012; Dobolyi et al. 2013; Lević et al. 2013). For AFB_1_ formation by *Aspergillusflavus* on soybean, the plant-derived compounds phytoalexin–glyceollin have been described to affect toxin production (Song and Karr 1993). Therefore, it is important to monitor aflatoxins especially in commodities that have a higher risk of toxin formation. Since aflatoxins are less resistant to thermal processes and extrusion has been shown to reduce the final aflatoxin concentrations during fish feed processing (Manning et al. 2005), their occurrence in feed is often low. However, after improper storage, high toxin concentrations can be observed. Aflatoxin M_1_ does not play an important role in fish which is the reason why the present study will only focus on AFB_1_.

OTA is the most prominent and most toxic member of the group of ochratoxins and is an isocumarin derivative that is produced by different *Penicillium* and *Aspergillus* species (Van der Merwe et al. 1965a, b). OTA has been detected in cereal-based feed ingredients and feeds (Binder et al. 2007; Duarte et al. 2010; Rodrigues and Naehrer 2012), and similar to the aflatoxins, its occurrence seems to be connected to humidity and temperature during crop growth and storage of feed ingredients and processed feeds. Contaminated feed products lead to the introduction of OTA in the food chain, and a risk for humans and farm animals is assumed (Duarte et al. 2011), probably due to the fact that OTA is more stable in the environment than, for example, aflatoxins (Moss 2002; Duarte et al. 2010). For cereals and cereal products intended for animal feeding, the guidance level for OTA was set to 250 μg/kg (European Commission 2006), while guidance levels between 20 and 2000 μg/kg OTA have been reported for non-EU countries (Van Egmond and Jonker 2004).

Fumonisins are a group of diesters that are produced by *Fusarium* fungi (mainly by *F. verticilloides* (synonym: *F. moniliforme*) and *F. proliferatum*). For most commodities, the occurrence of FB_1_ has been found in significant amounts and is especially a problem in corn (Nelson et al. 1993). FB_1_ has also been recognised as a problem in aquaculture because it represents 70% or more of the total fumonisin content in naturally contaminated feed (Griessler and Encarnacao 2009). However, until now, fumonisins have not been not considered to be highly problematic contaminants of animal feeds due to their rather low stability in feed production processes. Nevertheless, processes that lead to a hydrolysation of the tricarboxylic acid chain in FB_1_ have been reported to result in higher toxicity (Scott 2012). Disorders in vertebrates due to FB_1_ exposure have been found, whereby the most important ailment includes disruption of sphingolipid metabolism (Wang et al. 1992). The recommended guidance values for FB_1_ and FB_2_ in complementary and complete feeding stuffs have been set to a guidance level of 10 mg/kg by the European Commission (2006). Similar to that, the guidance level in feeding stuffs has been set to 10 mg/kg in the USA, but only a few further countries have already defined distinct guidance levels for fumonisins in feeds (Van Egmond and Jonker 2004).

ZEN is a resorcylic acid lactone that occurs after infection with *Fusarium graminearum* or *F. sporotrichoides* in the field, but also during the storage of cereals (Caldwell et al. 1970; Milano and Lopez 1991). ZEN occurrence in some commodities, e.g. in soybeans, is assumed to the less high since soybeans contain substances that limit the production of this toxin by *Fusarium* fungi (Vaamonde and Bonera 1987). The guidance level for ZEN in cereals and cereal products has been set at 2000 μg/kg in the EU, while ZEN concentrations in maize by-products should not exceed 3000 μg/kg. Additional regulations in other countries include maximal ZEN levels of 20 to 1000 μg/kg (Van Egmond and Jonker 2004).

The group of trichothecenes comprises of several mycotoxins produced by different fungi belonging to the genera *Fusarium*, *Cephalosporium* and *Stachybotrys* in different commodities. The most important mycotoxins belonging to this group include T-2 toxin, DON and NIV. Of these, deoxynivalenol shows the highest prevalence and incidence in cereals and feeds in Europe (Rodrigues and Naehrer 2012). The European Commission has established guidance levels of 250 μg/kg for T-2 toxin in compound feeds for farm animals (European Commission 2013). Some countries have also released individual recommendations (of maximum 80 to 100 μg/kg) on the occurrence of T-2 toxin in complete feed and all grains (Van Egmond and Jonker 2004).

*Fusarium* fungi also produce less well-described mycotoxins, so-called emerging mycotoxins, including BEA, ENNs and MON, which have not been included in recommendations by the European legislation. ENNs and BEA are cyclic hexadepsipeptides and can be differentiated according to their alternating N-methylated amino acids (ENN A = isoleucine, ENN B = valine, ENN C = leucin, BEA = phenylalanine), while the core structure is based on an 18-membered ring structure joined by amide and ester bonds (Hilgenfeld and Saenger 1982). Interestingly, ENNs are thought to be rarely produced by trichothecene-producing *Fusarium* strains (Desjardins 2006). Still, ENNs and BEA can show high prevalence in cereals (> 90%, Lindblad et al. 2013), but since they have not been investigated regularly over the last decades, data on their occurrence in many commodities are lacking.

Another *Fusarium*-derived mycotoxin is moniliformin (MON) which is the sodium or potassium salt of 1-hydroxycyclobut-1-ene-3,4-dione. MON is mainly produced by *Fusarium proliferatum* and *F. subglutinans* (Bullerman 2003). MON in animal feeds has been found at concentrations of up to 1.2 mg/kg (Labuda et al. 2005).

### Toxicity in humans and higher vertebrates

This section aims at addressing what is known about the toxicity of the selected mycotoxins on humans and farm animals. For comparison, their effects on fish will be summarised and described in detail in a subsequent section of this study. Generally, mycotoxin contamination of feeds is known to affect the digestibility of the nutrients (Broom 2015) and growth performance of farmed animals, but also leads to a variety of toxic effects. The type of toxicity mainly depends on the chemical structure of the toxin, but also on their concentration, durations of exposure and the life stage that is exposed to the toxins.

Accordingly, the acute toxicology, mutagenicity and carcinogenicity of AFB_1_ are the main risks after exposure of higher vertebrates to this toxin, and these endpoints have been well documented in humans as well as in farm animals (Ramos and Hernández 1997). The IARC has classified AFB_1_ as a human carcinogen (group 1A; Ostry et al. 2017).

Due to its stability during food processing (Duarte et al. 2010), OTA is considered to be problematic for humans and farm animals as well. In higher vertebrates, toxic effects of OTA are mainly observed in the kidney and liver but have also been reported to be teratogenic and immunotoxic (Duarte et al. 2011). Consequently, a higher susceptibility to disease and more secondary infections have been observed. In rodents, carcinogenic effects have also been reported (Boorman 1989). However, Supamattaya et al. (2005) observed that shrimp feeds contaminated with OTA (1 mg/kg) did not negatively affect the shrimp farming industry.

Up to now, the possible carcinogenicity of fumonisins has not been clarified. Consequently, they have been classified as potential carcinogens to humans by the IARC (group 2B; Ostry et al. 2017). The main reason why fumonisins are toxic is their structural similarity to backbone precursors of sphingolipids. Fumonisins are consequently known to interfere with the metabolism of sphingolipids (Voss and Riley 2013). The most predominant fumonisin is FB_1_ which has been described to be nephrotoxic and hepatotoxic in several species (Mathur et al. 2001). After dietary exposure to fumonisins, loss of appetite, reduced litter weight, detrimental effects on fetal development and fetal mortality, respiratory problems, pulmonary edema, hepatic damages and carcinoma, fibrosis, neurotoxicity, hypercholesterolemia, lethargy and immunosuppression have been described in higher vertebrates (Stockmann-Juvala and Savolainen 2008).

According to several studies, ZEN is known to be rapidly absorbed in the intestine, but also undergoes rapid metabolisation and excretion in animals and humans (Zinedine et al. 2007). ZEN and its derivatives are the only known mycotoxins with estrogenic potential and are classified as endocrine-disrupting substances (Bucheli et al. 2005). In addition, ZEN has been found to be genotoxic and is being assumed to be a possible carcinogen (group 2B; Ostry et al. 2017).

Less extensive reports have been accumulated on the toxicity of the emerging mycotoxins ENN and BEA. At a cellular level, they are able to interact with cations and function as ionophores (Ovchinnikov et al. 1974; Hilgenfeld and Saenger 1982). Furthermore, ENNs can lead to lysosomal disruption (Ivanova et al. 2012), cell cycle arrest (Gammelsrud et al. 2012; Devreese et al. 2013), decreased functioning of macrophages (Ficheux et al. 2013) and interference with mitochondrial functions (Tonshin et al. 2010). In addition, ENNs are able to inhibit the calmodulin-dependent signalling in cells (Mereish et al. 1990) and induce apoptosis or necrosis and nuclear fragmentation (Wätjen et al. 2009; Ivanova et al. 2012).

Another emerging mycotoxin is MON, which has been shown to cause several detrimental physiological effects, lowered growth performance and, at high toxin concentrations, even mortality in higher vertebrates (Kriek et al. 1977; Ledoux et al. 1995). Tissues with a high metabolic rate such as cardiac or hepatic tissue are likely a target for the toxic effects of moniliformin by inhibition of the mitochondrial energy metabolism (Thiel 1978). In other studies, the effects of MON on renal integrity and the immune system have been reported (Harvey et al. 1997; Li et al. 2000). In addition, MON mostly shows additive effects in combination with other mycotoxins (Javed et al. 1993) but did not when MON and DON were both simultaneously fed to birds (Harvey et al. 1997; Morris et al. 1999).

ENNs also had insecticidal effects on the blowfly (*Calliphora ertyhrocephala*) after exposure by injection. Both ENN A and BEA injections were also used against mosquito larvae (*Aedes aegypti*) (Grove and Pople 1980). *Fusarium* extracts containing ENN A and A_1_ were also lethal to invertebrates (Strongman et al. 1988). However, in vivo studies on vertebrates either indicated only low levels of toxicity or no toxicity at all (EFSA 2014; Manyes et al. 2014; Rodríguez-Carrasco et al. 2016). Nevertheless, ENNs are highly lipophilic and are therefore able to accumulate in egg yolk (Jestoi et al. 2009) and various tissues of poultry and mice (CODA-CERVA 2011; Rodríguez-Carrasco et al. 2016). In addition, ENNs and BEA have been shown to modulate ATP-binding transporter molecules and may thereby intensify the action of antibiotics and drugs (Dornetshuber et al. 2009).

Since most studies have concentrated on the toxic effects of mycotoxins on higher vertebrates and mostly ignored the effects on lower vertebrates, the present study will focus on the toxicity of the relevant selected mycotoxins in fish and compare the effects in fish to the already known effects in higher vertebrates.

## Methods

### Calculation of the inclusion percentages of feed ingredients

For each of the 97 commercial fish feeds, the percentage of ingredients was calculated based on the known list of ingredients and nutrient composition data from the local feed producer Granavit AG (Kaiseraugst, Switzerland, for details see Table S1 in the Supplement), the gross nutrient composition of each ingredient and the final composition of the gross nutrients in the fish feeds (focusing on crude protein and crude lipid, Table S1 in Annex 1). The approximation of the feed compositions was based on the nutrient composition published by the feed-processing companies (Fig. S1 in Annex 1). Overall, the protein contents were estimated too high by 0.1% of the value published by the manufacturers for all 97 fish feeds, whereas the crude lipid content was underestimated by 0.1% compared to the values of the feed producers. The calculated mean crude protein content for all 97 fish feeds in this study was 47.3 ± 0.9% (mean ± SEM), whereas the crude lipid content accounted for 15.4 ± 0.6% (mean ± SEM). The calculated feed composition with respect to the feed ingredients that are used is shown in Fig. S2 in Annex 1. From this, it can be seen that fishmeal is still a main component of fish feeds (44.6 ± 2.1%, mean ± SEM), although it has to be noted that four fish feeds contain no fishmeal at all. In the 93 fishmeal-containing fish feeds, the percentage of fishmeal averaged 46.2 ± 1.9%. Wheat flour is also a prominent ingredient in feed, showing a mean percentage of 16.6 ± 1.2% (mean ± SEM) in all 97 feeds and being reported for 87 of the 97 fish feeds. Soybeans and soya by-products are used in 67 of the 97 fish feeds and resulted in a mean percentage in all feeds of 10.7 ± 1.2% (mean ± SEM). The other feed ingredients are present at average percentages of less than 10% in the investigated fish feeds.

### Estimation of the mycotoxin contamination levels and their incidence in feed ingredients

Data from 116 scientific publications comprising mycotoxin contamination reports from food and feed ingredients from Northern Europe (Norway, Sweden, Finland, Estonia, Latvia, Lithuania, Poland, Denmark, UK, Ireland), Central Europe (France, Germany, Austria, Poland, Hungary, Romania, Ukraine, Slovakia, Netherlands, Switzerland, Belgium, Czech Republic, Slovenia, Serbia, Croatia) and Southern Europe (Spain, Portugal, Italy, Yugoslavia, Greece, Bulgaria) have been compiled. The difficulty of comparing these studies was based on the different methodologies for mycotoxin detection and quantification that have been used, which may lead to considerable uncertainties with respect to variations in the sensitivity and the accuracy of the different methods. If a study reported ELISA and HPLC or GC MS/MS results for the same samples, it was assumed that the latter methods yielded more accurate levels than the ELISA technique (e.g. Tansakul et al. 2013). These and additional uncertainties have been summarised in detail in a subsequent section in this study.

Mycotoxin occurrence varies worldwide due to differences in climate and the presence of different species and strains of fungi that show variability in their mycotoxin production abilities (Lević et al. 2013; Schatzmayr and Streit 2013). Therefore, the present study concentrated on collecting data from the literature for contamination of cereals and other feed ingredients from Europe and the UK. For the subsequent calculations, the range (minimum and maximum levels) and the mean of the positive samples have been used. For some studies, only the means of all samples have been reported. If these means were lower than the minimum value (because feed ingredient samples without mycotoxins have been used to calculate these means), the minimum value has then been included in the subsequent calculations within the contamination scenarios. Conjugated toxin levels were not added since it was assumed that metabolites are less toxic in most cases and because the exact levels for conjugates have rarely been reported in sufficient detail. If only the number of samples analysed in total was reported for a certain feed ingredient, the percentage of contaminated samples was estimated by using the average contamination level for this toxin, which was calculated from all studies reporting contamination levels for this commodity.

It is known that contamination in the different feed ingredients differs widely. To summarise the contamination levels in wheat, the values for wheat grains, flakes and wheat flour were used. No differentiation between hard wheat and soft wheat or winter and summer cereals was included, since most studies did not indicate the exact cereal strain that had been sampled. To summarise the mycotoxin contaminations in barley, barley samples and malting barley samples were used. For chicken meat contamination with mycotoxins, a low number of data sets were available. Therefore, data from outside Europe have also been included, assuming that the accumulation of mycotoxins is rather similar and independent of chicken origin. DON is excreted rapidly and no relevant residues in chicken have been detected (Awad et al. 2008). Similarly, FB_1_ clearance in chicken is rapid and values in organs remain low (Vudathala et al. 1994). For distillers’ grain with solubles (DDGS) as a feed ingredient, studies from European samples have rarely reported contamination with mycotoxins. Thus, reports from American studies have also been included in the present calculations.

However, the extent to which feed ingredients are processed prior to the feed production process also influences the mycotoxin contamination levels. For example, Mmongoyo et al. (2017) illustrated that generally more cake samples were contaminated with aflatoxin than whole sunflower seeds. This was thought to be caused by the small number of seeds that may have shown aflatoxin contamination within the entire batch. These contaminated grains may have not been sampled when whole grains were analysed, whereas cake produced from the crushed material has a higher probability to contain material from the highly contaminated seeds which finally results in higher detectable aflatoxin levels. However, other processing of the feed ingredients including cleaning, sorting, milling and thermal processes may also influence the mycotoxin content (Kushiro 2008; Cheli et al. 2013; Kaushik 2015). Accordingly, it can be assumed that milling increases mycotoxin concentration in cereal fractions (e.g. bran) that are commonly used for the production of animal feeds. Nevertheless, depending on the mycotoxins, the contamination level and technological processes, the extent of the modification is different. For example, cleaning prior to milling is known to reduce the mycotoxin levels in wheat since moulded grains, broken kernels and dust can be removed. The extent of mycotoxin reduction in wheat is different for each mycotoxin (cleaning of the grains reduced the levels by 7–63% for DON, 7 to nearly 100% for NIV, 7–40% for ZEN and 62% for T-2 toxin) (Lancova et al. 2008; Neuhof et al. 2008; Cheli et al. 2010; Edwards et al. 2011; Pascale et al. 2011). Since mycotoxin contamination often concerns the outer layers of kernels, debranning can reduce DON contamination by 15 to 78% (Aureli and D’Egidio 2007; Rios et al. 2009; Cheli et al. 2010; Sovrani et al. 2012). This leads to the conclusion that wheat bran is more contaminated with DON, ZEN and ENN B and B_1_ than wheat flour (Vaclavikova et al. 2013; Schwake-Anduschus et al. 2015; Tibola et al. 2015). Wheat bran was used at average levels of 6.8 ± 0.7% (mean ± SEM) in all 97 investigated fish feeds (Fig. 2) and was present in 66 of the 97 fish feeds. However, contamination levels of wheat bran have rarely been reported, which is why wheat flour contamination has been multiplied by a factor of 1.5 to yield a more realistic contamination level for wheat bran. In fact, it has been estimated by Pinotti et al. (2016) that the contamination level of wheat bran is 1.5- to 8-fold higher than wheat flour. Since different methods of milling are used for corn (e.g. dry milling and wet milling), several fractions can be obtained (Alexander 1987) with differing mycotoxin contamination levels (i.e. higher levels in the germ, the bran fractions and the flour intended for animal feed production) (Castells et al. 2008; Schollenberger et al. 2008; Scudamore 2008; Pietri et al. 2009). The characterisation and manipulation of kernel characteristics and milling practices therefore can become important strategies to further reduce mycotoxin contamination in the resultant milling fractions. But it is also known that the quality and the processing performance of cereals can be influenced. Accordingly, it has been reported that *Fusarium* infection decreases the wheat milling performance, affects the yield of flour yield and flour ash and impairs flour brightness and finally also baking performance (Siuda et al. 2010). Consequently, the effects of mycotoxins on the feed extrusion process can also be assumed, but no confirming studies are available yet.

### Compiling toxicity data for fish

From the literature, toxicity values for nearly all mycotoxins of interest have been compiled from 158 different publications. The fish species comprised African catfish (*Clarias gariepinus*), Atlantic salmon (*Salmo salar*), beluga (*Huso huso*), catla (*Catla catla*), channel catfish (*Ictalurus punctatus*), coho salmon (*Oncorhynchus kisutch*), common carp (*Cyprinus carpio*), fathead minnow (*Pimephalis promelas*), gibel carp (*Carassius auratus gibelio*), guppy (*Poecilia reticulata*), matrinxã (*Brycon cephalus*), medaka (*Oryzias latipes*), mosquitofish (*Gambusia affinis*), rainbow trout (*Oncorhynchus mykiss*), red drum (*Sciaenops ocellatus*), rohu (*Labeo rohita*), seabass (*Dicentrarchus labrax*), silver catfish (*Rhamdia quelen*), different tilapia species (*Oreochromis niloticus*, *Oreochromis mossambicus* and hybrid tilapia), Tra catfish (*Pangasius hypophthalmus*), vundu (*Heterobranchus longifilis*), walleye (*Sander vitreus*), yellow catfish (*Pelteobagrus fulvidraco*) and zebrafish (*Danio rerio*). The reports that will be mentioned include the effects of single mycotoxins. However, the occurrence of several mycotoxins in the same feed has a high probability, which can lead to combined effects or the pre-exposure to one mycotoxin, e.g. AFB_1_, leading to higher sensitivity to FB_1_ (Carlson et al. 2001; McKean et al. 2006). The effects of mycotoxin combinations will be valued in the discussion but cannot be included in the calculations in this study due to their rather unknown interactions in different commodities.

For the subsequent calculations, data for the following endpoints have been compiled: behaviour, blood (including haematocrit, changes of blood cell populations, but also plasma enzyme activities), body coloration, body composition (including changes of whole body, fillet and/or liver composition, i.e. hepatosomatic index), genotoxicity and cancer, development of early life stages (with heart development, pigmentation development of the skeleton and hatching as separate parameters if feasible), endocrine effects (only for the estrogenic mycotoxin ZEN), growth (including weight and length, weight gain, but also feed conversion), histology, immune responses (including direct measurements of immune responses after toxin exposure, but also resistance to diseases), mortality and oxidative stress.

### Calculation of the potential risk

Assuming mean (or minimum—if the mean data were not available), median and maximum contamination scenarios, the contamination of the final feeds can be calculated. For further calculations, 100% stability of the mycotoxins in the feeds was assumed (no reduction during the feed processing). It can also be assumed that the accuracy with which the contamination levels can be estimated depends on the number of samples that were analysed. Therefore, a weighted mean has also been included as one contamination scenario. For this scenario, the mean contamination level obtained from each publication in the literature was used for the calculation of the weighted mean contamination level by multiplication of the value with the number of investigated samples in the study divided by the number of samples in all studies for each commodity separately.

To estimate fish toxin uptake, the mycotoxin concentration in the feeds were calculated for each scenario considering the prevalence of each mycotoxin in the different commodities according to the reports in the literature. For the exposure assessment based on a deterministic approach, the estimated daily intake (EDI) levels were obtained by combining the mycotoxin occurrence data obtained from the literature with the assumption of a feed conversion factor of 1.2 for an adult fish with a body weight of 1 kg which means an average conversion of 12 feed per day for a fish weighing 1 kg.

For comparison, the maximum recommended and guidance levels for mycotoxins in animal feed have been compiled (Table 1). In addition, reports on contamination levels in commercially available fish feeds have been assembled (Fig. 4).

**Table 1:** Recommended guidance levels for mycotoxin contamination in animal feeds with relevance for fish species (according to the European Commission 2006 and European Commission 2013)

Mycotoxin	Relevant feed or cereal produce	Guidance level [μg/kg]
Aflatoxin B_1_	All feed materials	20
	Complete feedstuffs (not for cattle, sheep, goats, pigs and poultry)	10
	Complementary feedstuffs (not for cattle, sheep, goats, pigs and poultry)	20
Deoxynivalenol	Cereals and cereal products (except maize by-products)	8000
	Maize by-products	12,000
	Complementary and complete feedstuffs	5000
Zearalenone	Cereals and cereal products (except maize by-products)	2000
	Maize by-products	3000
Ochratoxin A	Cereals and cereal products	250
Fumonisin B_1_	Maize and maize products	60,000
	Complementary and complete feedstuffs	10,000
T-2 and HT-2 toxin	Cereals and cereal products, except oat bran	500

The risk of being affected by a certain chemical can be derived based on the lowest observed effect levels (LOEL) and applying a safety factor to it to be sure that the thus defined safe level lies below the concentration that causes obvious harm to an organism, or by using the no observed effect levels (NOEL) and estimating the safety factor that has to be added to estimate at which toxin concentration threshold first effects in organisms can be expected. In the present study, both approaches have been used. For the first approach, the calculations that are based on the reported LOEL have been adjusted by applying a safety factor of 50 to correct for uncertainties in the assumptions, which is a low level with respect to the number of uncertainties in the present study that will be addressed in detail in the discussion (EEC 1991). Furthermore, the calculation of 95:5 values as species sensitivity ratios (SR) uses the range of sensitivity to a toxic chemical for a certain species according to Elmegaard and Jagers op Akkerhuis (2000). In general, the species-specific sensitivity depends on the chemical characteristics of a certain compound. Therefore, the sensitivities have to be illustrated for each mycotoxin separately. The method of Elmegaard and Jagers op Akkerhuis (2000) allows for the definition of a safe toxin concentration range by displaying the general picture for the toxic substance for which several species have been tested. For this, the size of the SR95:5 for each toxin was illustrated in a graph showing the cumulative frequency distribution of the species-specific SR95:5 values, which shows the percentage of toxins having a SR95:5 lower or equal to *x*. Based on this approach, the data distribution for effects on fish was approximated to a normal distribution of the reported LOEL and the lower 5% quantile including its 95% credibility interval was derived from the posterior normal density function. By calculating the ratio of the 95% credibility interval and the lower 5% quantile, it was possible to compare the sensitivity of different species. A lower SR95:5 indicates a narrower range of toxicity to a mycotoxin and thus a higher specificity in the responses of the fish. Since the SR95:5 indicates the distance between the responses of very insensitive species and very sensitive species, it indicates a safety factor that should be applied to protect a sensitive species even though a test uses an insensitive species.

For the second approach, data sets with sufficient entries for no observed effect levels (NOEL) are needed. In the studies on the toxicity of mycotoxins in fish, even the lowest dose often produced an effect or the control diet was assumed to contain no mycotoxins at all. Therefore, NOEL could only be derived for a small subset of the studies. Given this lack of sufficient NOEL data, all further calculations were conducted with three different data sets: the original LOEL data, the original NOEL data and a NOEL data set predicted from the original LOEL (henceforth “predicted NOEL”). To generate the predicted NOEL data set, a linear modelling approach was implemented in the open-source software R (R Development Core Team 2006) by using a regression model that was fitted to the literature data containing original LOEL and NOEL concentrations. The modelling results for each toxin are shown in Annex IV. A theoretical distribution of the species’ sensitivities can be achieved by illustrating the probability density functions of the NOEL for each mycotoxin. Calculating the confidence intervals yields the concentration range that protects 95% of the fish species. The calculation of such a hazardous concentration according to Luttik and Aldenberg (1997) includes the definition of the concentration threshold that affects 5% of the fish from a theoretical population. This requires the extrapolation of the probability of selecting a species from a data set with a NOEL that is smaller than this concentration is equal to 5% (Van Straalen and Denneman 1989). To derive toxin-specific critical effect concentrations (CC5) that allow the potential effects of the exposure to toxins in aquaculture fish to be estimated, these were calculated for the predicted NOEL data sets using the Bayesian modelling approach described in Aldenberg and Jaworska (2000), since these calculations also allow for the calculation of model reliability. To ensure that a normal distribution was admissible, normality of the data was confirmed for each endpoint using quantile–quantile plots. The entire R-Script used for these analyses can be found in Annex VI.

## Results

### Mycotoxin levels according to the different contamination scenarios for fish feeds

**Fig. 1:**
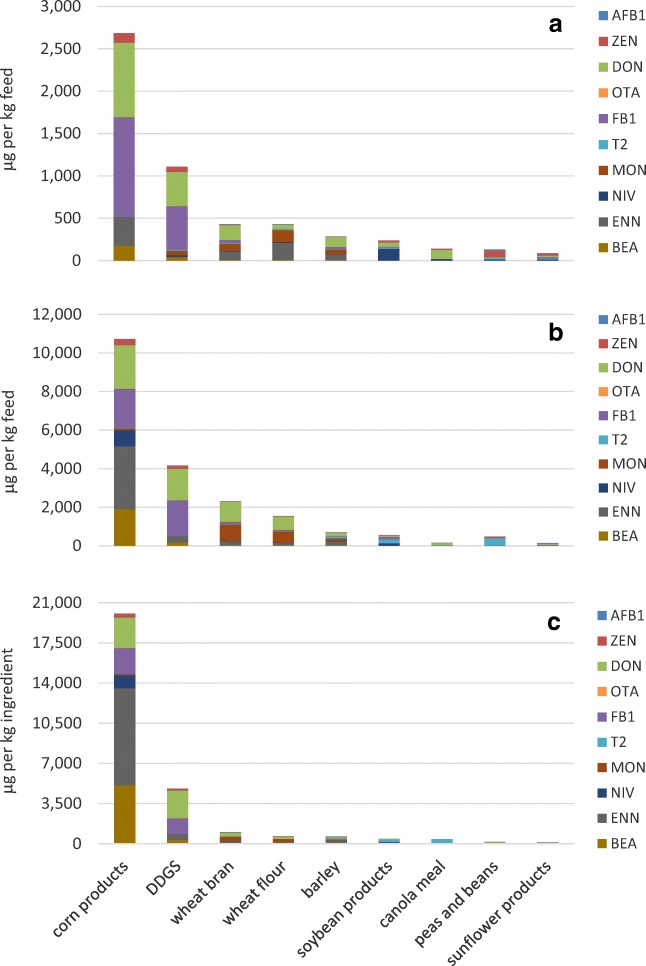
Estimated mycotoxin content in fish feed ingredients calculated from literature data according to the median scenario (**a**), mean scenario (**b**) and mean weighted scenario (**c**) (see Annex II for details) and considering the average prevalence of each mycotoxin in the feed ingredients

According to the assumption of median contamination levels in the feed ingredients, the highest contaminations with mycotoxins occurred in corn products, DDGS and wheat bran (Fig. 1a). The most prominent mycotoxins were FB_1_ (sum 1801 μg including 12 feed ingredients), DON (sum 1791 μg including 12 feed ingredients), ENN (sum 716 μg including 12 feed ingredients) and ZEN (sum 351 μg including 11 feed ingredients). The median contamination of the feed ingredients resulted in the occurrence of toxins in the final fish feeds in which the presence on DON, T-2, MON and NIV is dominating (Table *2*).

**Table 2:** Detailed results for the calculated mycotoxin levels (μg/kg feed) in the 97 fish feeds according to the different contamination scenarios, MW ± SEM

	Median scenario	Mean scenario	Weighted mean scenario	Maximum scenario	Bad-corn scenario	Bad-wheat scenario
AFB_1_	1.2 ± 0.1	1.5 ± 0.1	1.5 ± 0.1	9.1 ± 1.0	5.5 ± 0.9	2.4 ± 0.1
ZEN	8.7 ± 0.7	17.6 ± 1.6	17.1 ± 1.7	496.1 ± 26.4	57.6 ± 10.3	70.5 ± 3.5
DON	57.7 ± 4.8	250.4 ± 15.7	149.2 ± 16.3	1742 ± 150.3	753.9 ± 143.6	1025 ± 49.6
NIV	17.2 ± 1.5	33.7 ± 3.8	38.2 ± 5.0	340.3 ± 67.6	326.1 ± 67.5	52.4 ± 5.2
OTA	0.3 ± 0.0	3.5 ± 0.2	2.0 ± 0.1	14.3 ± 0.6	2.3 ± 0.2	11.0 ± 0.5
FB_1_	33.7 ± 6.0	100.5 ± 12.4	67.1 ± 11.5	796.2 ± 115.9	378.7 ± 77.8	233.6 ± 15.0
MON	18.1 ± 0.9	148.0 ± 7.1	68.8 ± 3.3	1570 ± 327.8	1570 ± 327.8	664.9 ± 32.1
T-2	26.3 ± 2.7	32.0 ± 3.0	31.4 ± 3.0	215.4 ± 39.2	33.8 ± 3.0	43.3 ± 2.9
ENN	22.7 ± 1.9	107.4 ± 14.5	210.4 ± 36.7	68,472 ± 6570	3290 ± 706.7	14,937 ± 720.5
BEA	5.7 ± 0.9	41.5 ± 8.1	105.3 ± 21.9	2692 ± 519.1	2380 ± 517.0	420.2 ± 28.2

According to the mean contamination scenario, the feed ingredients (Fig. 1b) showed slightly higher contamination levels compared with the median contamination level. The most prominent mycotoxins in these contamination scenarios in 12 feed ingredients were DON (sum 4265 μg/kg), FB_1_ (sum 3576 μg/kg), ENN (sum 997 μg/kg) and ZEN (sum 664 μg/kg). As can be seen in Table *2*, the assumption of mean contamination of the feed ingredients resulted in high DON levels in the final feeds, but also to higher levels of MON and ENN.

The weighted mean contamination scenario yielded slightly different contamination levels compared to the mean contamination levels (Fig. 1c). The most prominent mycotoxins in the included 12 feed ingredients were DON (sum 3254 μg/kg), FB_1_ (sum 1429 μg/kg), ENN (sum 1104 μg/kg) and BEA (sum 366 μg/kg). The contamination of the final feeds according to the weighted mean contamination scenario resulted in high levels of ENN, DON, BEA and FB_1_ (Table *2*).

**Fig. 2:**
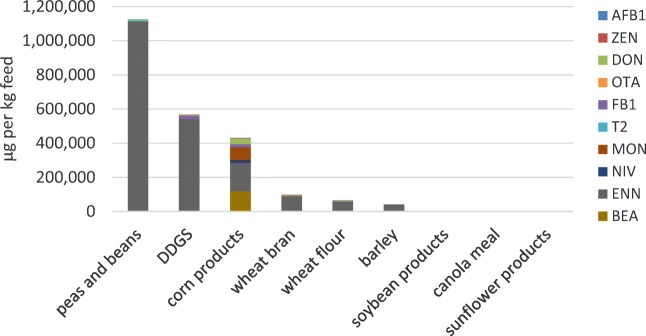
Estimated mycotoxin content in fish feed ingredients calculated from literature data according to the maximum scenario (see Annex II for details) and considering the average prevalence of each mycotoxin in the feed ingredients

The maximum contamination scenario yielded in part very high contamination in feed ingredients, and the ingredients showing the highest toxin levels differed from the previous contamination scenarios (Fig. 2). The highest contaminations with mycotoxins occurred in peas and beans, DDGS, corn products and wheat bran. The most prominent mycotoxins in the included 12 feed ingredients were ENN (sum 884 mg/kg), BEA (sum 128 mg/kg), MON (sum 74 mg/kg) and DON (sum 49 mg/kg). The maximum contamination scenario is mainly characterised by high ENN occurrence in final feeds (Table *2*).

**Fig. 3:**
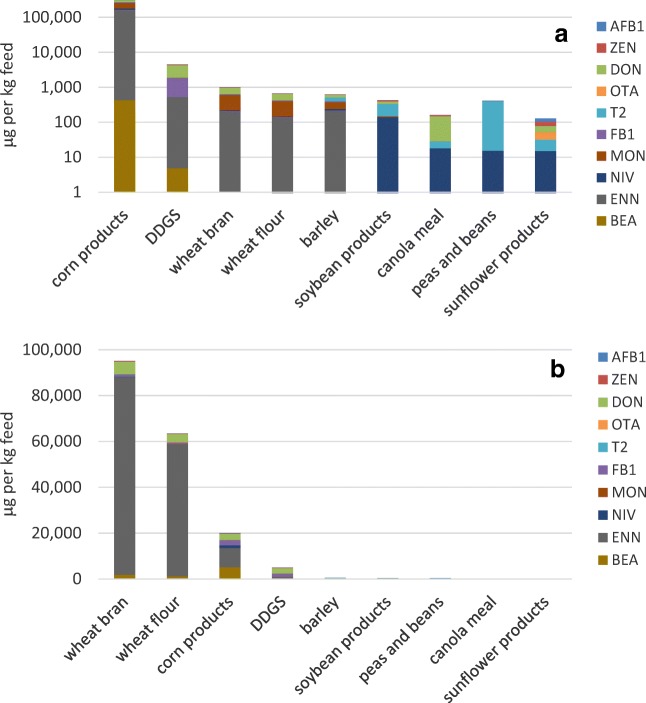
Estimated mycotoxin content in fish feed ingredients calculated from literature data according to the bad-corn scenario (**a**) on a logarithmic scale and the bad-wheat scenario (**b**) (see Annex II for details) and considering the average prevalence of each mycotoxin in the feed ingredients

According to the bad-corn contamination scenario, weighted mean contamination data for all feed ingredients were integrated except for the corn products (Fig. 3a). For the contamination of corn, the maximum mycotoxin contamination was assumed. The contamination of feed ingredients showed the highest levels for ENN and BEA in the compiled 12 feed ingredients (sum levels of 166 mg and 121 mg/kg, respectively), followed by MON, DON and FB_1_ with sum levels of 74, 37 and 19 mg/kg, respectively. In the bad-corn scenario, MON, ENN and BEA are the most prominent mycotoxins occurring in the finished feeds (Table 2).

According to the bad-wheat scenario, weighted mean contamination data for all feed ingredients except for the wheat products were integrated (Fig. 3b). The contamination of feed ingredients showed the highest levels for ENN in the compiled 12 feed ingredients (sum level of 153 mg/kg), followed by DON, BEA, MON and FB_1_ with levels of 14.3, 8.5, 6.7 and 5.3 mg/kg, respectively. In the bad-wheat scenario, the occurrence of ENN dominates the contamination of the final fish feeds. However, DON, MON and BEA also showed higher contamination values than the other mycotoxins (Table 2).

### Comparison with actual contamination levels in fish feeds

**Fig. 4:**
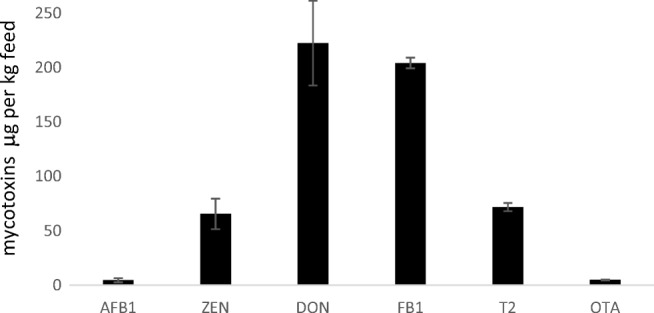
Contamination of aquaculture feeds and naturally contaminated experimental fish feeds with the mycotoxins AFB_1_, ZEN, DON, FB_1_, T-2 and OTA reported in the literature (according to the studies of Boonyaratpalin et al. 2001; Kokic et al. 2009; Deng et al. 2010; Huang et al. 2011; Rajeev Raghavan et al. 2011; Pietsch et al. 2013; Greco et al. 2015)

**Fig. 5:**
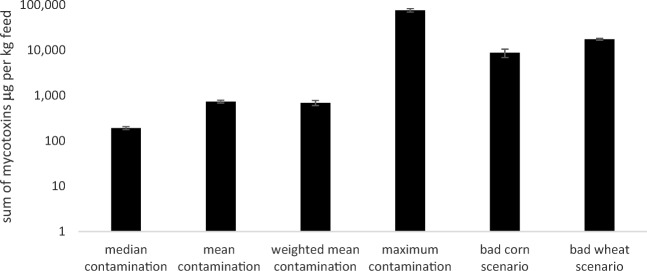
Comparison of the accumulated mycotoxin content in fish feed according to all contamination scenarios. The mean (± SEM) contamination level of 97 fish feeds is shown on a logarithmic scale on the *y*-axis; detailed values are shown in Annex IV

Contamination of commercially available fish feeds has rarely been reported for multiple mycotoxins and published in detail (Fig. 4). Mean contamination with DON and ZEN has been reported, yet for emerging mycotoxins (e.g. MON, FB_1_, ENN, BEA), no sufficient data sets have been published so far. Compared to this, the sum of mycotoxins that has been calculated in the present study indicates a higher incidence and prevalence of mycotoxins in fish feeds (Fig. 5). However, depending on the contamination scenario, these level can differ considerably.

### Toxic effect levels described in fish

Differences in mycotoxin toxicity have already been described in higher vertebrates. For example, poultry species appear to be rather resistant to the effects of fumonisin, DON and ZEN, whereas pigs are very sensitive to DON and T-2 exposure (Kanora and Maes 2009; Murugesan et al. 2015). In the following sections, the species- and endpoint-specific effects of different mycotoxins in fish will be summarised.

#### Effects of AFB_1_ on fish

**Fig. 6:**
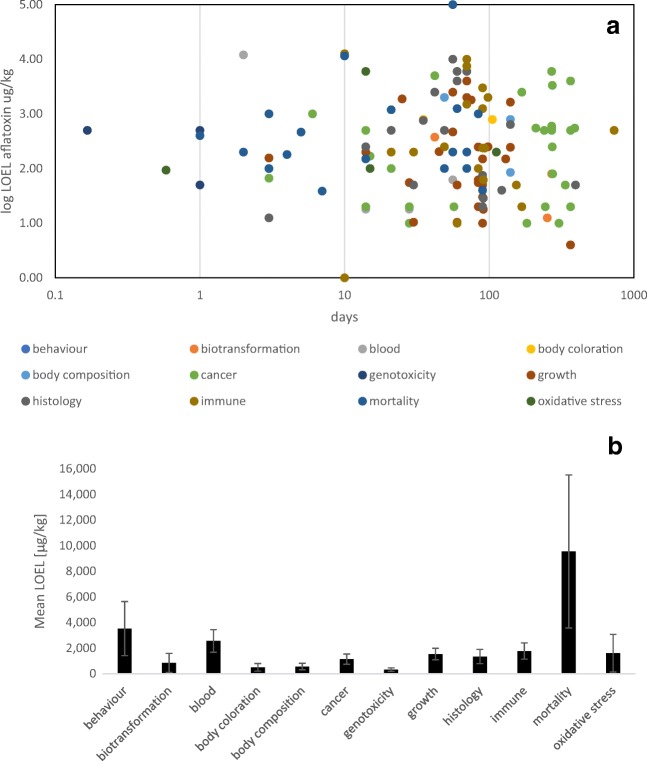
Overview of the lowest observable effect levels (displayed as log 10 LOEL) for different endpoints in different fish species exposed to AFB_1_ for different exposure durations (**a**). The *x*-axis has been logarithmised for better visualisation. The studies that have been compiled for this graph are listed in Annex IV and V. The mean lowest observable effect levels (LOEL) for different endpoints in different fish species exposed to AFB_1_ (**b**) for different exposure durations (mean ± SEM). The number of data points from studies reported in the literature for each endpoint is as follows: behaviour *n* = 5, biotransformation *n* = 8, blood *n* = 19, body coloration *n* = 2, body composition *n* = 10, cancer *n* = 32, genotoxicity *n* = 3, growth *n* = 40, histology *n* = 22, immune responses *n* = 26, mortality *n* = 23, oxidative stress *n* = 4. The studies that have been compiled for this graph are listed in detail in Annex IV and V

Although aflatoxin toxicity has been investigated more thoroughly than for other mycotoxins, uncertainties in the accurate diagnosis of aflatoxicosis in fish still exist. Early aflatoxicosis in fish has often been characterised by hepatic damage, poor growth, pale gills and immunosuppression (Jantrarotai et al. 1990; Sahoo and Mukherjee 2001; Tuan et al. 2002; Akter et al. 2010). However, Fig. 6a reveals that the earliest signs of AFB_1_ toxicity include changes of the body composition and oxidative stress (with mean LOELs of 563 ± 252 and 1598 ± 1467 μg/kg AFB_1_, respectively; mean ± SEM). Nevertheless, growth performance is still a prominent endpoint in studies investigating AFB_1_ effects on fish showing a mean LOEL of 1530 ± 461 μg/kg AFB_1_ (mean ± SEM; Fig. 6b). But the LOEL levels that have been reported for individual fish species are variable. For example, carp exposed to 2 μg/kg AFB_1_ did not show impaired weight gain or body condition (Svobodova and Piskac 1980), and even doses of 20 to 200 μg/kg feed did not impair feeding and protein efficiency (Svobodova et al. 1982). In contrast, exposure of carp fingerlings to 100 μg/kg AFB_1_ significantly reduced their growth performance (Akter et al. 2010). Similarly, the growth of other fish species was impaired by exposure to AFB_1_ concentrations ranging from 1.88 to 10 mg/kg (Jantrarotai and Lovell 1990; Chavez-Sanches et al. 1994; Tuan et al. 2002). In addition, blood parameters can be influenced by elevated AFB_1_ levels (e.g. at levels > 250 μg/kg AFB_1_ in Nile tilapia and at 80 μg/kg AFB_1_ in juvenile sturgeon hybrids) (Tuan et al. 2002; Rajeev Raghavan et al. 2011). Immunosuppressive effects after AFB_1_ treatment have been reported for several fish species with a mean LOEL of 1770 ± 630 μg/kg AFB_1_ (mean ± SEM; Fig. 6b). However, the lowest mean LOELs were noted for the endpoint genotoxicity with 317 ± 136 μg/kg AFB_1_ (mean ± SEM; Fig. 6b). Nevertheless, research on AFB_1_ effects has concentrated on liver carcinogenicity in fish. Accordingly, exposure to 2 μg/kg AFB_1_ did result in liver lesions, but exposure to 20 and 200 μg/kg feed caused histopathological changes in carp liver (Svobodova and Piskac 1980). Severe hepatic changes have also been noted in Nile tilapia and rainbow trout at higher AFB_1_ exposure levels or when observing the animals after prolonged experimental duration (Ashley 1970; Tuan et al. 2002).

The age- and species-dependent differences in the sensitivity of different fish species to AFB_1_ are thought to be caused by differences in the metabolism of AFB_1_ in the liver (Ngethe et al. 1993; Santacroce et al. 2008). Especially a higher susceptibility of cold-water fish species compared with warm-water fish species has been proposed. For example, juvenile hybrid sturgeons should not be fed with more than 10 μg/kg AFB_1_ in the diet to avoid mortality (Rajeev Raghavan et al. 2011). In addition, cold-water species such as rainbow trout and European seabass (*Dicentrarchus labrax*) were considered to be more sensitive to AFB_1_ than channel catfish (Manning 2001; Tuan et al. 2002; El-Sayed and Khalil 2009). In comparison, beluga, *Huso huso*, showed only liver damage and reduced weight gain, but no increased mortality after 60 days of exposure to 75 and 100 μg/kg AFB_1_ (Sepahdari et al. 2010). In contrast, increased mortality was noted in Nile tilapia exposed to 200 μg/kg AFB_1_ (El-Banna et al. 1992). This shows that on the one hand, mortality is not a very reliable endpoint in fish. This is also confirmed by the LOEL calculations for mortality in fish in Fig. 6b.

#### Effects of ZEN on fish

**Fig. 7:**
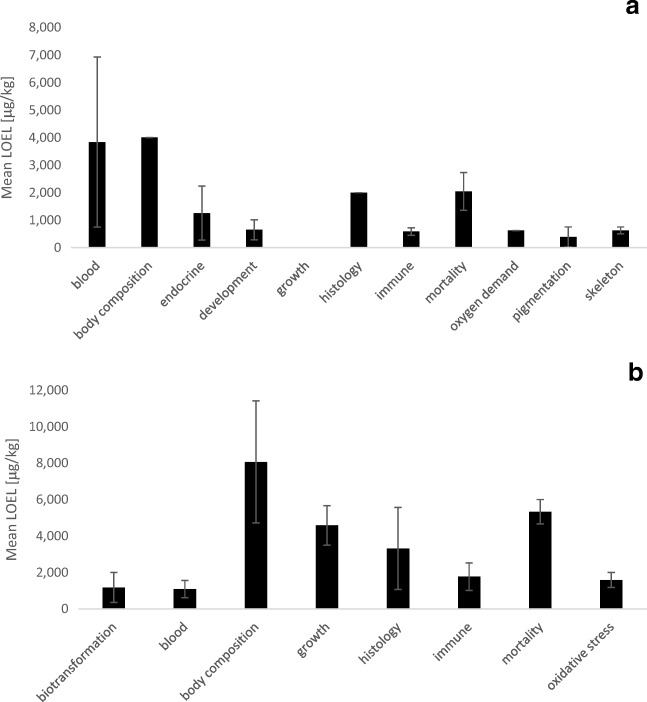
Mean lowest observable effect levels (LOEL) for different endpoints in different fish species **a** exposed to ZEN for different exposure durations (mean ± SEM). The number of data points from studies reported in the literature for each endpoint is as follows: blood *n* = 3, body composition *n* = 1, endocrine effects *n* = 10, development *n* = 4, growth *n* = 3, histology *n* = 1, immune responses *n* = 3, mortality *n* = 6, oxygen demand *n* = 1, pigmentation *n* = 2, skeleton *n* = 2; and the mean LOEL for different endpoints in different fish species **b** exposed to DON for different exposure durations (mean ± SEM). The number of data points from studies reported in the literature for each endpoint is as follows: biotransformation *n* = 2, blood *n* = 3, body composition *n* = 2, growth *n* = 6, histology *n* = 4, immune responses *n* = 4, mortality *n* = 3, oxidative stress *n* = 4. The studies that have been compiled for these graphs are listed in detail in Annex IV and V

ZEN in fish has been shown to be immunotoxic, genotoxic, hepatotoxic and cytotoxic and to cause increased damage to kidney tissue (Pietsch and Junge 2016; Pietsch 2017). Mycotoxins such as ZEN are known to contribute to the estrogenic potential in aquatic systems (Bucheli et al. 2005). Accordingly, estrogenic effects have also been described in several fish species (Johns et al. 2009; Schwartz et al. 2010; Bakos et al. 2013), although physiological effects are lacking in other species (Pietsch et al. 2015a, 2015b; Pietsch 2017). Estrogenic effects have therefore also been included in the calculations for Fig. 7a, which led to a mean LOEL for this endpoint of 1256 ± 982 μg/kg ZEN (mean ± SEM). The high estrogenic potency of ZEN has an impact on fish reproduction. For example, dietary exposure of carp to ZEN resulted in an impaired quality and number of sperm (Sándor and Ványi 1990). Developmental problems in zebrafish (*Danio rerio*) and early life stages of fathead minnow (*Pimephales promelas*) due to ZEN exposure have also been described (Johns et al. 2009; Schwartz et al. 2010; Bakos et al. 2013). Developmental effects often included the occurrence of edema and lacking pigmentation in early ontogenic life stages or impaired development of the bony parts of the body. Therefore, these endpoints have been summarised in Fig. 7a as the endpoint development, pigmentation, and skeleton. In addition, the effects of ZEN on fish growth have only been observed in water-exposed fish embryos, and due to the limited number of studies, a very low mean LOEL of 0.34 ± 0.31 μg/L ZEN (mean ± SEM) has been calculated for this endpoint. Moreover, effects on white blood cell counts of fish, leading to pronounced modulations of immune parameters, have been noted in carp fed ZEN-contaminated diets (Pietsch et al. 2015a). Accordingly, effects on immune responses due to ZEN exposure revealed a mean LOEL of 583 ± 163 μg/kg ZEN (mean ± SEM).

#### Effects of DON and NIV on fish

Until now, the effects of NIV on fish remain unknown. In contrast, the different effects of DON on fish have been described. DON feeding to cyprinids did not influence weight gain (Jorgensen 2012; Pietsch et al. 2014a, b), but salmonids and channel catfish showed reduction of feed intake and reduced growth and feed efficiency at dietary concentrations of DON of 1.0 to 8.8 mg/kg, resulting in a calculated mean LOEL for this endpoint of 4586 ± 1081 μg/kg DON (mean ± SEM, Fig. 7b). Significant histopathological changes in the liver related to dietary levels of DON were also reported resulting in a mean LOEL for this endpoint of 3317 ± 2252 μg/kg DON (mean ± SEM). Another typical response of fish to dietary DON exposure is the change of immune responses which occurred at a mean LOEL of 1767 ± 755 μg/kg DON (mean ± SEM). In addition, production of oxidative stress has already been shown for fish cell lines (Pietsch et al. 2011) but also occurs in vivo with a mean LOEL of 1588 ± 412 μg/kg DON (mean ± SEM, Fig. 7b).

#### Effects of OTA on fish

**Fig. 8:**
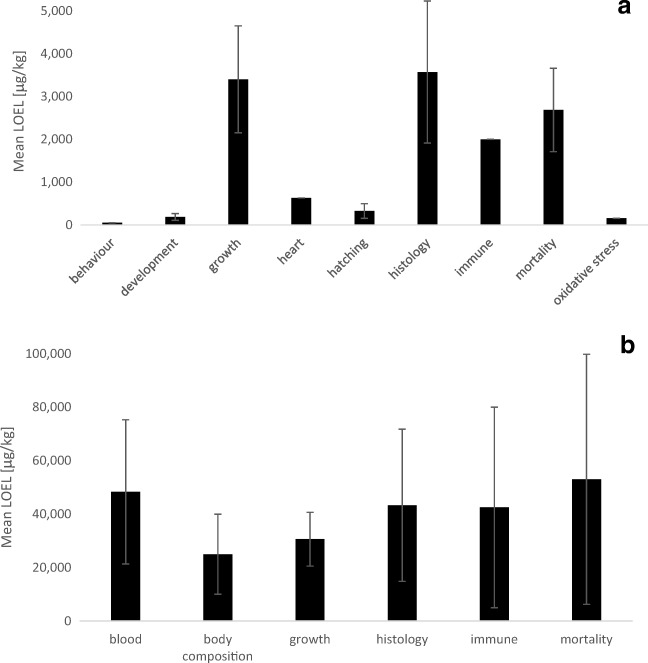
Mean lowest observable effect levels (LOEL) for different endpoints in different fish species **a** exposed to OTA for different exposure durations (mean ± SEM). The number of data points from studies reported in the literature for each endpoint is as follows: behaviour *n* = 1, development *n* = 4, growth *n* = 5, heart *n* = 1, hatching *n* = 3, histology *n* = 4, immune responses *n* = 2, mortality *n* = 9, oxidative stress *n* = 1; and the mean LOEL for different endpoints in different fish species **b** exposed to FB_1_ for different exposure durations (mean ± SEM). The number of data points from studies reported in the literature for each endpoint is as follows: blood *n* = 5, body composition *n* = 2, growth *n* = 9, histology *n* = 3, immune responses *n* = 2, mortality *n* = 5. The studies that have been compiled for these graphs are listed in detail in Annex IV and V

The bioavailability of OTA was found to be as low as 1.6% in fish (Hagelberg et al. 1989), but a number of severe effects of OTA exposure can still be observed in different fish species. Embryotoxicity including severe deformities, reduced growth and hatching rates, and increased embryo mortality has been reported in zebrafish, which was assumed to be related to the increased production of oxidative stress (Tschirren et al. 2018). Thus, developmental effects and impaired hatching of OTA-treated embryos showed a mean LOEL of 187 ± 80 and 327 ± 170 μg/L OTA (mean ± SEM, Fig. 8a), respectively. Moreover, histological damages to the kidneys and liver have been reported after exposure to OTA with a mean LEOL of 3573 ± 1659 μg/kg OTA (mean ± SEM, Fig. 8a). Damage to the heart appears to be a very sensitive endpoint in zebrafish embryos with a LOEL of 150 μg/L OTA, but needs confirmation in further fish species. The most sensitive endpoint appeared to be behaviour of sea bass in response to dietary exposure to OTA with a LOEL of 50 μg/kg OTA. In addition, dietary exposure to OTA has also resulted in reduced weight gain and lower feed conversion. This is also displayed by the mean LOEL for growth of 3400 ± 1249 μg/kg DON (mean ± SEM, Fig. 8a). In contrast, mortality shows an even lower mean LOEL of 2686 ± 974 μg/kg OTA (mean ± SEM).

#### Effects of FB_1_ on fish

The impact of fumonisins on fish has not been described in much detail so far. The biggest threat that may be posed to fish as a result of FB_1_ is to fish growth with a mean LOEL of 30.6 ± 10.1 mg/kg FB_1_ (mean ± SEM, Fig. 8b). However, FB_1_ is also known to have further adverse physiological effects on the kidney and liver, which is also displayed by the mean LOEL for the endpoint histology with a value of 43.3 ± 28.5 mg/kg FB_1_ (mean ± SEM). Furthermore, FB_1_ is able to interfere with the immune system, causing changes of disease resistance in fish with a mean LOEL of 42.5 ± 37.5 mg/kg FB_1_ (mean ± SEM). Effects on blood parameters, including significant decreases in haematocrit and changes of red and white blood cell populations, occurred in fish with a mean LOEL of 48.3 ± 27.0 mg/kg FB_1_ (mean ± SEM). Finally, increased mortality was observed in fish with a mean LOEL of 53.0 ± 46.8 mg/kg FB_1_ (mean ± SEM).

#### Effects of MON on fish

**Fig. 9:**
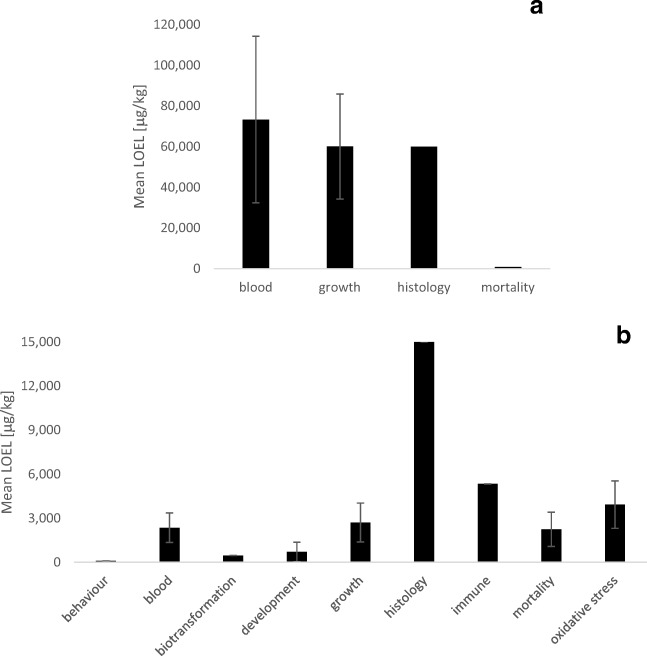
Mean lowest observable effect levels (LOEL) for different endpoints in different fish species **a** exposed to MON for different exposure durations (mean ± SEM). The number of data points from studies reported in the literature for each endpoint is as follows: blood *n* = 3, growth *n* = 5, histology *n* = 1, mortality *n* = 1; and the mean LOEL for different endpoints in different fish species **b** exposed to T-2 toxin for different exposure durations (mean ± SEM). The number of data points from studies reported in the literature for each endpoint is as follows: behaviour *n* = 1, biotransformation *n* = 1, blood *n* = 4, development *n* = 1, growth *n* = 4, histology *n* = 1, immune *n* = 1, mortality *n* = 3, oxidative stress *n* = 7. The studies that have been compiled for these graphs are listed in detail in Annex IV and V

Only a few effects of MON on fish have been described so far. These included effects on blood parameters with a mean LOEL of 73.3 ± 41.0 mg/kg MON, whereas growth in fish shows a mean LOEL of 45.0 ± 25.0 mg/kg MON (mean ± SEM, Fig. 9a). The study of Gonçalves et al. (2018) shows effects on MON on growth and survival of zebrafish after exposure to water-borne concentrations of less than 1 mg/L MON. This resulted in a mean LOEL of 60.1 ± 25.8 mg/kg MON for the effect on growth of all three fish species that have been investigated so far.

#### Effects of T-2 toxin on fish

Sublethal concentrations of T-2 toxin affected feed consumption and weight gain in different fish species with a mean LOEL of 2706 ± 1327 μg/kg T-2 (mean ± SEM, Fig. 9b). Effects on the antioxidant system have been observed at a mean LOEL of 3928 ± 1615 μg/kg T-2 (mean ± SEM) which is probably also related to the effects on lysosomal enzymes and the alkaline phosphatase (Kravchenko et al. 1989). In addition, T-2 toxin affects blood parameters, such as haematocrit and haemoglobin levels, with a mean LOEL of 2354 ± 1010 μg/kg T-2 (mean ± SEM). Increased mortality was observed after exposure to a mean T-2 concentration of 2242 ± 1160 μg/kg T-2 (mean ± SEM). However, the most sensitive endpoint appears to be behaviour, although this has only been reported for water-borne exposure to 93 μg/kg T-2 (Yuan et al. 2014).

#### Effects of ENN and BEA in fish

It has been shown that ENN can be present in fish tissues and is differently affected by food processing (Tolosa et al. 2017). Toxicity of ENN A has been investigated and impaired development of zebrafish embryos could be observed at concentrations of 1000 μg/L or higher (C. Pietsch, unpublished results).

### Estimation of species sensitivities

**Fig. 10:**
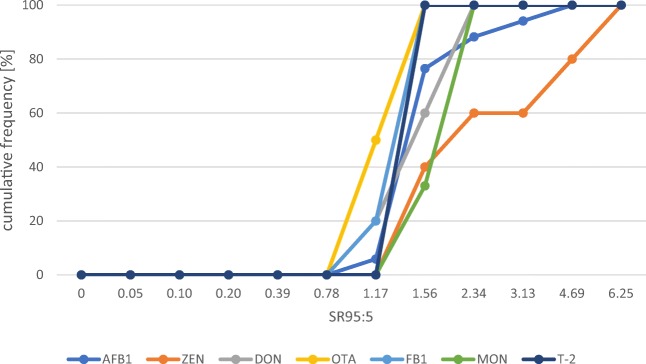
Fish species sensitivity to the different mycotoxins based on the toxicity reports from the literature listed in Annex IV and V comparing toxicity levels for AFB_1_ from beluga (*n* = 3), gibel carp (*n* = 3), catla (*n* = 4), channel catfish (*n* = 3), common carp (*n* = 7), hybrid tilapia (*n* = 5), rohu (*n* = 2), medaka (*n* = 2), mosquitofish (*n* = 2), Mozambique tilapia (*n* = 3), Nile tilapia (*n* = 31), rainbow trout (*n* = 39), red drum (*n* = 4), sea bass (*n* = 2), silver catfish (*n* = 3) and zebrafish (*n* = 6); for ZEN, the toxicity levels for Atlantic salmon (*n* = 2), common carp (*n* = 3), fathead minnow (*n* = 2), rainbow trout (*n* = 2) and zebrafish (*n* = 17) were compiled; for DON, toxicity levels for Atlantic salmon (*n* = 2), channel catfish (*n* = 2), common carp (*n* = 2), rainbow trout (*n* = 9) and zebrafish (*n* = 2) were used; for OTA, toxicity levels for channel catfish (*n* = 7), rainbow trout (*n* = 4), sea bass (*n* = 4) and zebrafish (*n* = 9) were compiled; for FB_1_, toxicity levels for channel catfish (*n* = 9), African catfish (*n* = 2), common carp (*n* = 4), mosquitofish (*n* = 2) and Nile tilapia (*n* = 3) were used; for MON, toxicity levels for channel catfish (*n* = 2), Nile tilapia (*n* = 3) and zebrafish (*n* = 2) were compared; and for T-2 toxin, the toxicity levels for channel catfish (*n* = 4), common carp (*n* = 5), rainbow trout (*n* = 5) and zebrafish (*n* = 3) were summarised; all values were calculated according to Elmegaard and Jagers op Akkerhuis (2000)

In general, for every chemical, a SR95:5 can be derived if a sufficient number of experiments have been conducted. However, in many cases, the number of species that have been tested is limited. The species sensitivity ratios SR95:5 revealed that two fish species have a sensitivity to AFB_1_ that is greater than 2 (i.e. sea bass and beluga). The fish species with the narrowest sensitivity range included the hybrid and Nile tilapia, catla and rainbow trout (with SR95:5 values close to 1). This resulted in the cumulative frequency curve as shown in Fig. 10. The calculations also revealed that the sensitivity ranges for this mycotoxin were not smaller for salmonid species than for the other groups of fish. The calculations for ZEN revealed that fathead minnow and rainbow trout are less sensitive to ZEN than the other species. The lower SR95:5 values for zebrafish and common carp exposed to DON indicate a smaller range of toxicity to this mycotoxin and thus a higher specificity in the responses of these fish species compared with the specificity of the responses in rainbow trout, Atlantic salmon and channel catfish. The cumulative frequencies of these sensitivities in Fig. 10 revealed that 60% of the species showed SR95:5 values lower than 1.5. The species sensitivity ratios SR95:5 for OTA indicated that all fish species have quite similar sensitivity to this mycotoxin. The comparison of the species sensitivity to FB_1_ shows that all fish species had SR95:5 values between 1.08 and 1.50, which strongly influenced the cumulative frequency in Fig. 10. The cumulative species sensitivity for fish exposed to MON reveals that two of the three fish species that have been investigated so far show SR95:5 values of more than 1.5. For T-2 toxin, all fish species that have been investigated so far show SR95:5 values between 1.19 and 1.28.

### Estimation of the risks for fish

**Fig. 11:**
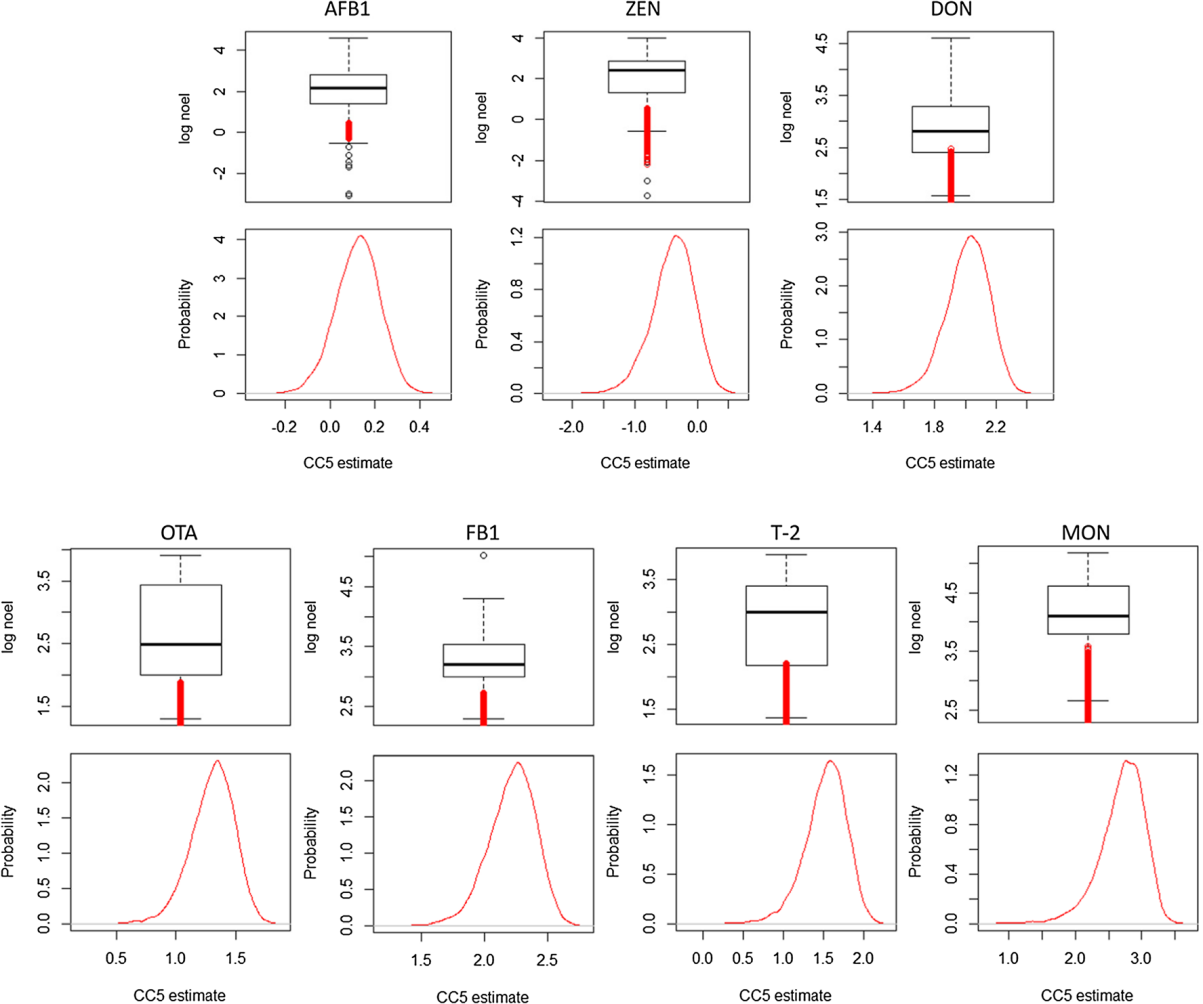
Fish predicted log NOELs as box plots including the critical concentration 5% (CC5) (in red colour) for fish exposed to the mycotoxins AFB_1_ (based on *n* = 247 data points for the CC5 calculation), ZEN (*n* = 51), DON (*n* = 39), OTA (*n* = 38), FB_1_ (*n* = 14), MON (*n* = 14) and T-2 toxin (*n* = 24) according to the Bayesian modelling, and plotting the derived probabilities for each calculation as kernel density plots (CC5 estimates as log 10[concentration of the toxin], see details in Tables S19 and S20 in Annex IV

The summary on the reported toxic levels of the seven relevant mycotoxins which were investigated in the present study shows that NOEL could be predicted for each of the mycotoxins and combined with the original NOEL that have been reported in the literature (Fig. S11 in Annex IV). The theoretical species sensitivity distribution can be achieved by illustrating the probability density functions of the NOEL for each mycotoxin. Although NOEL per se are defined as concentrations without an effect on an organism, critical threshold levels affecting 5% of a fish population must be assumed and their probabilities have been calculated and displayed in Fig. 11. The detailed results show that for AFB_1_, a potential risk for 5% of the fish in a population would occur at concentrations 1.69–8.70 μg/kg feed (min–max, with a mean of 4.30 μg/kg feed, Table S19 in Annex IV). This was also the most reliable prediction since the aggregation of data on the effects of AFB_1_ contained the highest number of data points (*n* = 247). For ZEN, the range of CC5 estimates would be between 0.0002 and 0.5295 μg/kg feed (min–max) with a mean of 2.30 μg/kg feed. For DON, this range was calculated to be between 23.8 and 272.3 μg/kg feed (min–max) with a mean of 114.8 μg/kg feed. For OTA, the CC5 was estimated to range between 0.491 and 1.892 μg/kg feed (min–max) with a mean of 1.324 μg/kg feed. Toxins for which considerably less data points have been accumulated, the predictions showed higher variation. Accordingly, for the toxins FB_1_ and MON, CC5 estimates ranging from 6.23 to 3867 μg/kg feed (min–max, with a mean of 505.7 μg/kg feed) and 0.11 to 2040 μg/kg feed (min–max, with a mean of 222.7 μg/kg feed) were obtained, while T-2 toxin showed a range of 0.47–142.4 μg/kg feed (min–max, with a mean of 21.9 μg/kg feed). In the case that the actual toxin concentrations in fish feeds exceed the estimated range of the CC5 derived from data from laboratory studies, a potential risk for at least 5% of the fish can be assumed. Accordingly, a risk can be assumed for AFB_1_ intoxications in the maximum contamination scenario and the bad-corn and bad-wheat scenario. The estimated CC5 values for ZEN, DON, FB_1_, MON and T-2 would be exceeded by the estimated toxin levels in feed in all scenarios. Similarly, the estimated CC5 values for OTA would be exceeded by the estimated toxin levels in feed in all scenarios except the median contamination scenario.

## Discussion

The present study gives an estimation on the potential contamination of fish feeds with several mycotoxins which includes the so-called emerging mycotoxins in the assessment since these may be increasingly important in the future.

### Comparison of the mycotoxin contamination scenarios

The present study not only concentrated on the commonly detectable mycotoxins in feed ingredients and feeds, but also on emerging mycotoxins. Up to our current knowledge, the mean contamination scenario appears to yield realistic contamination values if a feed producer avoids using feed ingredients with obvious signs of contamination. Although DDGS is a feed ingredient with a higher risk of mycotoxin contamination, it is rarely used at high percentages in fish feeds. More commonly used feed ingredients that can be highly contaminated are wheat and corn. For this reason, the bad-wheat and the bad-corn scenario have been calculated as well which resulted in high mycotoxin levels in the feeds. In addition to the high levels of ENN and BEA, this also mainly concerned DON, MON and FB_1_. This may be an indicator of which mycotoxins feeds should be screened for in final feeds if a pre-screening in the raw materials is not performed by a feed producer. According to Ivic et al. (2009), wheat and corn products are contaminated with higher amounts of *Fusarium* toxins than soybean and pea. Although the data sets for each of these ingredients do not have the same size, the present calculations support this assumption.

Aflatoxin concentrations in actual fish feeds appeared to be rather low (summarised in Fig. 4). Not included were data from a survey in which aflatoxins were found in 21 out of 31 feed samples, whereby AFB_1_ reached a maximum level of 221 μg/kg (Gonçalves 2016). Thus, critical concentrations for fish health may be reached in some cases. Similarly, the OTA concentrations appear to be rather low in most of the fish feeds that were compiled for Fig. 4, but may reach high OTA levels under unfavourable storage conditions which may pose a risk to fish (Tschirren et al. 2018). In addition, thermal stability of ENN in fish products has been investigated by Tolosa et al. (2017) showing that food processing affects the presence on ENN in fish tissue variably. The contamination calculations for the 97 fish feed in the present study appear to overestimate the actual concentrations of ENN and BEA since the toxin content of the raw materials is assumed to also be present in the final feeds. Thus, more research on these emerging mycotoxins is highly recommended.

In most cases, the possible feed contaminants are already present in the field at higher abundance than in storage. In addition, several factors influence the formation of mycotoxins in feed ingredients which have not been understood in total so far. For example, damage to cereal grains increases the possibility of fungal infections leading to toxin production in the grains (Lacey and Magan 1991). But the toxin production by fungi is also determined by their genetic capability to produce certain chemical compounds and environmental factors. Toxin production depends on several factors including physical, chemical and biological factors, and each fungus requires special conditions for its growth and other conditions for its toxin production. The physical factors include temperature, moisture and light conditions. The optimal temperature for the production of individual toxins by *F. graminearum* on soybeans was found to differ with the temperature (Garcia et al. 2012). In addition, fungi commonly need at least 1–2% oxygen for their growth, whereas the CO_2_ concentrations for optimal fungal development differ widely between fungal species (Taniwaki et al. 2009). The effects of light on fungal growth and mycotoxin production are often contradicting and need further research for clarification (Mohsen et al. 2015; Cheong et al. 2016).

In addition to physical factors, also the chemical environment (pH, nutrients availability, feed preservatives) determines fungal development. The availability of nutrients that are essential for fungal growth (i.e. carbohydrates, nitrogen-containing compounds and microelements such as copper, zinc and cobalt) and the presence of other chemicals can influence the development and sporulation of the fungi and their toxin production (Montville and Shih 1991; Škrinjar et al. 1995).

Furthermore, certain biological factors appear to influence the occurrence of mycotoxins. The presence of bacteria or fungi at the same time can impair the growth of mycotoxin-producing fungi and the resulting production of mycotoxins. Accordingly, an antagonistic interaction between *Alternaria* and *Aspergillus* species has been observed (Lee and Magan 1999) but has not been investigated in animal feeds so far.

### Is mycotoxin contamination in feeds a problem for farmed fish?

Generally, our knowledge on the effects of mycotoxins on fish is insufficient, and further research is needed to estimate the actual impact of mycotoxins on fish health in aquaculture. Most of the contamination scenarios that have been calculated in the present study yielded mycotoxin levels in fish feeds that may pose a risk to fish. This emphasises that mycotoxins may be a realistic threat to fish health in aquaculture. However, certain uncertainties influenced the estimations in the present study and will be discussed in the following sections.

### Uncertainties

As already mentioned in the “Methods” section, several studies did not report the LOD and LOQ values for the methods used, and the measured values might contain uncertainties depending on the sample preparation and the detection method that has been chosen. In addition, it can be assumed that the actual mycotoxin exposure concentrations in feed ingredients, feeds and fish are underestimated since masked mycotoxins often cannot be detected by routine measurement techniques. For most feed compounds, matrix effects have not sufficiently been tested, which may further impair accurate detection of the different mycotoxins. The advantages and disadvantages of traditional and emerging methods for mycotoxin analysis have been reviewed by Pascale (2009). Research continuously improves the detection methods for mycotoxins, but the high number of different mycotoxins with different chemical characteristics and the masking by the sample material make the exact detection of these substances rather complicated. In addition, metabolites of commonly occurring mycotoxins should be investigated more in detail in contaminated cereals, since, e.g. DON derivatives such as 3-acetyl DON and 15-acetyl DON can occur in significant amounts with DON (Mirocha et al. 1989). Another problem with the present data sets is that the toxin levels in the control diets in several studies have been assumed to be zero, but toxin analyses have not been performed sufficiently to support this assumption. Thus, the true value may correspond to a concentration considerably above zero thus underestimating the control level.

What also makes mycotoxin research difficult is the fact that we do not know enough about mycotoxin mixtures and their effects. Natural contamination of feed ingredients leads to the occurrence of several mycotoxins at the same time and their interactions remain mostly unknown. Some mixture effects have been investigated in higher vertebrates, but rarely in fish (D’Mello et al. 1999; Tuan et al. 2003; Hooft et al. 2019). Combinations of mycotoxins can have serious effects. One example is the exposure of chicken embryos to fusaric acid and FB_1_, while these mycotoxins had no effect on mortality if applied separately (D’Mello et al. 1999). In addition, MON caused mortality in broilers and the MON effects could be enhanced by the addition of FB_1_. Accordingly, Tuan et al. (2003) showed differences in the sensitivity of channel catfish and Nile tilapia to FB_1_ and MON. Furthermore, rainbow trout appear to be more sensitive to DON than Nile tilapia (Hooft et al. 2019). Generally, interactions of mycotoxins may have antagonistic, agonistic and synergistic characteristics (Sobral et al. 2018). As a consequence, if several mycotoxins occur in the diet, the observed effects on the treated animals cannot be assigned to the presence and action of a single mycotoxin in this mixture, since the effects of different mycotoxins in these experiments might be too unspecific or overlapping with each other. Up to now, it has been assumed that synergism among co-occurring mycotoxins is quite frequent, but the resulting consequences for the animals remain largely unknown (D’Mello et al. 1999). Nevertheless, these problems apply to mycotoxin research in general and the resulting constraints for research have to be accepted up to a certain extent at the moment. This shows that this area requires additional research to facilitate better assessment of the risks to animal production.

Irrespective of the toxic chemical that is used, it is commonly observed that species vary considerably in their sensitivity to the chemicals. In general, some biological endpoints such as cancer caused by AFB_1_ exposure or endocrine interactions due to ZEN exposure occur in higher as well as in lower vertebrate such as fish. Other effects such as the effects of ENN A on the early development appear to be more specific to fish. The differences in sensitivity are often thought to be caused by species-specific differences in morphology or metabolisation. For example, terrestrial animals are capable of hydrolysing T-2 toxin to HT-2 toxin, but this biotransformation pathway appears not to be relevant in carp (Wu et al. 2014). This may indicate why differences in sensitivity to these mycotoxins in fish may occur in comparison to terrestrial animals and emphasises the importance of mycotoxin research in fish. However, between different fish species, considerable differences in sensitivity also have been observed, although the exact difference in biotransformation processes have not been described in sufficient detail (Hooft et al. 2019). The common variation of species in sensitivity raises the question whether the most sensitive fish species have already been investigated in the past studies which would be important for proper risk assessment. In order to investigate this, the sensitivity ranges of the species have been calculated in the present study. Fish appear to be very sensitive to AFB_1_ and OTA, but also to ZEN. However, the results for ZEN sensitivity may have considerably been overestimated since water-borne exposure data have been combined with feed-borne toxicity data for these calculations. It was not possible to perform the calculations for exposure to ZEN via water and feed separately, since not enough studies have investigated the effects of dietary ZEN exposure in different fish species so far. Compared to the SR95:5 values for different pesticides in the study of Elmegaard and Jagers op Akkerhuis (2000), the specificity of the reaction of fish to mycotoxins is rather high since they do not exceed values of 7. In addition, the present sensitivity calculations do not support the common assumption that cold-water species are more sensitive, for example, to AFB_1_ or DON than warm-water fish species. This is a unique insight into mycotoxin action that cannot be investigated in mammals. Thus, it would be important to clarify the reasons for fish species-dependent differences in mycotoxin toxicity by further detailed research.

The investigation of SR95:5 values also allows the safety factors that should be used for each mycotoxin to be estimated, since they are a ratio of the effect levels of 95% of the species to the 5% most sensitive species. According to the present predictions, a safety factor of 7 to 10 would be sufficient to correct the predictions of safe toxin concentrations for species-specific differences with regard to the investigated mycotoxins.

The assumed feed contamination in commercial fish feeds leads to health problems in fish, although the discussed uncertainties due to a lack of data on toxicity in fish of different mycotoxins and the effects of mycotoxin mixtures render these estimations imprecise to a certain extent. As another approach, the present study used CC5 values to estimate the risk for 5% of a fish population. These calculations showed a risk for the fish in most feed contamination scenarios. But not surprisingly, the confidence limits for these calculations of hazardous concentrations depend heavily on the number of species tested which is why there is still a considerable level of uncertainty within the present calculations. A strong influence of the number of species tested on the risk estimation can also be seen from the study of Luttik and Aldenberg (1997) for other groups of species, such as birds and mammals. To prevent damage from fish populations, the reported mean CC5 values should be used as recommended maximum levels for feed. However, due to the above-mentioned restrictions, these should be revised as soon as possible, especially for ZEN, FB_1_ and MON.

If taking up mycotoxin-contaminated feeds the fish may not show health issues, but the retention of mycotoxins in edible parts of the fish can also be problematic for consumers (Pietsch 2019). Thus, the mycotoxin contamination of feed ingredients is a problem that increasingly needs to be addressed by crop farmers, feed producers, fish farmers and authorities. Avoiding heavily contaminated raw materials or feed ingredients with a higher risk of mycotoxin contamination in general improves the feed quality by lowering the potential mycotoxin contamination levels. For some mycotoxins, the stability during storage and feed production processes have not yet been described in sufficient detail, which should be improved by future research. This would allow for more realistic risk assessments.

From the toxicity data, it was possible to estimate hazardous concentration ranges for each mycotoxin and to recommend safety factors that should be used to correct for species-specific differences in sensitivity. The results showed that for most of the mycotoxins that have been investigated, the estimated feed contamination levels exceeded the critical levels and it can be assumed that a risk for fish is present. The present study also confirms that further research is necessary to gain detailed information about species-specific sensitivity to mycotoxins and to derive reliable recommendations for maximum mycotoxin levels in fish feeds.

## Electronic supplementary material

ESM 1 (DOCX 26 kb)

ESM 2 (DOCX 249 kb)

ESM 3 (DOCX 47 kb)

ESM 4 (DOCX 227 kb)

ESM 5 (DOCX 48 kb)

ESM 6 (DOCX 18 kb)
