# Supplementary material for: Risk assessment for mycotoxin contamination in fish feeds in Europe
Source: Mycotoxin Res. 2019 Jul 26;36(1):41–62. doi: 10.1007/s12550-019-00368-6 (PMC6971146; doi:10.1007/s12550-019-00368-6)
Supplement: Supplementary file 1 — (DOCX 26 kb) [file 12550_2019_368_MOESM1_ESM.docx]

Annex I: Nutrient composition of feed ingredients

**Table S1** Details for nutrient concentrations in feed ingredients (supplied by Provimi Kliba AG, Kaiseraugst, Switzerland).

| **Feed ingredient** | Nutrient Composition | | | | |
| --- | --- | --- | --- | --- | --- |
|  | Dry matter | Ash | Crude protein | Crude lipid | Crude fibre |
| Casein | 91.5 | 3.7 | 86.5 | 0.2 | 1.0 |
| Blood meal (batch) | 89.0 | 4.0 | 76.7 | 1.0 | 1.0 |
| Fish meal | 90.3 | 12.4 | 72.9 | 13.3 | 2.2 |
| Krill meal | 92.9 | 13.0 | 58.0 | 18.0 | 6.0 |
| Poultry offal meal | 90.0 | 8.0 | 60.0 | 14.0 | 1.9 |
| Canola | 91.0 | 6.8 | 39.0 | 4.0 | 11.9 |
| Chick pea | 90.8 | 3.3 | 20.6 | 4.4 | 7.7 |
| Gluten (corn) | 92.9 | 1.7 | 63.1 | 8.0 | 1.9 |
| Soybean meal (46-expel) | 89.0 | 6.0 | 42.0 | 3.8 | 6.5 |
| Yeast (brewers) | 94.7 | 9.2 | 48.5 | 3.0 | 1.9 |
| Barley | 89.8 | 2.4 | 10.7 | 1.8 | 7 |
| Corn (7.5% CP) | 87.7 | 1.2 | 8.3 | 4.0 | 2.4 |
| Dextrose | 95.0 |  |  |  |  |
| Dried Distillers Grains Soluble (DDGS) | 95.0 | 3.7 | 35.0 | 6.2 | 10.0 |
| Soybean grits | 88.0 | 6.5 | 42.0 | 2.0 | 7.0 |
| Rapeseed expelled | 88.0 | 6.3 | 32.4 | 11.8 | 11.7 |
| Sunflower seed cake | 88.0 | 6.9 | 23.2 | 9.8 | 22.3 |
| Field beans | 88.0 |  | 26.0 |  | 7.8 |
| Trace mineral premix | 90.0 | 38.0 | 5.0 | 0.5 | 4.0 |
| Vitamin premix | 90.0 | 5.3 | 13.5 | 3.9 | 3.0 |
| Binder (inert) | 90.0 | 5.0 | 0 | 0.0 | 0 |
| Vegetable oil | 100 | 0 | 0 | 100 | 0 |
| Canola oil | 100 | 0 | 0 | 100 | 0 |
| Fish oil | 100 | 0 | 0 | 100 | 0 |
| Wheat bran | 88.7 | 5.3 | 11.3 | 2.2 | 7.6 |
| Wheat (12 CP) | 89.0 | 0.6 | 12.0 | 1.7 | 0.8 |
| Lecithin - soy (70%) | 97.0 | 0.0 | 0 | 70.0 | 0 |
| Poultry feather meal | 89.2 | 5.4 | 77.9 | 4.2 | 0.6 |

Figure S1. Calculated fat and protein content of estimated composition of 97 commercial fish feeds.

Figure S2 Calculated percentages of feed ingredients in 97 commercial fish feeds, mean ± SEM, n = 97.
