# Supplementary material for: Risk assessment for mycotoxin contamination in fish feeds in Europe
Source: Mycotoxin Res. 2019 Jul 26;36(1):41–62. doi: 10.1007/s12550-019-00368-6 (PMC6971146; doi:10.1007/s12550-019-00368-6)
Supplement: Supplementary file 2 — (DOCX 249 kb) [file 12550_2019_368_MOESM2_ESM.docx]

Annex II: Estimated mycotoxin contamination of feed ingredients


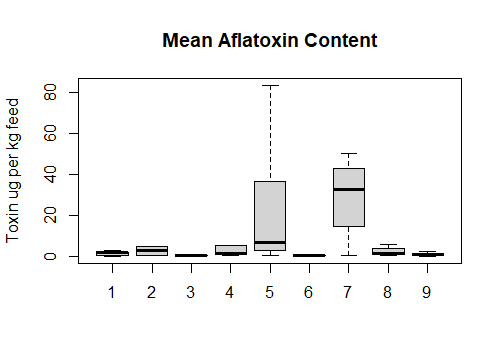


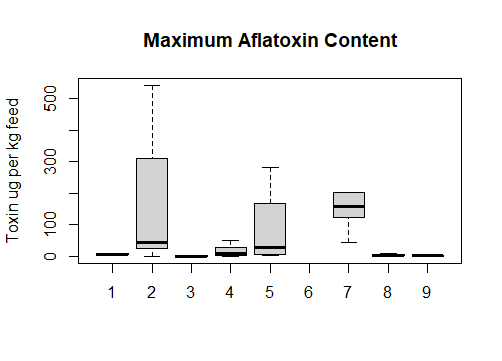


**Figure S1.** Mean (upper graph) and maximum (lower graph) content of aflatoxin B_1_ in feed ingredients (excluding outliers) according to the literature (listed in Annex III). 1 = wheat. 2 = corn, 3 = barley, 4 = soya, 5 = sunflower, 6 = canola, 7 = beans, 8 = DDGS, 9 = meat.

**Table S2** Literature data for aflatoxin B_1_ (AFB_1_) in feed ingredients (μg/kg), average values for all reported studies and the percentage of positive tested samples are shaded in light blue.

| **AFB_1_ in wheat** | | | | | | | | |
| --- | --- | --- | --- | --- | --- | --- | --- | --- |
| **1.34** | | **9.48** | |  | **34.1 %** | | |  |
| mean | | max | | min | N (pos/all) | | | References |
|  | | 10 | | 1 | 9/80 | | | Martins et al. 2008 |
| 3 | | 6 | | 2 | 3/3 | | | Griessler et al. 2010 |
| 2  0 | | 6  20 | |  | 7/15  6/122 | | | Rodrigues and Naehrer 2012 |
| 0.4 | |  | |  | 6/? | | | EFSA CONTAM Panel 2014 |
| 1.7 | | 5.4 | |  | 15/201 | | | Pleadin et al. 2015 |
| **AFB_1_ in corn** | | | | | | | | |
| **7.42** | **717** | |  | | | **28.8 %** |  | |
| mean | max | | min | | | N (pos/all) | References | |
| 4.4 |  | |  | | | 83/110 | Piva et al. 2006 | |
|  | 45 | | 1 | | | 30/248 | Martins et al 2008 | |
|  | 311 | |  | | | 3/? | Binder et al. 2007 | |
| 0.42 |  | |  | | | 1/? | Mage et al. 2007 | |
| 3.4 | 4.5 | | 2.7 | | | 4/12 | Segvić Klarić et al. 2009 | |
|  | 0.5 | | 0.1 | | | 8/95 | Soares and Venancio 2011 | |
| 5 | 6 | | 1 | | | 3/15 | Griessler et al. 2010^a^ | |
| 3 | 39 | | 1 | | | 6/16 |  | |
| 3.2 | 42.6 | |  | | | 8/21 | Tabuc et al. 2011 | |
|  | 45 | | 1 | | | 30/248 | Martins et al. 2008 | |
| 0.28 |  | |  | | | 1/28 | Grajewski et al. 2012 | |
| 0.61 |  | |  | | | 1/14 |  | |
| 1 | 26 | |  | | | 4/50 | Rodrigues and Naehrer 2012 | |
| 38 | 6105 | |  | | | 409/1077 |  | |
| 37 | 542 | |  | | | 15/30 |  | |
| 0.6 |  | |  | | | 62/127 | EFSA CONTAM Panel 2014 | |
| 1.6 |  | |  | | | 16/? |  | |
| 38.46 | 2072 | |  | | | 305/972 | Pleadin et al. 2015 | |
| 0.18 |  | |  | | | 1/8 | Kosicki et al. 2016 | |
|  | 88.8 | | 1.3 | | | 103/180 | Hajnal et al. 2017 | |

a = reported only means of all samples

| **AFB_1_ in barley** | | | | |
| --- | --- | --- | --- | --- |
| **0.51** | **2.33** |  | **43.4 %** |  |
| mean | max | min | N (pos/all) | References |
| 0.11 | 0.24 |  | 21/21 | Ibáñez-Vea et al. 2012 a |
| 0.13 | 0.22 |  | 40/40 |  |
| 0.14 | 0.28 |  | 30/30 |  |
| 0.13 | 0.34 |  | 14/14 |  |
|  | 10 | 1 | 5/74 | Martins et al. 2008 |
|  | 2 |  | 1/24 | Rodrigues and Naehrer 2012 |
| 1.54 | 3.22 |  | 9/147 | Pleadin et al. 2015 |

| **AFB_1_ in beans** | | | | |
| --- | --- | --- | --- | --- |
| **21.45** | **146** |  | **63.0 %** |  |
| mean | max | min | N (pos/all) | References |
| 35.73 | 158.6 | 0.3 | 17/27 | Beuchat-Leuchowich 1970 |
| 29.18 | 203.3 | 0.1 | 17/27 |  |
| 50.42 | 202.4 | 0.1 | 17/27 |  |
|  | 122 | 5.3 | 9/? | Bankole et al. 1996 |
|  | 42.3 | 7.7 | 7/? |  |
| 0.38 |  |  | 2/? | EFSA CONTAM Panel 2014 |

| **AFB_1_ in sunflower products** | | | | |
| --- | --- | --- | --- | --- |
| **35.53** | **123** |  | **70.3 %** |  |
| mean | max | min | N (pos/all) | References |
| 0.4 |  |  | 2 | EFSA CONTAM Panel 2014 |
| 1.7 | 8.8 | 0.1 | 40/125 | Mortensen and Granby 2014 |
| 5.14 | 13.8 | 1.5 | 5/7 | Mmongoyo et al. 2017^b^ |
| 19.7 | 52.8 | 3.2 | 4/7 |  |
| 209.43 | 598.4 | 7.1 | 4/7 |  |
| 148.97 | 536 | 2.7 | 6/6 |  |
| 5.13 | 12 | 1.5 | 3/7 |  |
| 7.1 | 20.3 | 1.4 | 9/9 |  |
| 6.45 | 11.2 | 1.7 | 2/5 |  |
| 1.75 | 2.3 | 1.4 | 4/7 |  |
| 39.43 | 217.6 | 1.6 | 6/7 |  |
| 1.8 | 2 | 1.6 | 2/7 |  |
| 244.17 | 662.7 | 2.8 | 3/6 |  |
| 6.7 | 28.6 | 1.5 | 6/7 |  |
| 23.69 | 174.2 | 1.4 | 8/9 |  |
| 2.63 | 3.7 | 1.9 | 3/5 |  |
| 56.24 | 162 | 1.8 | 5/6 |  |
| 54.96 | 261.8 | 1.4 | 5/6 |  |
| 83.5 | 280.6 | 1.7 | 5/7 |  |
| 2.7 | 1.9 | 1.6 | 2/5 |  |
|  |  | 1.4 | 1/7 |  |
| 2.4 | 2.7 | 2.1 | 3/4 |  |
| 6 | 17.8 | 1.7 | 7/7 |  |
| 13.2 | 34.3 | 2 | 6/6 |  |
| 33.53 | 88.2 | 1.9 | 7/7 |  |
| 13.45 | 31.9 | 2.2 | 4/5 |  |
| 2.9 | 5.3 | 1.7 | 7/7 |  |
| 33.62 | 97.7 | 3 | 6/6 |  |
| 1.9 | 2.2 | 1.5 | 4/5 |  |

^b^ = these data have been obtained from outside of Europe.

| **AFB_1_ in soybean products** | | | | |
| --- | --- | --- | --- | --- |
| **3.48** | **17.7** |  | **26.7 %** |  |
| mean | max | min | N (pos/all) | References |
|  | 14 | 7 | 2/1046 | Nesheim and Wood 1995 |
|  | 0.41 |  | 32/55 | Valenta et al. 2002 |
| 5.37 |  |  | 2/7 | Kokic et al. 2009 |
|  | 10 | 1 | 2/66 | Martins et al. 2008 |
| 13.3 |  |  |  | Deng et al. 2010 |
|  | 51.7 |  | ?/12 | Tabuc et al. 2011 |
| 1 | 2 |  | 4/7 | Rodrigues and Naehrer 2012 |
| 1 | 42 |  | 28/159 |  |
| 0.32 |  |  | 30/? | EFSA CONTAM Panel 2014 |
| 1.88 | 4.02 | 0.5 | 5/23 | Fapohunda et al. 2018 |

| **AFB_1_ in DDGS** | | | | |
| --- | --- | --- | --- | --- |
| **4.19** | **15.5** |  | **13.5 %** |  |
| mean | max | min | N (pos/all) | References |
| 1.18 | 1.21 | 1.15 | 2/16 | Zhang et al. 2009 ^b^ |
| 1.83 | 2.56 | 1.04 | 3/69 |  |
| 2.2 | 3.7 | 1.1 | 6/20 |  |
| 1 | 89 |  | 18/393 | Rodrigues-Chin 2011 |
| 1.29 | 2 | 1 | 13/59 | Zhang and Caupert 2012 ^b^ |
| 20 |  |  | 62/1238 | Schatzmayr and Streit 2013 |
| 0.2 | 0.3 | 0.1 | 2/13 | Mortensen and Granby 2014 |
| 5.8 | 9.9 | 1 | 21/150 | Abudabos et al. 2017 |

^b^ = these data have been obtained from outside of Europe.

| **AFB_1_ in rapeseed products** | | | | |
| --- | --- | --- | --- | --- |
| **0.39** | **?** |  | **100** |  |
| mean | max | min | N (pos/all) | References |
| 0.39 |  |  | 6/? | EFSA CONTAM Panel 2014 |

| **AFB_1_ in chicken meat** | | | | |
| --- | --- | --- | --- | --- |
| **0.91** | **1.76** |  | **43.9 %** |  |
| mean | max | min | N (pos/all) | References |
| 0.11 |  |  |  | Richard et al. 1986 |
| 0.14 |  |  |  |  |
| 0.37 | 0.49 | 0.23 | 3/8 | Hussain et al. 2010 |
| 0.67 | 0.77 | 0.48 | 3/8 |  |
| 1.63 | 2.18 | 0.65 | 3/8 |  |
| 0.33 | 0.41 | 0.25 | 2/6 |  |
| 0.41 | 0.49 | 0.32 | 3/6 |  |
| 1.35 | 1.9 | 0.95 | 5/6 |  |
| 0.86 | 1.63 | 0.22 | 3/8 |  |
| 1.02 | 1.9 | 0.24 | 3/8 |  |
| 2.4 | 3.27 | 0.95 | 4/8 |  |
| 0.63 |  |  |  | Herzallah 2013 |
| 1.19 | 4.41 |  | 15/39 | Iqbal et al. 2014 |
| 0.89 | 1.89 |  | 18/45 |  |

^b^ = these data have been obtained from outside of Europe.

| **AFB_1_ in fishmeal** | | | | |
| --- | --- | --- | --- | --- |
| **1.6** | **?** |  | **100** |  |
| mean | max | min | N (pos/all) | References |
| 1.6 |  |  | ? | Deng et al. 2010 |


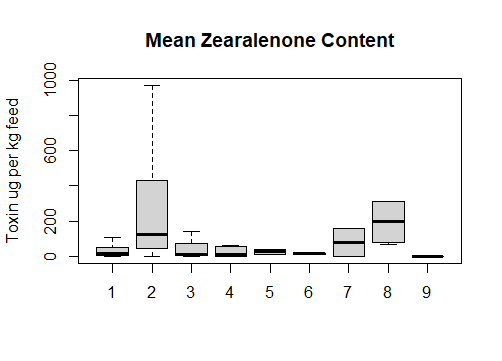


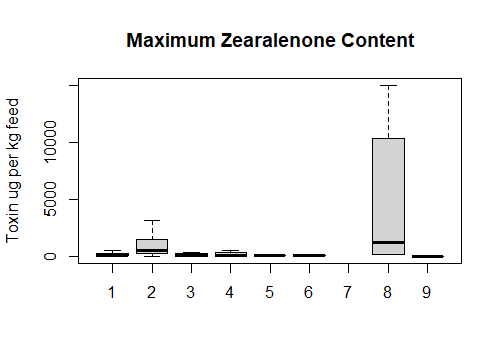


**Figure S2.** Mean (upper graph) and maximum (lower graph) content of zearalenone in feed ingredients (excluding outliers) according to the literature (listed in Annex III). 1 = wheat. 2 = corn, 3 = barley, 4 = soya, 5 = sunflower, 6 = canola, 7 = beans, 8 = DDGS, 9 = meat.

**Table S3** Literature data for zearalenone (ZEN) in feed ingredients (μg/kg), average values for all reported studies and the percentage of positive tested samples are shaded in light blue.

| **ZEN in wheat** | | | | |
| --- | --- | --- | --- | --- |
| **33.87** | **492** |  | **46.8 %** |  |
| mean | max | min | N (pos/all) | References |
|  | 182 | 61 | 7/? | Bartos and Matyas 1981 |
| 80 | 1560 |  | 61/? | Gleissenthal et al. 1989 |
|  | 1560 |  | 58% | Lepschy-von Gleissenthal et al. 1989 |
| 27 | 64 | 4 | 7/? | Ranfft et al. 1990 |
| 45 | 174 | 2 | 7/? | Tanaka et al. 1990 |
| 22 | 32 | 12 | 2/? | Hietaniemi and Kumpulainen 1991 |
| 178 | 8036 |  | 80% | Müller and Schwadorf 1993b |
| 17 | 120 |  | ?/97 | Vrabcheva et al. 1996 |
| 178 | 8036 | 1 | 67/? | Müller et al. 1997^a^ |
| 3 | 6 | 3 | 11/? |  |
| 5 | 15 | 1 | 9/? |  |
| 20 | 109 | 1 | 10/? |  |
| 4 | 20 | 1 | 15/? |  |
| 11 | 52 | 2 | 28/? |  |
| 5 |  |  | 7/? | Eriksen and Alexander 1998 |
|  | 170 |  | 25/25 | Curtui et al. 1998 |
| 210.3 | 890 | 50 | 215/367 | Rafai et al. 2000 |
|  | 860 | 11 | 22/24 | Schneweis et al. 2002 |
|  | 2 | 1 | 3/28 | Schollenberger et al. 2002 |
|  | 8 | 1 | 4/13 | Schollenberger et al. 2002 |
|  | 24 | 2 | 15/19 | Schollenberger et al. 2002 |
|  | 2 |  | 10/30 | Rasmussen et al. 2003 |
| 19000 |  |  | ? | Pussemier et al. 2006 |
| 187 | 921 |  | 44/48 | Binder et al 2007 |
|  | 184 | 2.5 | 8/? | Mage et al. 2007 |
|  | 95.6 |  | 16/51 | Mankevičienė et al. 2007 |
|  | 33.4 |  | 16/49 |  |
|  | 28.1 |  | 33/48 |  |
|  | 45.8 |  | 33/34 |  |
|  | 1292 |  | 309/1624 | Edwards 2009 |
| 35.7 |  |  | 1/1 | Kokic et al. 2009 |
|  |  | 10 | 1/54 | Manova and Mladenova 2009 |
|  |  | 27 | 1/7 | Cunha and Fernandes 2010 |
| 156.5 | 200 | 113 | 2/33 | Giraud et al. 2010 |
| 57 |  |  | 1/25 | Griessler et al. 2010 ^a^ |
|  | 50 | 13 | 4/6 | Klarić et al. 2009 |
| 108 | 155 |  | 2/? | Monbaliu et al. 2010 |
|  | 67.3 | 36.7 | 6/12 | Stroia et al. 2010 |
|  | 5.52 |  | 4/40 | Banu et al. 2011 |
| 2.225 | 3.57  41.8 | 0.88  0.41  0.42 | 2/2  5/16  1/1 | Galbenu et al. 2011a |
|  | 6 | 3 | 4/40 | Banu et al. 2011 |
| 110 | 490 | 4 | 35/35 | Chelkowski et al. 2012 |
| 53 | 145 |  | 14/208 | Rodrigues and Nährer 2012 |
| 43 | 513 |  | 62/184 |  |
|  | 21.1 | 1.9 | 5/15 | Škrbić et al. 2012 |
|  | 143 | 10 | 22/28 | Stankovic et al. 2012 |
|  | 201 | 16 | 71/75 |  |
|  | 310 |  | 27/312 | Van Der Fels-Klerx et al. 2012 |
|  | 1000 | 37.6 | 18/26 | Alexa et al. 2013 |
|  | 105.6 | 28 | 20/26 |  |
|  | 27.15 | 2.35 | 5/57 | Juan et al. 2013 |
|  | 86 |  | 31/31 | Lindblad et al. 2013 |
|  | 25 |  | 2/33 |  |
|  | 32 |  | 10/28 |  |
|  | 678 |  | 13/33 |  |
|  | 507 |  | 432/3088 | Marin et al. 2013 |
|  | 107 |  | 35/51 | Pleadin et al. 2013 |
| 13.05 | 13.7 | 12.4 | 2/2 | Aldana et al. 2014 |
|  | 15.3 | 7.4 | 4/17 |  |
| 18 | 19 | 17 | 2/5 | Alkadri et al. 2014 |
| 88 | 231 | 8 | 5/12 |  |
| 15 | 26 | 7 | 4/7 |  |
| 46 |  |  | 1/3 |  |
| 7 |  |  | 1/4 |  |
| 38 | 62 | 11 | 3/12 |  |
| 2.7 |  |  | 5/165 | EFSA CONTAM Panel 2014 |
| 3 |  |  | 2/45 |  |
| 10 |  |  | 1/41 |  |
| 2.6 |  |  | 11/38 |  |
| 1.3 |  |  | 5/59 |  |
| 5.4 |  |  | 27/27 |  |
| 3 |  |  | 44/137 |  |
| 3.6 |  |  | 50/113 |  |
| 3 |  |  | 37/120 |  |
| 64 |  |  | 1/2 | Mortensen and Granby 2014 |
|  | 80 |  | 17/336 | Misca et al. 2014 |
|  | 116 |  | 10/29 | Nordkvist and Häggblom 2014 |
| 39 | 113 |  | 17/? | Kirinčič et al. 2015 |
| 7 | 7 |  | 2/? |  |
|  | 113 |  | 19/80 | Kirinčič et al. 2015 |
| 15.5 | 17 | 14 | 2/4 | Nacher-Mestre et al. 2015 |
| 9 | 15 | 2 | 9/9 | Bryła et al. 2016 |
| 43  16  3.5 | 100  45  4 | 7  1  3 | 18/18 |  |
|  |  |  | 18/18  2/36 |  |
| 45 | 3070 |  | 179/527 | Vogelsang et al. 2017 |
| 18 | 1000 |  | 35/159 |  |

^a^ = reported only means of all samples

| **ZEN in corn** | | | | |
| --- | --- | --- | --- | --- |
| **459** | **3345** |  | **71.1 %** |  |
| mean | max | min | N (pos/all) | References |
| 2350 |  |  | 1/? | Jemmali 1973 |
| 5100 | 10000 | 43 | 3/? | Balzer et al. 1977 |
| 105 |  |  | 1/? | Bartos and Matyas 1981 |
| 12 | 35 | 5 | 6/? | Ranfft et al. 1990 |
| 677 |  |  | 1/? | Tanaka et al. 1990 |
| 30 | 79 | 6 | 17/? | Fazekas et al. 1996 |
|  | 41 | 6.5 | 4/? | Patel et al. 1996 |
| 1080 | 1500 | 640 | 3/? | Scudamore et al. 1998 |
| 46 | 150 | 4 | 14/? | Visconti and Pascale 1998 |
| 228.9 | 1350 | 60 | 140/760 | Rafai et al., 2000 |
| 1.14 | 2.24 | 0.47 | 4/27 | Jaimez et al. 2004 |
|  | 3100 |  |  | Zinedine et al. 2007 |
| 279 | 921 |  | 4/484 | Binder et al. 2007 |
|  | 1403 | 218 | 3/? | Mage et al 2007 |
| 112.0 | 1182 | 27.7 | 21/24 | Segvić Klarić et al. 2009 |
| 27  22 | 178  258 | 20  48 | 4/12  10/66 | Griessler et al. 2010 ^a^  ^a^ |
| 70  480 | 860  14580 |  | 12/44  37/40 | Goertze et al. 2010 |
| 387  147  166.7  127.2 | 356  281 |  | 1/?  1/?  3/?  5/? | Monbaliu et al. 2010 |
|  | 1260 | 16 | 15/19 | Dorn et al. 2011 |
| 122.0  47.2  43.0  34.3 | 603  447  292  422 |  | 26/37  24/25  49/49  66/68 | Grajewski et al. 2012 |
| 41  121  1848 | 1014  7422  16712 |  | 126/394  380/979  28/30 | Rodrigues and Nährer 2012 |
| 246 |  |  | 16/? | EFSA CONTAM Panel 2014 |
| 1225  18 | 4578  31 |  | 8/17.6  4/? | Kirincic et al. 2015 |
|  | 13 | 8 | 3/3 | Nacher-Mestre et al. 2015 |
| 970  2540 | 16200  30130 |  | ?/78  ?/90 | Gromadzka et al. 2016 ^a^ |
| 58.2  31.3  48.5  161.0 | 312  430  405  521 |  | 42/43  78/88  80/91  73/73 | Kosicki et al. 2016 |
|  |  | 2  2 | 1/1  1/1 | Dzuman et al. 2017 Supplementary Data |

^a^ = reported only means of all samples

| **ZEN in barley** | | | | |
| --- | --- | --- | --- | --- |
| **55.8** | **624** |  | **32.1 %** |  |
| mean | max | min | N (pos/all) | References |
|  | 261 | 61 | ? | Bartos and Matyas 1981 |
| 24 | 320 |  | 24/? | Gleissenthal et al. 1989 |
| 7 | 9 | 4 | 6/? | Tanaka et al. 1990 |
| 26 | 30 | 21 | 2/? | Hietaniemi and Kumpulainen 1991 |
| 3  36 | 311 |  | 68/?  68/? | Müller et al. 1997b |
| 18 |  |  | 23/? | Eriksen and Alexander 1998 |
| 139.8 | 840 | 50 | 108/222 | Rafai et al. 2000 |
| 221 | 970 |  | 9/81 | Binder et al 2007 |
| 1.89 | 18.53 |  | 48/123 | Ibáñez-Vea et al. 2012 a |
| 323 |  |  | 18/47 | Rodrigues and Nährer 2012 |
| 11.15 | 15 | 7.3 | 2/9 | Juan et al. 2013 |
| 0.9  3.9  1  3.7 | 21.5  59.4  21.1  47.9 |  | 7/85  18/75  5/85  27/80 | Beláková et al. 2014 |
| 7.2 | 8952 |  | 34/62 | EFSA CONTAM Panel 2014 |
| 75  192  95  2  3 | 214  1558  1116  4  50 |  | ?/12  ?/30  ?/21  ?/35  ?/45 | Nielsen et al 2014 |
| 192.8 | 204.4 | 181.2 | 2/22 | Bolechova et al. 2015 |
| 79 | 203 |  | 5/11 | Kirincic et al. 2015 |
| 7  13 | 19  31 | 2  2 | 10/16  6/8 | Bryła et al. 2016 |
| 3.7  10.2 | 84  240 |  | 59/280  22/160 | Schönenberger et al. 2016 |

| **ZEN in soybean products** | | | | |
| --- | --- | --- | --- | --- |
| **51.6** | **245** |  | **41.7 %** |  |
| mean | max | min | N (pos/all) | References |
| 181.1 | 520 | 50 | 38/119 | Rafai et al 2000 |
|  | 18 |  | 23/51 | Valenta et al. 2002 |
| 50 | 50 |  | 1/18 | Binder et al. 2007 |
| 62.16 | 74.3 |  | 7/7 | Kokic et al. 2009 |
| 13 | 807 |  | 22/156 | Rodrigues and Nährer 2012 |
| 2.7 |  |  | 17/? | EFSA CONTAM Panel 2014 |
| 0.73 | 1.4 | 0.3 | 3/23 | Fapohunda et al. 2018 ^b^ |

^b^ = these data have been obtained from outside of Europe.

| **ZEN in sunflower products** | | | | |
| --- | --- | --- | --- | --- |
|  |  |  | **100 %** |  |
| mean | max | min | N (pos/all) | References |
|  | 83 | 2.5 | ? | Mage et al. 2007 |
| 39.9 | 48.4 |  | 7/7 | Kokic et al. 2009 |
| 13 |  |  | 7/? | EFSA CONTAM Panel 2014 |

| **ZEN in beans** | | | | |
| --- | --- | --- | --- | --- |
| **80.5** | **?** |  | **100** |  |
| mean | max | min | N (pos/all) | References |
| 160 |  |  | 1/? | Pepeljnjak 1984 |
| 1 |  |  | 2/? | EFSA CONTAM Panel 2014 |

| **ZEN in DDGS** | | | | |
| --- | --- | --- | --- | --- |
| **262** | **4653** |  | **59.2 %** |  |
| mean | max | min | N (pos/all) | References |
| 227 | 10374 |  | 85/405 | Rodrigues Chin 2011 ^a^ |
| 910 | 14990 | 100 | 48/59 | Tansakul et al. 2013 ^a^ |
| 65.63 | 2120 | <250 | 27/81 | Khatibi et al. 2014 |
| 78 | 160 | 12 | 4/4 | Mortensen and Granby 2014 |
| 167.6 | 501 | 33 | 52/150 | Abudabos et al. 2017 |

^a^ = reported only means of all samples

| **ZEN in rapeseed products** | | | | |
| --- | --- | --- | --- | --- |
| **13.4** | **27.0** |  | **95.6 %** |  |
| mean | max | min | N (pos/all) | References |
| 12.3  14.3  14.3  19.6  18.6 | 25.1  32.3  25.1  25.6 | 10.6 | 6/7  11/11  25/25  12/13  12/12 | Mankeveciene et al. 2012 |
| 1.3 |  |  | 6/? | EFSA CONTAM Panel 2014 |

| **ZEN in yeast** | | | | |
| --- | --- | --- | --- | --- |
| **31.8** | **?** |  | **100 %** |  |
| mean | max | min | N (pos/all) | References |
| 31.8 |  |  | 1/1 | Kokic et al. 2009 |
| **ZEN in sugarbeetle molasses** | | | | |
| **6.0** | **391** |  | **100 %** |  |
| mean | max | min | N (pos/all) | References |
|  | 391 | 12  0 | 6/?  10/? | Bosch and Mirocha 1992 |
| **ZEN in chicken meat** | | | | |
| **30.2** | **36.4** |  | **58.2 %** |  |
| mean | max | min | N (pos/all) | References |
|  | 103 | 59 | ? | Mirocha et al. 1982 ^b^ |
| 1.45  1.37 | 3.2  2.89 |  | 22/39  27/45 | Iqbal et al. 2014 |

^b^ = these data have been obtained from outside of Europe.


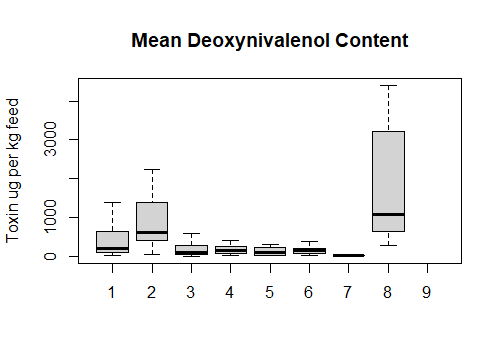


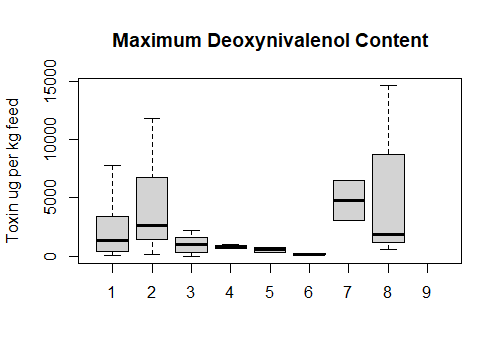


**Figure S3.** Mean (upper graph) and maximum (lower graph) content of DON in feed ingredients (excluding outliers) according to the literature (listed in Annex III). 1 = wheat. 2 = corn, 3 = barley, 4 = soya, 5 = sunflower, 6 = canola, 7 = beans, 8 = DDGS, 9 = meat.

**Table S4** Literature data for deoxynivalenol (DON) in feed ingredients (μg/kg), average values for all reported studies and the percentage of positive tested samples are shaded in light blue.

| **DON in wheat** | | | | |
| --- | --- | --- | --- | --- |
| **894** | **4701** |  | **76.4 %** |  |
| mean | max | min | N (pos/all) | References |
| 3960 | 43800 |  | 87% | Lepschy-von Gleissenthal et al. 1989 |
| 1632 | 20538 |  | 96% | Müller and Schwadorf 1993b |
|  | 8200 |  | ? | Adler et al. 1995 |
|  | 5600 |  | 25/25 | Curtui et al. 1998 |
|  | 77 | 20 | 8/10 | Moura et al. 1998 |
|  |  |  | 24/169 | Langseth and Rundberget 1999 |
| 298.9 | 1560 | 70 | 287/367 | Rafai et al. 2000 |
| 3385  1371  146  70  202 | 6465  3010  330  105  1000 | 1610  380  55  50  60 | 2/2  2/2  5/36  18/35  24/26 | Pascale et al. 2000/2001 |
|  | 1379  965  756 | 15  15  38 | 19/19  28/28  12/13 | Schollenberger et al. 2002 |
|  | 2591 | 10 | 108/120 | Rasmussen et al. 2003 |
|  | 1821 | 333 | 4/10 | Peito and Venâncio 2004 |
|  | 2270 | 20 | 51/103 | Biselli and Hummert 2005 |
| 705 | 5510 |  | 157/254 | Binder et al. 2007 |
|  | 2265.2  804.9  721.9  702 | 17  22.8  18.6  6.8 | 48/48  43/47  44/45  40/41 | Hajšlova et al. 2007 |
|  | 618 | 22 | 13/? | Mage et al. 2007 |
|  | 642  1121  223  445 |  | 61/62  83/88  48/48  32/34 | Mankevičienė et al. 2007 |
|  | 997 |  | 19/32 | Perkowski et al. 2012 |
|  | 455  341 |  | 17/19  12/15 | Perkowski et al. 2007 |
| 1235  182  223  177 | 1840  423  410  208 | 630  57  90  142 | 2/4  4/12  12/34  3/9 | Jajic et al. 2008 |
|  | 200 | 100 | 8/50 | Martins et al. 2008 |
|  | 4130 | 203 | 23/23 | Berthiller et al. 2009 |
|  | 20333 |  | 1396/1624 | Edwards 2009 |
| 600 |  |  | 1/1 | Kokic et al. 2009 |
|  | 3395 | 21 | 38/42 | Macri et al. 2009 |
|  | 434 | 205 | 3/7 | Cunha and Fernandes 2010 |
| 838.29  1163.73 | 4506  8111 | 70  70 | 14/17  12/16 | Giraud et al. 2010 |
| 275 | 2232 | 338 | 8/29 | Griessler et al. 2010 ^a^ |
| 4642  1970  750 | 8841  2113 | 443  1799 | 2/?  3/?  1/? | Monbaliu et al. 2010 |
|  | 154.3 | 6.1 | 10/12 | Stroia et al. 2010 |
|  | 95.7 | 19 | 17/40 | Banu et al. 2011 |
|  | 177 |  | 2/18 | Reinhold and Reinhardt 2011 |
|  | 309 | 41 | 15/54 | Škrbić et al. 2011 |
| 23090  30800 | 69150  52700 | 4010  13400 | 35/35  10/10 | Chelkowski et al. 2012 |
|  | 150 |  | 4/6 | De Boevre et al. 2012 |
| 494.5  1400 | 4557  49000 |  | 188/325  101/191 | Rodrigues and Nährer 2012 |
|  | 976 | 17.5 | 13/15 | Škrbić et al. 2012 |
|  | 3306  1090 | 52  50 | 24/28  70/75 | Stankovic et al. 2012 |
|  | 5865  10000  890  890 |  | 101/338  671/940  245/832  114/554 | Van Der Fels-Klerx et al. 2012 |
|  | 3390  1440 | 294  254 | 19/26  5/26 | Alexa et al. 2013 |
|  | 99.6 | 9.6 | 16/57 | Juan et al. 2013 |
|  | 77 | 20 | 8/10 | Abrunhosa et al. 2016 |
| 68.1  82.2  103.3  41  31  818 | 180  120  551  93  37  1230 | 19  53  62  14  24  13 | 6/12  5/5  5/12  5/7  ¾  3/3 | Alkadri et al. 2014 |
| 104  84  218  161  50  71  118  108  39  16  109  155  62  83  256  209  153  130  405  94  213  161  152  224 |  |  | 136/165  100/107  110/128  83/131  55/142  19/39  7/42  16/38  29/59  83/83  59/59  18/18  74/74  88/88  60/60  93/93  22/22  6/6  30/30  28/31  132/137  106/113  119/120  89/90 | EFSA CONTAM Panel 2014 |
|  | 303  1394  1189  6460 |  | 25/31  21/33  26/28  30/33 | Lindblad et al., 2013 |
|  | 278 |  | 33/51 | Pleadin et al. 2013 |
|  | 83.2 |  | 55/119 | Rodríguez-Carrasco et al. 2013 |
|  | 3230 |  | 26/29 | Nordkvist and Häggblom 2014 |
|  | 7780 |  | 229/290 | Šliková et al. 2014 |
|  | 2940 | 200 | 145/186 | Šliková et al. 2014 |
|  | 7780 | 220 | 20/20 | Šliková et al. 2014 |
| 315 | 330 | 300 | 2/2 | Mortensen and Granby 2014 |
| 473  513 | 3070  2142  3070 |  | 49/?  6/?  55/80 | Kirincic et al. 2015 |
|  | 371  504 | 53  17 | 3/3  4/4 | Nacher-Mestre et al. 2015 |
| 25  590  960  762 | 1616  2975  1721 | 25  82  209  277 | 1/36  18/18  18/18  9/9 | Bryła et al. 2016 |
| 108.2  262.67 | 3602.6  5027.7 | <18.5  <18.5 | ?/816  ?/740 | Gagiu and Smeu 2017 |
| 647  473 | 10600  9880 |  | 442/527  105/159 | Vogelsang et al. 2017 |
| 1063.3 | 1400 | 790 | 3/3 | Yli-Mattila et al. 2017 |

^a^ = reported only means of all samples

| **DON in corn** | | | | |
| --- | --- | --- | --- | --- |
| **3068** | **45380** |  | **73.3 %** |  |
| mean | max | min | N (pos/all) | References |
| 191 | 870 | 50 | 84/780 | Rafai et al. 2000 |
| 1073 | 3970 |  | 197/244 | Binder et al. 2007 |
| 401 | 617 |  | 2/? | Mage et al. 2007 |
| 536  327  426  58 | 2460  2210  1340  172 | 42  40  140  27 | 5/10  32/76  8/21  30/119 | Jajic et al. 2008 |
|  | 500 | 100 | 15/74 | Martins et al. 2008 |
| 460 |  |  | 1/4 | Kokic et al. 2009 |
| 1780  2240 | 19570  16250 |  | 33/44  36/40 | Goertze et al. 2010 |
| 233  547 | 1687  3025 | 68  253 | 7/14  60/71 | Griessler et al. 2010^c^ |
| 2067  268  824  696 | 9528  899  1920  3039 |  | 6/?  7/?  11/?  10/? | Monbaliu et al. 2010 |
|  | 8580 | 210 | 19/19 | Dorn et al. 2011 |
|  | 2248.2 |  | ?/21 | Tabuc et al. 2011 |
|  | 1900 | 300 | 13/? | Cortinovis et al. 2012 |
| 1130  613  653  432 | 6817  3090  4503  4405 |  | 65/66  41/41  53/53  65/68 | Grajewski et al. 2012 |
| 240  634  1705 | 546  26121  11836 |  | 358/459  465/878  26/30 | Rodrigues and Nährer 2012 |
| 2200  1300 | 2900  1900 | 1700  900 | 14/14  14/14 | Zhang and Caupert 2012 |
| 72 |  |  | 16/? | EFSA CONTAM Panel 2014 |
|  | 1523 | 624 | 8/? | Zachariasova et al. 2014 |
| 473  1872 | 3070  11800 |  | 8/?  4/? | Kirincic et al. 2015 |
|  | 814 | 139 | 3/3 | Nacher-Mestre et al. 2015 |
| 6150  90690 | 134360  1505000 |  | ?/78  ?/90 | Gromadzka et al. 2016 ^a^ |
| 624  391  494  1486 | 2027  3075  2473  6688 |  | 42/43  67/88  79/91  73/73 | Kosicki et al. 2016 |
| 548  599.5 | 686  787 | 410  412 | 2/2  2/2 | Dzuman et al. 2017 Supplementary Data |

^a^ = reported only means of all samples

^c^ = DON and ADON levels have been summarized

| **DON in barley** | | | | |
| --- | --- | --- | --- | --- |
| **270** | **2376** |  | **63.2 %** |  |
| mean | max | min | N (pos/all) | References |
| 400 | 4760 |  | 89% | Müller and Schwadorf 1993a |
| 268 | 1200 | 50 | 174/222 | Rafai et al 2000 |
| 805 | 1540 | 70 | 2/2 | Pascale et al. 2000/2001 |
| 394 |  |  | 95/191 | Binder et al. 2007 |
|  | 304 | 40 | 1/? | Jajic et al. 2008 |
|  | 300 | 100 | 3/29 | Martins et al. 2008 |
|  | 997 |  | 1/19 | Griessler et al. 2010 |
|  | 900 | 200 | ? | Cortinovis et al. 2012 |
| 59.6 | 1111 |  | 117/123 | Ibáñez-Vea et al. 2012 b |
| 1677 | 29300 |  | 19/45 | Rodrigues and Nährer 2012 |
|  |  | 35.5 | 1/9 | Juan et al. 2013 |
| 31.1  256  13.5  48.4 | 407.5  2213.5  106.1  985.9 |  | 47/85  73/75  31/85  62/80 | Beláková et al. 2014 |
| 37 |  |  | 805/805 | EFSA CONTAM Panel 2014 |
| 283  211  134  14  56 | 974  3599  707  66  255 |  | ?/12  ?/30  ?/21  ?/35  ?/45 | Nielsen et al. 2014 |
| 284.14 | 602.3 | 69.9 | 19/22 | Bolechova et al. 2015 |
| 289 | 640 |  | 8/11 | Kirincic et al. 2015 |
| 3.4 | 15.1 | 0.2 | 9/50 | Piacentini et al. 2015 |
| 138  602 | 222  1602 | 76  54 | 4/8  16/16 | Bryła et al. 2016 |
| 239.8  46.7 | 4860  1725 |  | 257/280  50/160 | Schönenberger et al. 2016 |

| **DON in beans** | | | | |
| --- | --- | --- | --- | --- |
| **10** | **4800** |  | **100 %** |  |
| mean | max | min | N (pos/all) | References |
|  | 3100  6500 |  |  | Tseng et al. 1995 |
| 10 |  |  | 2/? | EFSA CONTAM Panel 2014 |

| **DON in soybean products** | | | | |
| --- | --- | --- | --- | --- |
| **173** | **703** |  | **32.4 %** |  |
| mean | max | min | N (pos/all) | References |
| 252.7 | 720 | 60 | 78/119 | Rafai et al. 2000 |
| 108 |  |  | 1/51 | Valenta et al. 2002 |
| 397 | 840 |  | 13/32 | Binder et al. 2007 |
| 100 |  |  | 2/? | Jajic et al. 2008 |
| 250 |  |  | 1/7 | Kokic et al. 2009 |
| 202  62 | 908  1019 |  | 10/24  48/159 | Rodrigues and Nährer 2012 |
|  | 30 |  |  | Souza et al. 2013 ^b^ |
| 10 |  |  | 17/? | EFSA CONTAM Panel 2014 |

^b^ = these data have been obtained from outside of Europe.

| **DON in sunflower products** | | | | |
| --- | --- | --- | --- | --- |
| **106** | **559** |  | **30.8** |  |
| mean | max | min | N (pos/all) | References |
| 150 |  |  | 1/22 | Rafai et al. 2000 |
| 25 |  |  | 1/? | Mage et al. 2007 |
|  | 788 | 40 | 9/? | Jajic et al. 2008 |
| 302.5 | 330 |  | 4/7 | Kokic et al. 2009 |
| 12 |  |  | 7/? | EFSA CONTAM Panel 2014 |

| **DON in rapeseed products** | | | | |
| --- | --- | --- | --- | --- |
| **165** | **188** |  | **72.9 %** |  |
| mean | max | min | N (pos/all) | References |
| 387  209.7  133  165 | 226  278.3  181  176.5 | 153.5 | 7/7  2/11  21/25  6/8  8/8 | Mankeveciene et al. 2012 |
| 19 |  |  | 6/? | EFSA CONTAM Panel 2014 |
| 79 | 79 | 68 | 3/5 | Mortensen and Granby 2014 |

| **DON in DDGS** | | | | |
| --- | --- | --- | --- | --- |
| **1919** | **6017** |  | **79.9 %** |  |
| mean | max | min | N (pos/all) | References |
| 461.04  1018.13  684.24  767 | 1900  1680  1420  1200 | 200  130  120  100 | 77/77  16/16  66/69  9/20 | Zhang et al. 2009 ^b^    ^b^ |
| 1755 | 24269 |  | 77/409 | Rodrigues-Chin 2011 ^a^ |
| 7900  4400  3408.47 | 9300  5600  12300 | 6200  3200  300 | 14/14  14/14  59/59 | Zhang and Caupert 2012 ^b^ |
| 1160 | 6750 | 260 | 29/59 | Tansakul et al. 2013 ^a^ |
| 1915.29 | 14620 | <500 | 87/141 | Khatibi et al. 2014 |
| 580  290 | 1100  570 | 140  90 | 4/4  7/7 | Mortensen and Granby 2014 |
| 3000 | 8100 | 800 | 43/150 | Abudabos et al. 2017 |
| 579.5 | 724 | 435 | 2/2 | Dzuman et al. Supplementary Data |
| 869 | 724 | 435 | 2/2 | Duzman et al. 2017 |

^a^ = reported only means of all samples

^b^ = these data have been obtained from outside of Europe.

**
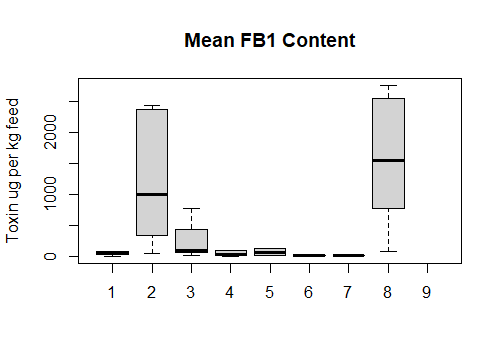
**


**
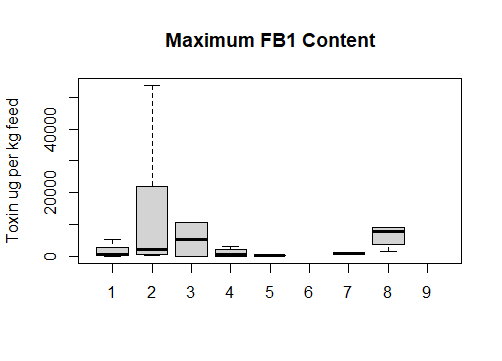
**

**Figure S4.** Mean (upper graph) and maximum (lower graph) content of fumonisin B_1_ in feed ingredients (excluding outliers) according to the literature (listed in Annex III). 1 = wheat. 2 = corn, 3 = barley, 4 = soya, 5 = sunflower, 6 = canola, 7 = beans, 8 = DDGS, 9 = meat.

**Table S5** Literature data for fumonisin B_1_ (FB_1_) in feed ingredients (μg/kg), average values for all reported studies and the percentage of positive tested samples are shaded in light blue.

| **FB_1_ in wheat** | | | | |
| --- | --- | --- | --- | --- |
| **202** | **1565** |  | **42.7 %** |  |
| mean | max | min | N (pos/all) | References |
|  | 736  4343 |  | 87/110  42/256 | SCOOP 2003 |
| 580 | 580 |  | 1/1 | Binder et al. 2007 |
|  | 40 | 10 | 4/50 | Martins et al. 2008 |
| 78 |  |  | 1/? | Monbaliu-et-al-2010 |
|  |  |  | 12/21 | Tabuc et al. 2011 |
|  | 5400  4900 | 750  750 | 23/28  69/75 | Stankovic et al. 2012 |
| 28 | 874 |  | 16/175 | Rodrigues and Nährer 2012 |
|  | 59.8  125.8 | 20.2  75.7 | 6/44  9/63 | Rubert et al. 2013 |
| 15 |  |  | 6/? | EFSA CONTAM Panel 2014 |
| 1159.7 | 2319 | 0.4 | 3/3 | Nacher-Mestre et al. 2015 |
| 50  68 | 150 | 50  40 | 1/9  4/18 | Bryła et al. 2016 |

| **FB_1_ in corn** | | | | |
| --- | --- | --- | --- | --- |
| **3221** | **28816** |  | **62.6 %** |  |
| mean | max | min | N (pos/all) | References |
| 100 | 800 |  | 44/120 | Shephard et al 1996 |
| 11900 | 32200 | 25 | 8/12 | Peito and Venancio 2004 |
| 460 | 1162 | 113 | 8/11 | Lino et al. 2006b |
| 836 |  |  | 9/16 | Binder et al. 2007 |
|  | 300 | 10 | 12/58 | Martins et al. 2008 |
| 443.7 | 543 |  | 3/4 | Kokic et al. 2009 |
| 7630 | 20700 |  | 3/12 | Klarić et al. 2009 ^d^ |
| 1910 | 20690 |  | 15/44 | Goertze et al. 2010 |
| 2195  6306 | 7714  36390 | 92  584 | 20/21  18/29 | Griessler et al. 2010 ^a^ |
| 370.6  200.8  1425.6  1121.4 | 853  511  5114  3761 |  | 5/?  6/?  12/?  11/? | Monbaliu et al. 2010 |
|  | 2110 | 1180 | 2/19 | Dorn et al. 2011 |
|  | 100 | 0.1 | 61/95 | Soares and Venancio 2011 |
|  |  |  | 10/21 | Tabuc et al. 2011 |
| 11300.0  959.0  78.8  51.0 | 9409  6163  701  435 |  | 41/45  10/10  15/18  22/42 | Grajewski et al. 2012 ^d^ |
| 813  2431  5871 | 22900  53700  32510 |  | 70/157  921/1110  29/29 | Rodrigues and Nährer 2012 |
| 992  1662 |  |  | 49/55  13/? | EFSA CONTAM Panel 2014 |
|  | 189 |  | 8/? | Zachariasova et al. 2014 |
| 6404  238 | 27483  304 |  | 5/?  3/? | Kirincic et al. 2015 ^e^ |
| 1159.7 | 2319 | 0.4 | 3/3 | Nacher-Mestre et al. 2015 |
| 44460  2310 | 525920  103210 |  | ?/78  ?/90 | Gromadzka et al. 2016 ^a^ |
| 550.0  67.1  83.3  307.0 | 1885  219  264  1559 |  | 10/17  6/25  16/24  16/17 | Kosicki et al. 2016 |
|  |  | 43  45 | 1/1  1/1 | Dzuman et al. 2017 Supplementary Data |

^a^ = reported only means of all samples

^d^ = summarized values for FB1, FB2 and FB3

^e^ = summarized values for FB1, FB2 and FB3

| **FB_1_ in barley** | | | | |
| --- | --- | --- | --- | --- |
| **222** | **5243** |  | **13.8 %** |  |
| mean | max | min | N (pos/all) | References |
| 10 |  |  | 2/29 | Martins et al. 2008 |
| 777 | 10485 |  | 6/23 | Rodrigues and Nährer 2012 |
|  | 0.013 | 0.001 | 5/50 | Piacentini et al. 2015 |
| 101 |  |  | 1/8 | Bryła et al. 2016 |

| **FB_1_ in beans** | | | | |
| --- | --- | --- | --- | --- |
| **5** | **800** |  | **100%** |  |
| mean | max | min | N (pos/all) | References |
|  | 500  1100 |  |  | Tseng et al. 1995 |
| 5 |  |  | 2/? | EFSA CONTAM Panel 2014 |
| **FB_1_ in rapeseed products** | | | | |
| **6.7** | **?** |  | **100 %** |  |
| mean | max | min | N (pos/all) | References |
| 6.7 |  |  | 6/? | EFSA CONTAM Panel 2014 |
| **FB_1_ in sunflower products** | | | | |
| **63.9** | **168** |  | **28.6 %** |  |
| mean | max | min | N (pos/all) | References |
| 117.8 | 168 |  | 2/7 | Kokic et al. 2009 |
| 10 |  |  | 2/? | EFSA CONTAM Panel 2014 |

| **FB_1_ in soybean products** | | | | |
| --- | --- | --- | --- | --- |
| **546** | **1060** |  | **25.8 %** |  |
| mean | max | min | N (pos/all) | References |
| 3120 | 3120 |  | 1/2 | Binder et al. 2007 |
| 97.4 | 97.4 |  | 1/7 | Kokic et al. 2009 |
|  | 1008.1 |  | ? | Tabuc et al. 2011 |
| 27 |  |  | 9/157 | Rodrigues and Nährer 2012 |
| 22 |  |  | 30/? | EFSA CONTAM Panel 2014 |
| 0.4 |  |  | 2/4 | Nacher-Mestre et al. 2015 |
| 97.4 | 97.4 |  | 1/7 | Fapohunda et al. 2018 ^b^ |

^b^ = these data have been obtained from outside of Europe.

| **FB_1_ in DDGS** | | | | |
| --- | --- | --- | --- | --- |
| **2401** | **22949** |  | **80.4 %** |  |
| mean | max | min | N (pos/all) | References |
| 2758.67  1471.25  2327.24  85.22 | 7200  2770  5880  8600 | 800  280  120  100 | 75/77  69/69  69/69  13/20 | Zhang et al. 2009 ^b,e^ |
| 892 | 9042 |  | 91/390 | Rodrigues-Chin-2011 ^a^ |
| 2162.36 | 8900 | 100 | 44/59 | Zhang and Caupert 2012 ^b,d^ |
| 7590  9080 | 38860  143000 | 840  320 | ?/30  58/59 | Tansakul et al. 2013 ^a^ |
| 650 | 1600 | 33 | 19/19 | Mortensen and Granby 2014 |
| 1640 | 3640 | 430 | 38/150 | Abudabos et al. 2017 |
| 80 |  |  | 1/1 | Dzuman et al. 2017 Supplementary Data |
|  |  | 80 | 1/1 | Duzman et al. 2017 |

^a^ = reported only means of all samples

^b^ = these data have been obtained from outside of Europe.

^d^ = summarized values for FB_1_, FB_2_ and FB_3_

^e^ = summarized values for FB_1_ and FB_2_


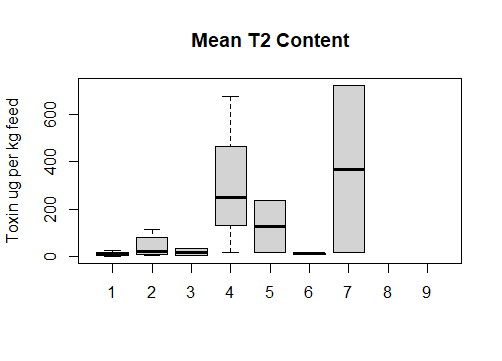


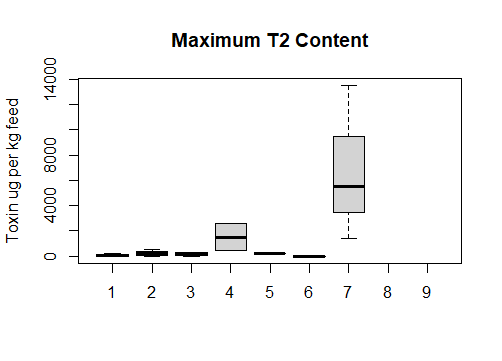


**Figure S5.** Mean (upper graph) and maximum (lower graph) content of T-2 toxin in feed ingredients (excluding outliers) according to the literature (listed in Annex III). 1 = wheat. 2 = corn, 3 = barley, 4 = soya, 5 = sunflower, 6 = canola, 7 = beans, 8 = DDGS, 9 = meat.

**Table S6** Literature data for T-2 toxin in feed ingredients (μg/kg), average values for all reported studies and the percentage of positive tested samples are shaded in light blue.

| **T-2 in wheat** | | | | |
| --- | --- | --- | --- | --- |
| **22.9** | **124** |  | **41.2 %** |  |
| mean | max | min | N (pos/all) | References |
|  | 600 | 5 | 38.0% | Lepschy-von Gleissenthal et al. 1989 |
| 82 | 249 |  | 26.0 % | Müller and Schwadorf 1993b |
| 55 |  |  | 1/140 | Vrabcheva et al. 1996 |
|  | 63 |  | 6/25 | Curtui et al. 1998 |
|  |  | 20 | 1/169 | Langseth and Rundberget 1999 |
| 193.8 | 370 | 80 | 25/367 | Rafai et al. 2000 |
|  |  | 4 | 1/19 | Schollenberger et al. 2002 |
|  | 153 |  | 11/38 | Rasmussen et al. 2003 |
| 187 | 829 |  | 18/18 | Binder et al. 2007 |
|  | 8.2 | 5.7 | 16/41 | Hajšlova et al. 2007 |
|  | 1.9 |  | 110/130 | Gottschalk et al. 2009 |
| 26 |  |  | 1/? | Monbaliu et al. 2010 |
| 0.9 | 1 | 0.8 | 2/2 | Galbenu-Morvay et al. 2011 |
|  | 18.8  23 |  | 33/48  34/34 | Mankevičienė et al. 2011 |
|  | 4 |  | 3/19 | Perkowski et al. 2012 |
|  | 26.9 | 9.8 | 4/15 | Škrbić et al. 2012 |
| 12.5 | 17.8 | 7.17 | 2/57 | Juan et al. 2013 |
|  | 18 |  | 13/51 | Pleadin et al. 2013 |
| 9  12  11  2 | 51 | 2 | 1/12  7/12  1/7  1/4 | Alkadri et al. 2014 |
| 15  19  15  14  13  5.3  15  15  15  15  14  13  15  15  3.1  8.5  15  19 |  |  | 1/42  3/38  1/59  83/83  59/59  18/18  74/74  1/88  1/60  1/93  22/22  6/6  1/30  27/27  1/137  12/113  14/120  1/90 | EFSA CONTAM Panel 2014 |
| 4  4 |  |  | 1/3  2/4 | Nacher-Mestre et al. 2015 |
| 4  2  3 | 5  6  22 | 3  1  1 | 7/9  18/18  16/18 | Bryła et al. 2016 |

| **T-2 in corn** | | | | |
| --- | --- | --- | --- | --- |
| **28** | **265** |  | **52.3 %** |  |
| mean | max | min | N (pos/all) | References |
| 225.2 | 980 | 50 | 220/780 | Rafai et al. 2000 |
| 188 | 188 |  | 1/18 | Binder et al. 2007 |
| 4  10 | 30  340 |  | 6/44  4/49 | Goertze et al. 2010 |
| 13.5  112  10 | 18 |  | 4/?  1/?  1/? | Monbaliu et al. 2010 |
| 45.7  36.5  191 | 289  174  11 |  | 10/10  22/25  44/68 | Grajewski et al. 2012 |
| 15 |  |  | 16/? | EFSA CONTAM Panel 2014 |
| 2.8 |  |  | 1/3 | Nacher-Mestre et al. 2015 |
| 45  7.81  10.3  29.3 | 434  63.7  100  550 |  | 34/43  30/88  67/91  71/73 | Kosicki et al. 2016 |

| **T-2 in barley** | | | | |
| --- | --- | --- | --- | --- |
| **91.8** | **178** |  | **36.6 %** |  |
| mean | max | min | N (pos/all) | References |
| 220 | 310 | 50 | 16/222 | Rafai et al. 2000 |
| 921 | 921 |  | 1/5 | Binder et al. 2007 |
| 34.9 | 332 |  | 12/123 | Ibáñez-Vea et al. 2012 b |
| 15 |  |  | 785/785 | EFSA CONTAM Panel 2014 |
| 5  6  12  9  5 | 5  24  130  52  22 |  | ?/12  ?/30  ?/21  ?/35  ?/45 | Nielsen et al. 2014 |
| 24 | 30.5 | 14.85 | 8/22 | Bolechova et al. 2015 |
| 5  3 | 11  5 | 2  1 | 8/8  7/16 | Bryła et al. 2016 |
| 15.4  9.7 | 319  155 |  | 11/180  10/160 | Schönenberger et al. 2016 |

| **T-2 in soybean products** | | | | |
| --- | --- | --- | --- | --- |
| **93.0** | **450** |  | **100 %** |  |
| mean | max | min | N (pos/all) | References |
| 249.1 | 450 | 50 | 22/119 | Rafai et al. 2000 |
| 15 |  |  | 7/? | EFSA CONTAM Panel 2014 |
| **T-2 in beans** | | | | |
| **15** | **9500** |  | **100 %** |  |
| mean | max | min | N (pos/all) | References |
|  | 13500  5500 |  | ?  ? | Tseng et al. 1995 |
| 15 |  |  | 2/? | EFSA CONTAM Panel 2014 |
| **T-2 in rapeseed products** | | | | |
| **11.4** | **11.5** |  | **93.3 %** |  |
| mean | max | min | N (pos/all) | References |
| 11.8  9.7  9.2 | 10.1  11.6  10.1  10.2 | 8.5 | 2/3  9/9  19/19  8/8  8/8 | Mankeveciene et al. 2012 |
| 15 |  |  | 6/? | EFSA CONTAM Panel 2014 |
| **T-2 in sunflower products** | | | | |
| **126** | **250** |  | **13.6 %** |  |
| mean | max | min | N (pos/all) | References |
| 236.7 | 250 | 230 | 3/22 | Rafai et al. 2000 |
| 15 |  |  | 7/? | EFSA CONTAM Panel 2014 |

**
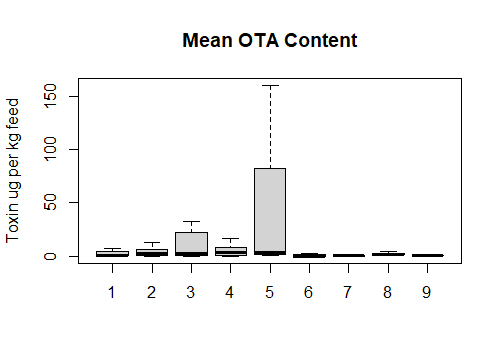
**

**
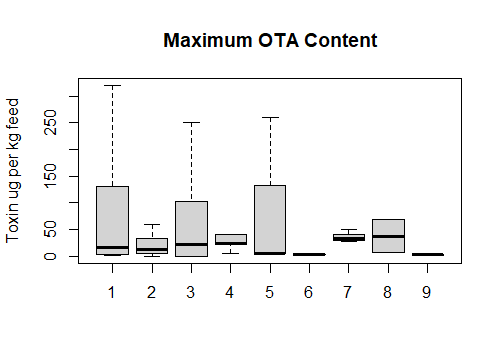
**

**Figure S6.** Mean (upper graph) and maximum (lower graph) content of ochratoxin (OTA) in feed ingredients (excluding outliers) according to the literature (listed in Annex III). 1 = wheat. 2 = corn, 3 = barley, 4 = soya, 5 = sunflower, 6 = canola, 7 = beans, 8 = DDGS, 9 = meat.

**Table S7** Literature data for ochratoxin A (OTA) in feed ingredients (μg/kg), average values for all reported studies and the percentage of positive tested samples are shaded in light blue.

| **OTA in wheat** | | | | |
| --- | --- | --- | --- | --- |
| **18.8** | **125** |  | **30.8 %** |  |
| mean | max | min | n | References |
|  | 17 |  | 10/50 | Scudamore et al. 1997 |
|  | 231 | 0.3 | 32/201 | Prickett et al. 2000 |
| 207.8  120 | 320  320 | 90 | 9/367  1/32 | Rafai et al. 2000 |
| 0.83 | 1.2 | 0.48 | 3/31 | Czerwiecki et al. 2002a |
| 267  1.17 | 1024  1.6 | 0.6  0.8 | 18/37  8/34 | Czerwiecki et al. 2002b |
| 0.3  0.5  0.3  0.3 | 16  19  32  1.6 |  | 108/156  101/120  217/405  6/14 | Jørgensen and Jacobsen 2002 |
| 0.19  0.25 |  |  | 2/34  2/8 | Miraglia and Brera 2002 |
| 1.4 | 1.47 |  | 6/70 | Palermo et al. 2002 |
| 4.6 | 12.1 |  | 10/14 | Beg et al. 2006 |
| 4 | 7 |  | 5/12 | Binder et al. 2007 |
|  | 3 | 2.3 | ? | Mage et al. 2007 |
| 12.7 | 31.7 |  | 3/12 | Klaric´ et al. 2009 |
| 4.79 |  |  | 1/1 | Kokic et al. 2009 |
| 0.3 |  | 3 | 1/4 | Griessler et al. 2010 |
| 1  5.7 | 331 |  | 26/124  4/20 | Rodrigues and Nährer 2012 |
| 0.2  0.7  0.4  0.3  0.4  0.2  0.5  0.3 |  |  | 5/165  8/18  5/33  6/45  2/42  16/38  3/59  28/? | EFSA CONTAM Panel 2014 |
| 2.1 |  |  | 1/2 | Mortensen and Granby 2014 |
| 3.9 | 5.8 |  | 2/80 | Kirincic et al. 2015 |
| 0.4 | 5.2 | 2 | 1/3  4/4 | Nacher-Mestre et al. 2015 |
| 7  4 |  | 7  4 | 1/9  1/18 | Bryła et al. 2016 |

| **OTA in corn** | | | | |
| --- | --- | --- | --- | --- |
| **19.6** | **122** |  | **35.0 %** |  |
| mean | max | min | n | References |
|  | 35  25 | 25  10 | ?  ? | Petkova-Bocharova and Castegnaro 1985 |
| 320 | 1850 | 60 | 70/760 | Rafai et al 2000 |
| 1.7 | 5.2 |  | 19/70 | Palermo et al. 2002 |
| 1.95  3.95 | 3.2  12.2 |  | 2/13  9/27 | Jaimez et al. 2004 |
| 1.47 | 2.54 |  | 19/49 | Domijan et al. 2005 |
| 6.38 | 14.5 |  | 31/32 | Beg et al. 2006 |
| 0 |  |  | 0/11 | Binder et al. 2007 |
|  | 6 | 5.8 | 3/? | Mage et al. 2007 |
| 12.7 | 31.7 | 2.5 | 3/12 | Klarić et al. 2009 |
| 1 |  |  | 2/7 | Griessler et al. 2010 |
| 22 |  |  | 1/? | Monbaliu et al. 2010 |
| 0.25  0.21  0.51  4.33 | 0.78  0.23  1.15  13.6 |  | 28/37  7/19  5/30  5/39 | Grajewski et al. 2012 |
| 1  3  6 | 11  355  59.7 |  | 1/12  61/557  20/30 | Rodrigues and Nährer 2012 |
| 1.3 |  |  | 16/? | EFSA CONTAM Panel 2014 |
| 0.4 |  |  | 3/3 | Nacher-Mestre et al. 2015 |
| 20.1  32.2  2.39  5.78 | 32.5  86  5.09  11.9 |  | 2/30  3/19  3/32  4/32 | Kosicki et al. 2016 |

| **OTA in barley** | | | | |
| --- | --- | --- | --- | --- |
| **13.7** | **58.1** |  | **16.9 %** |  |
| mean | max | min | n | References |
|  | 102 |  | 12/45 | Scudamore et al. 1997 |
|  | 117 | 0.3 | 20/106 | Prickett et al. 2000 |
| 76.7 | 250 | 50 | 6/222 | Rafai et al. 2000 |
| 25.73 | 57 | 0.3  6.7 | 1/26  3/40 | Czerwiecki et al. 2002a |
| 5.45  18.4 | 9.7  35.3 | 1.2  1.43 | 2/36  2/17 | Czerwiecki et al. 2002b |
| 33 |  |  | 1/31 | Binder et al. 2007 |
| 2.6 |  |  | 1/6 | Klarić et al. 2009 |
| 0.1 | 3.53 |  | 71/123 | Ibáñez-Vea et al. 2012 a |
| 1 | 9.6 |  | 1/15 | Rodrigues and Nährer 2012 |
| 1.01 |  |  | 27/82 | EFSA CONTAM Panel 2014 |

| **OTA in soybean products** | | | | |
| --- | --- | --- | --- | --- |
| 42.9 | 77.1 |  | 35.3 % |  |
| mean | max | min | n | References |
| 350 | 350 | 50 | 2/119 | Rafai et al. 2000 |
| 1 |  |  | 5/51 | Valenta et al. 2002 |
| 7.9 | 40 |  | 18/21 | Beg et al. 2006 |
| 0.02 |  |  | 2/? | Mage et al. 2007 |
| 3.97 | 5.12 |  | 6/7 | Kokic et al. 2009 |
| 5  1 | 21.4  23 |  | 1/4  24/122 | Rodrigues and Nährer 2012 |
| 0.06 |  |  | 21/? | EFSA CONTAM Panel 2014 |
| 16.8 | 23.1 | 10.5 | 2/23 | Fapohunda et al. 2018 ^b^ |

^b^ = these data have been obtained from outside of Europe.

| **OTA in fishmeal** | | | | |
| --- | --- | --- | --- | --- |
| **2.31** | **3.48** |  | **20.0 %** |  |
| mean | max | min | n | References |
|  | 0.13 | 0.06 | 4/? | Mage et al. 2007 |
| 6.83 | 6.83 |  | 1/5 | Kokic et al. 2009 |
| 0.05 |  |  | 49/? | EFSA CONTAM Panel 2014 |
| **OTA in beans** | | | | |
| **20.3** | **36.7** |  | **36.7 %** |  |
| mean | max | min | n | References |
|  | 27  50 | 25  25 | 16.7%  7.1% | Petkova-Bocharova and Castegnaro 1985 |
|  | 33 |  | 1/15 | Scudamore et al. 1997 |
| 0.41 |  |  | 17/45 | Domijan et al. 2005 |
| 0.03 |  |  | 2/? | EFSA CONTAM Panel 2014 |
| 1.8 |  |  | 1/3 | Nacher-Mestre et al. 2015 |
| **OTA in rapeseed products** | | | | |
| **0.77** | **2.6** |  | **100 %** |  |
| mean | max | min | n | References |
| 0.02 |  |  | 1/? | Mage et al. 2007 |
| 0.04 |  |  | 6/? | EFSA CONTAM Panel 2014 |
| 2.6 | 2.6 |  | 1/1 | Mortensen and Granby 2014 |
| 0.4 |  |  | 1/1 | Nacher-Mestre et al. 2015 |
| **OTA in sunflower products** | | | | |
| **33.9** | **89.9** |  | **63.2 %** |  |
| mean | max | min | N | References |
| 160 | 260 | 100 | 4/22 | Rafai et al 2000 |
|  | 5.8 | 2.4 | 5/? | Mage et al. 2007 |
| 2.73 | 3.82 |  | 5/7 | Kokic et al. 2009 |
| 3.9 |  |  | 7/? | EFSA CONTAM Panel 2014 |
| 0.4 |  |  | 1/1 | Nacher-Mestre et al. 2015 |
| **OTA in yeast** | | | | |
| **2.26** | **2.26** |  | **100 %** |  |
| mean | Max | min | n | References |
| 2.26 | 2.26 |  | 1/1 | Kokic et al. 2009 |
| **OTA in DDGS** | | | | |
| **2.57** | **37.7** |  | **45.0 %** |  |
| mean | max | min | n | References |
| 2 | 68 |  | 25/173 | Rodrigues-Chin 2011 |
| 4 |  |  | 433/1238 | Schatzmayr and Streit 2013 |
| 1.7 | 7.3 | 0.5 | 6/7 | Mortensen and Granby 2014 |
| **OTA in chicken meat** | | | | |
| **0.81** | **2.49** |  | **45.0 %** |  |
| mean | max | min | n | References |
| 0.28 | 3.67 |  | 16/39 | Rodrigues-Chin 2011 |
| 0.81 | 1.3 |  | 22/45 | Mortensen and Granby 2014 |


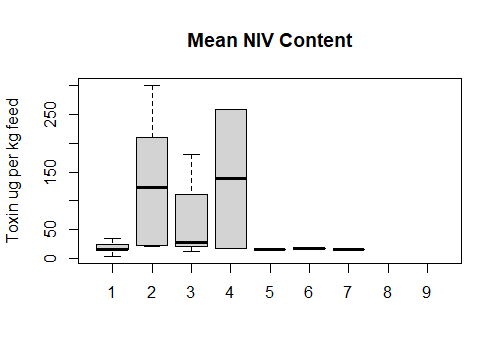


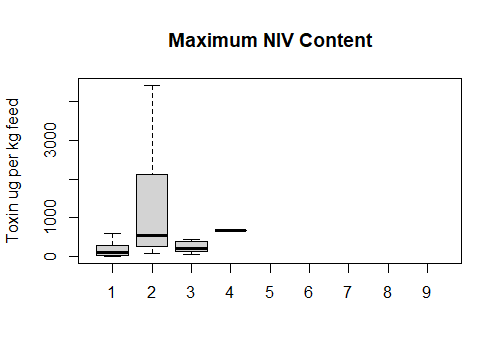


**Figure S7.** Mean (upper graph) and maximum (lower graph) content of nivalenol (NIV) in feed ingredients (excluding outliers) according to the literature (listed in Annex III). 1 = wheat. 2 = corn, 3 = barley, 4 = soya, 5 = sunflower, 6 = canola, 7 = beans, 8 = DDGS, 9 = meat.

**Table S8** Literature data for nivalenol (NIV) in feed ingredients (μg/kg), average values for all reported studies and the percentage of positive tested samples are shaded in light blue.

| **NIV in wheat** | | | | |
| --- | --- | --- | --- | --- |
| **28.9** | **177** |  | **33.1 %** |  |
| mean | max | min | n | References |
|  |  |  | 30% | Lepschy-von Gleissenthal et al. 1989 |
|  | 32 | 9 | 26% | Müller and Schwadorf 1993b |
| 177.3 | 590 | 50 | 33/367 | Rafai et al. 2000 |
|  | 25 | 25  25 | 1/28  1/13  5/19 | Schollenberger et al. 2002 |
|  | 234 | 10 | 57/120 | Rasmussen et al. 2003 |
|  | 440 |  | ? | SCOOP 2003 |
|  | 25.9 | 15.4 | 32/41 | Hajšlova et al. 2007 |
|  | 80 |  | 14/32 | Perkowski et al. 2007 |
|  | 430 |  | 1088/1624 | Edwards 2009 |
|  |  | 7 | 1/42 | Macri et al. 2009 |
|  | 13 |  | 3/57 | Scudamore et al. 2009 |
| 256.7 | 293 | 236 | 3/? | Giraud et al. 2010 |
| 86 |  |  | 1/? | Monbaliu et al. 2010 |
|  | 23  18 |  | 8/15  16/19 | Perkowski et al. 2012 |
|  | 26.9 | 9.8 | 5/14 | Škrbić et al. 2012 |
|  | 495  200 | 60  86 | 21/28  45/75 | Stankovic et al. 2012 |
|  | 106  106 | 12  12 | 16/57  11/57 | Juan et al. 2013 |
|  | 111  39  39  50 |  | 29/31  11/33  14/28  9/33 | Lindblad et al. 2013 |
|  | 53.6 |  | 16/119 | Rodríguez-Carrasco et al. 2013 |
| 183 | 290 | 67 | 3/12 | Alkadri et al. 2014 |
| 27  35  25  25  26  25  10  21  11  12  11  5.3  15  15  15  15  15  13  15  16  3.9  15  16  19 | 290 | 40 | 1/165  27/107  1/128  1/131  4/142  1/39  1/42  7/38  8/59  83/83  59/59  18/18  74/74  1/88  1/60  1/93  22/22  6/6  1/30  28/28  11/137  2/113  10/111  1/72 | EFSA CONTAM Panel 2014 |
|  | 12 |  | 2/29 | Nordkvist and Häggblom 2014 |
| 15  16 | 470  290 |  | 100/527  40/159 | Vogelsang et al. 2017 |

| **NIV in corn** | | | | |
| --- | --- | --- | --- | --- |
| **1551** | **29540** |  | **53.2 %** |  |
| mean | max | min | n | References |
| 186.7 | 260 | 130 | 3/780 | Rafai et al. 2000 |
|  | 340 |  | ? | SCOOP 2003 |
| 2547  94 |  |  | 1/?  1/? | Monbaliu et al. 2010 |
|  | 1300 | 80 | 7/19 | Dorn et al. 2011 |
| 160  210 | 4410  2120 |  | 10/44  19/40 | Goertze et al. 2010 |
| 37.3 | 76.8 | < 15 | 2/2  30/68 | Grajewski et al. 2012 |
| 21 |  |  | 16/? | EFSA CONTAM Panel 2014 |
| 153 | 580 |  | 8/? | Zachariasova et al. 2014 |
| 300  17970 | 5720  368200 |  | 78  90 | Gromadzka et al. 2016 ^a^ |
| 20.7  22.1  23.2  39 | 89.2  251  122  550 |  | 34/43  45/88  80/91  73/73 | Kosicki et al. 2016 |

^a^ = reported only means of all samples

| **NIV in barley** | | | | |
| --- | --- | --- | --- | --- |
| **57.5** | **296** |  | **34.8 %** |  |
| mean | max | min | n | References |
| 130 |  |  | 1/2 | Hysek et al. 1999 |
| 136.4 | 340 | 60 | 14/222 | Rafai et al 2000 |
| 20.8 | 142.5 |  | 25/123 | Ibáñez-Vea et al. 2012 b |
|  | 106 | 21.7 | 3/9 | Juan et al. 2013 |
| 16 |  |  | 805/805 | EFSA CONTAM Panel 2014 |
| 24  22  34  26  180 | 47  206  122  209  1089 |  | ?/12  ?/30  ?/21  ?/35  ?/45 | Nielsen et al. 2014 |
| 93.4 | 123.3 | 52 | 8/22 | Bolechova et al. 2015 |
| 12.5  31 | 435  431 |  | 73/280  10/160 | Schönenberger et al. 2016 |

| **NIV in soybean products** | | | | |
| --- | --- | --- | --- | --- |
| **138** | **680** |  | **100 %** |  |
| mean | max | min | n | References |
| 258.7 | 680 | 50 | 22/119 | Rafai et al. 2000 |
| 18 |  |  | 7/? | EFSA CONTAM Panel 2014 |
| **NIV in beans** | | | | |
| **15** | **?** |  | **100 %** |  |
| mean | max | min | N | Reference |
| 15 |  |  | 2/? | EFSA CONTAM Panel 2014 |
| **NIV in rapeseed prducts** | | | | |
| **18** | **?** |  | **100 %** |  |
| mean | max | min | N | Reference |
| 18 |  |  | 6/? | EFSA CONTAM Panel 2014 |
| **NIV in sunflower products** | | | | |
| **15** | **?** |  | **100 %** |  |
| mean | max | min | n | Reference |
| 15 |  |  | 7/? | EFSA CONTAM Panel 2014 |


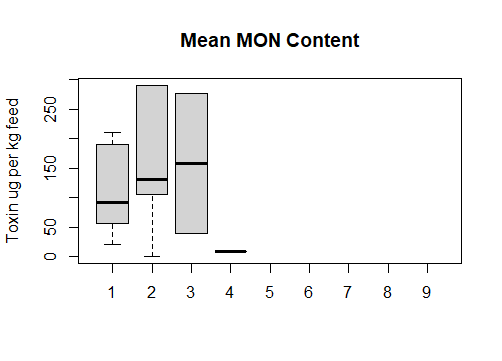

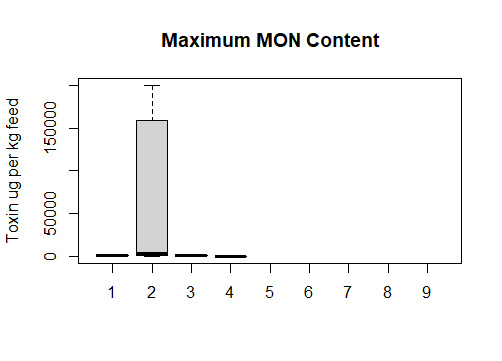


**Figure S8.** Mean (upper graph) and maximum (lower graph) content of moniliformin (MON) in feed ingredients (excluding outliers) according to the literature (listed in Annex III). 1 = wheat. 2 = corn, 3 = barley, 4 = soya, 5 = sunflower, 6 = canola, 7 = beans, 8 = DDGS, 9 = meat.

**Table S9** Literature data for moniliformin (MON) in feed ingredients (μg/kg), average values for all reported studies and the percentage of positive tested samples are shaded in light blue.

| **MON in wheat** | | | | |
| --- | --- | --- | --- | --- |
| **657** | **2985** |  | **86.4 %** |  |
| mean | max | min | n | References |
| 8670 | 17100 | 500 | 6/6 | Sharman et al. 1991 |
|  | 880 |  | ? | Adler et al. 1995 |
|  | 200 |  | 10/? | Krysinska-Traczyk et al. 2001 |
| 171.9 | 810 | 30 | 10/14 | Jestoi et al. 2004a |
|  | 950 | 130 | 83/? | Uhlig et al. 2004 |
|  | 950 |  | ? | Sørensen et al. 2007 |
| 98  71  52  211  59  47 |  |  | 25/?  30/?  27/?  25/?  30/?  27/? | Bernhoft et al. 2008 |
|  | 6.5 | 7  3.1 | 1/3  2/2 | vanBargen et al. 2012 |
| 20  92  210 |  |  | 13/13  35/35  35/35 | EFSA CONTAM Panel 2014 |

| **MON in corn** | | | | |
| --- | --- | --- | --- | --- |
| **9318** | **111412** |  | **65.7 %** |  |
| mean | max | min | n | References |
|  |  |  | 23/58 | Thalmann et al. 1985 |
| 130 | 200 | 60 | 2/12 | Scott and Lawrence 1987 |
| 0.73  97715 | 399300 | 4200 | 1/10  20/20 | Sharman et al.1991 |
|  | 425000 | 17000 | ? | Logrieco et al 1993 |
|  | 8530 | 450 | 6/12 | Kostecki et al. 1995 |
|  | 200000 |  | 8/14 | Logrieco et al 1995 |
|  | 2000 | 50 | ? | Filek and Lindner, 1996 |
|  | 530000  20000 | 4200 | 87/87  21/25 | Lew et al. 1991,1996 |
|  | 530000  530000 | 66000  30000 | ?  ? | Chelkowski et al. 1998 |
|  | 1340 |  | ? | Noser et al. 2001 |
|  | 4600 |  | 60% | Scudamore et al. 1998 |
| 280  110 | 3330  1850 |  | 19/44  18/40 | Goertze et al. 2010 |
|  | 1030 | 160 | ? | Parich et al. 2003 |
| 1127  106  262  89 | 2606  527  920  409 |  | 16/16  13/16  39/40  33/36 | Scarpinio et al. 2012 |
| 126 | 126 | 18  31.1 | 1/3  1/2  1/1 | vanBargen et al. 2012 |
| 290  5290 | 3100  118500 |  | 78/?  90/? | Gromadzka et al. 2016 ^a^ |

^a^ = reported only means of all samples

| **MON in barley** | | | | |
| --- | --- | --- | --- | --- |
| **149** | **350** |  | **88.6 %** |  |
| mean | max | min | n | References |
| 276.8 | 290 | 25 | 17/22 | Jestoi et al. 2004 |
|  | 380 | 130 | ?/83 | Uhlig et al. 2004 |
|  | 380 |  | ? | Sørensen et al. 2007 |
| 40 |  |  | 75/75 | EFSA CONTAM Panel 2014 |

| **MON in soybean products** | | | | |
| --- | --- | --- | --- | --- |
| **9** | **33** |  | **30.4 %** |  |
| mean | max | min | n | References |
| 9.4 | 33.34 | 0.12 | 7/23 | Fapohunda et al. 2018 ^b^ |

^b^ = these data have been obtained from outside of Europe.


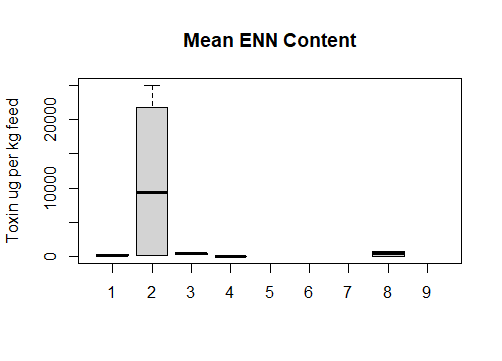


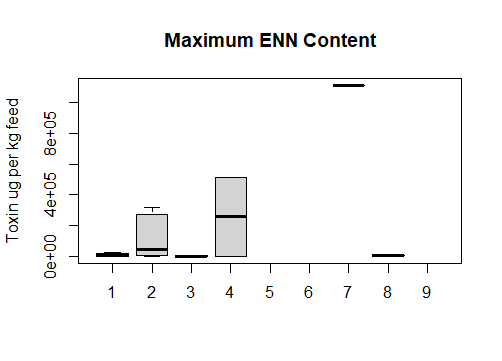


**Figure S9.** Mean (upper graph) and maximum (lower graph) content of enniatins (ENN) in feed ingredients (excluding outliers) according to the literature (listed in Annex III). 1 = wheat. 2 = corn, 3 = barley, 4 = soya, 5 = sunflower, 6 = canola, 7 = beans, 8 = DDGS, 9 = meat.

**Table S10** Literature data for enniatins (ENN) in feed ingredients (μg/kg), average values for all reported studies and the percentage of positive tested samples are shaded in light blue.

| **ENN in wheat** | | | | |
| --- | --- | --- | --- | --- |
| **174** | **67514** |  | **85.2** |  |
| mean | max | min | n | References |
|  | 136000 | 6 | 12/14 | Logrieco et al. 2002 |
|  | 24830 | 5.4 | 14/19 | Jestoi et al. 2004 |
|  | 7620 | 12 | 80/80 | Uhlig et al. 2006 |
|  | 2397 | 16.18 | 9/9 | Jestoi et al. 2008 |
|  | 634850 | 140 | 13/21 | Meca et al. 2010 |
|  | 325.1 | 30.37 | 18/57 | Juan et al. 2013 |
| 79.4 | 215.5 | 12.2 | 18/43 | Alkadri et al. 2014 |
| 126  730  297 |  |  | 12/12  34/34  34/34 | EFSA CONTAM Panel 2014 |
| 4  27  133  284  727 | 10  184  241  2400  1097 | 2  5  57  30  179 | 18/18  30/36  18/18  18/18  9/9 | Bryła et al. 2016 |

| **ENN in corn** | | | | |
| --- | --- | --- | --- | --- |
| **4033** | **202571** |  | **81.2** |  |
| mean | max | min | n | References |
|  | 46000 |  | 15/27 | Chelkowski et al. 2007 |
|  | 3307 | 44 | 70/73 | Sørensen et al. 2008 |
| 70  160 | 1210  2960 |  | 18/44  12/40 | Goertze et al. 2010 |
|  | 823651 | 450 | 25/28 | Meca et al. 2010 |
| 25040  18580 | 225170  315700 |  | ?/78  ?/90 | Gromadzka et al. 2016 ^a^ |
|  |  | 2  4  4  3  1 | 2/2  1/1  1/1  1/1  3/3 | Dzuman et al. 2017 Supplementary Data |

^a^ = reported only means of all samples

| **ENN in barley** | | | | |
| --- | --- | --- | --- | --- |
| **233** | **40496** |  | **93.9 %** |  |
| Mean | max | min | n | References |
|  | 18430 | 5.4 | 22/22 | Jestoi et al. 2004 |
|  | 5800 | 12 | 80/80 | Uhlig et al. 2006 |
|  | 735.8 | 16.18 | 29/29 | Jestoi et al. 2008 |
|  | 335850 | 450 | 2/4 | Meca et al. 2010 |
|  | 67.3 | 12.1 | 8/9 | Juan et al. 2013 |
| 363 |  |  | 75/75 | EFSA CONTAM Panel 2014 |
| 696.7  231.42 | 2078.86  601.42 | 33.26  31.32 | 22/22  6/6 | Bolechova et al. 2015 |
| 202  339 | 350  549 | 58  191 | 16/16  8/8 | Bryła et al. 2016 |

| **ENN in soybean products** | | | | |
| --- | --- | --- | --- | --- |
| **0.01** | **257000** |  | **54.3 %** |  |
| Mean | max | min | n | References |
|  | 514000 |  | 1/1 | Wang et al. 2013 |
| 0.00525 | 0.0055 | 0.005 | 2/23 | Fapohunda et al. 2018 ^b^ |

^b^ = these data have been obtained from outside of Europe.

| **ENN in beans** | | | | |
| --- | --- | --- | --- | --- |
| **?** | **1113000** |  | **100 %** |  |
| Mean | max | min | n | References |
|  | 1113000 |  | 1/1 | Wang et al. 2013 |

^b^ = these data have been obtained from outside of Europe.

| **ENN in DDGS** | | | | |
| --- | --- | --- | --- | --- |
| **366** | **1830** |  | **92.9 %** |  |
| mean | max | min | n | References |
| 730 | 1830 | <10 | 6/7 | Mortensen and Granby 2014 |
| 2 |  |  | 1/1 | Dzuman et al. 2017 Supplementary Data |


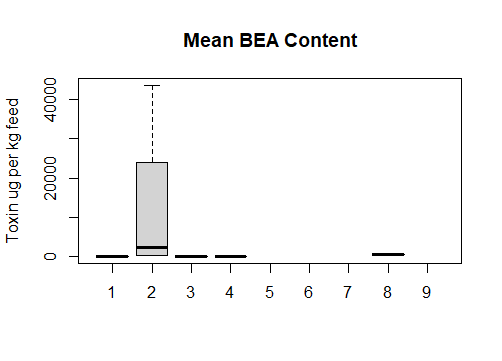


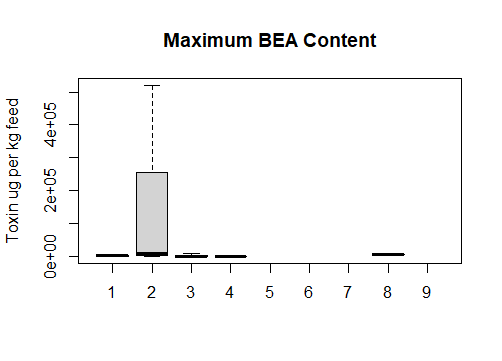


**Figure S10.** Mean (upper graph) and maximum (lower graph) content of beauvericin (BEA) in feed ingredients (excluding outliers) according to the literature (listed in Annex III). 1 = wheat. 2 = corn, 3 = barley, 4 = soya, 5 = sunflower, 6 = canola, 7 = beans, 8 = DDGS, 9 = meat.

**Table S10** Literature data for beauvericin (BEA) in feed ingredients (μg/kg), average values for all reported studies and the percentage of positive tested samples are shaded in light blue.

| **BEA in wheat** | | | | |
| --- | --- | --- | --- | --- |
| **22.7** | **1791** |  | **68.6 %** |  |
| mean | max | min | n | References |
|  | 3500 |  | ? | Kostecki et al. 1997 |
|  | 3703  3500 | 20 | ?  14/14 | Logrieco et al. 2002 |
| 10 |  |  | 13/14 | Jestoi et al. 2004 |
|  | 0.8 | 0.3 | 10/80 | Uhlig et al. 2006 |
|  |  | 10 | 8/9 | Jestoi et al. 2008 |
|  | 3500 | 170 | 9/21 | Meca et al. 2010 |
|  | 35 | 9.6 | 5/57 | Juan et al. 2013 |
| 2.7 | 5.1 | 1.8 | 6/43 | Alkadri et al 2014 |
| 1.5  1.5  1.5 |  |  | 12/12  34/34  34/34 | EFSA CONTAM Panel 2014 |

| **BEA in corn** | | | | |
| --- | --- | --- | --- | --- |
| **3611** | **2299903** |  | **52.3 %** |  |
| mean | max | min | n | References |
|  | 60000 | <1000 | 13/14 | Logrieco et al. 1993 |
|  | 10000 | <3000 | 4/6 | Bottalico et al. 1995 |
|  | 36890 | 1800 | 12/12 | Kostecki 1995 |
|  | 520000 | 100 | 6/22 | Ritieni et al. 1997 |
|  | 450 | 50 | 3/99 | Noser et al. 2001 |
|  | 1864000 | 10 | 19/209 | Jurjevic et al. 2002 |
|  | 3000 |  | 1/22 | Srobarova et al. 2002 |
|  | 988  496 | 7  7 | 45/73  5/7 | Sørensen et al. 2008 |
| 390  240 | 6402  5100 |  | 23/44  9/40 | Goertze et al. 2010 |
|  | 9310 | 172 | 6/28 | Meca et al. 2010 |
| 4330  43670 | 256500  445500 |  | ?/78  ?/90 | Gromadzka et al. 2016 |
|  |  | 1  1 | 2/2  2/2 | Dzuman et al. 2017 Supplementary Data |

| **BEA in barley** | | | | |
| --- | --- | --- | --- | --- |
| **31.4** | **1747** |  | **67.6 %** |  |
| mean | max | min | n | References |
|  | 19 | 10 | 22/22 | Jestoi et al. 2004 |
|  | 21 | 3 | 14/75 | Uhlig et al. 2006 |
|  |  | 10 | 27/29 | Jestoi et al. 2008 |
|  | 6940 | 170 | 2/4 | Meca et al. 2010 |
|  |  | 7.3 | 1/9 | Juan et al. 2013 |
| 1.5 |  |  | 75/75 | EFSA CONTAM Panel 2014 |
| 18.2 | 47.91 | 5.13 | 22/22 | Bolechova et al. 2015 |

| **BEA in soybean products** | | | | |
| --- | --- | --- | --- | --- |
| **3.69** | **23.04** |  | **56.5 %** |  |
| mean | max | min | n | References |
| 3.69 | 23.04 | 0.018 | 13/23 | Fapohunda et al. 2018 ^b^ |

^b^ = these data have been obtained from outside of Europe.

| **BEA in DDGS** | | | | |
| --- | --- | --- | --- | --- |
| **176** | **4220** |  | **99.2 %** |  |
| mean | max | min | n | References |
| 350 | 4220 | 40 | 58/59 | Tansakul et al. 2013 |
|  |  | 2 | 1/1 | Dzuman et al. 2017 Supplementary Data |
