# Supplementary material for: Risk assessment for mycotoxin contamination in fish feeds in Europe
Source: Mycotoxin Res. 2019 Jul 26;36(1):41–62. doi: 10.1007/s12550-019-00368-6 (PMC6971146; doi:10.1007/s12550-019-00368-6)
Supplement: Supplementary file 3 — (DOCX 47 kb) [file 12550_2019_368_MOESM3_ESM.docx]

Annex III: References for the Mycotoxin Occurrence

Abrunhosa L, Moraless H, Soares C, Calado T, Vila-Chã AS, Pereira M, Venâncio A (2016) A review of mycotoxins in food and feed products in Portugal and estimation of probable daily intakes, Crit Rev Food Sci Nutr 56:249–265.

Adler A, Lew H, Brodacz W, Edinger W, Oberforster M (1995) Occurrence of moniliformin, deoxynivalenol and zearalenone in durum wheat (*Triticum durum* Desf.). Mycotox Res 11:9–15.

Abudabos AM, Atiyat RMA, Khan RU (2017) A survey of mycotoxin contamination and chemical composition of distiller’s dried grains with solubles (DDGS) imported from the USA into Saudi Arabia Environ Sci Pollut Res 24:15401–15405.

Aldana JR, Silva LJG, Pena A, Mañes J, Lino CM (2014) Occurrence and risk assessment of zearalenone in flours from Portuguese and Dutch markets. Food Contr 45:51–55.

Alexa E, Dehelean CA, Poiana MA, Radulov I, Cimpean AM, Bordean DM, Tulcan C, Pop G (2013) The occurrence of mycotoxins in wheat from western Romania and histopathological impact as effect of feed intake. Chem Cent J 7:99.

Banu I, Aprodu I, Nicolau AI (2011) Occurrence of fusarium mycotoxins (deoxynivalenol and zearalenone) in wheat and high fibre wheat bread in Eastern Romania. J Environ Prot Ecol 12(2):519–525.

Beg MU, Al-Mutairi M, Beg KR, Al-Mazeedi HM, Ali LN, Saeed T (2006) Mycotoxins in poultry feed in Kuwait. Arch Environ Contam Toxicol 50:594–602.

Berthiller F, Dallásta C, Corradini C, Marchelli R, Sulyok M, Krska R, Adam G, Schuhmacher R (2009) Occurrence of deoxynivalenol and its 3-β-D-glucoside in wheat and maize. Food Addit Contam A 26(4):507–511.

Binder EM, Tan LM, Chin LJ, Handl J, Richard J (2007) Worldwide occurrence of mycotoxins in commodities, feeds and feed ingredients. Anim Feed Sci Technol 137(3-4):265–282.

Biselli S, Hummert C (2005) Development of a multicomponent method for Fusarium toxins using LCMS/MS and its application during a survey for the content of T-2 toxin and deoxynivalenol in various feed and food samples. Food Addit Contam 22(8):752–760.

Bryła M, Waskiewicz A, Podolska G, Szymczyk K, Jedrzejczak R, Damaziak K, Sułek A (2016) Occurrence of 26 mycotoxins in the grain of cereals cultivated in Poland. Toxins 8:160.

Chelkowski J, Zajkowki P, Zawadzki M, Perkowski J (1987) Moniliformin, deoxynivalenol, 3acetyldeoxynivalenol and zearalenone - Mycotoxins associated with corn cob fusariosis in Poland. Mycotox Res 3(Suppl 1):25–27.

Chelkowski J (1998) Distribution of Fusarium species and their mycotoxins in cereal grains. In: Sinha K.K., D. Bhatnager (eds.), Mycotoxins in Agriculture and Food Safety. Marcel Dekker, New York, pp. 45–64.

Chelkowski J, Gromadzka K, Stępień Ł, Lenc L, Kostecki M, Berthiller F (2012) Fusarium species, zearalenone and deoxynivalenol content in preharvest scabby wheat heads from Poland. World Mycotox J 5(2):133–141.

Cunha SC, Fernandes JO (2010) Development and validation of a method based on a QuEChERS procedure and heart-cutting GC-MS for determination of five mycotoxins in cereal products. J Sep Sci 33:600–609.

Curtui V, Usleber E, Dietrich R, Lepschy J, Martlauber E (1998) A survey on the occurrence of mycotoxins in wheat and maize from western Romania. Mycopathol 143(2):97–103.

Czerwiecki L, Czajkowska D, Witkowska-Gwiazdowska A (2002a) On ochratoxin A and fungal flora in polish cereals from conventional and ecological farms. Part 1: occurrence of ochratoxin A and fungi in cereals in 1997. Food Addit. Contam. 19:470–477.

Czerwiecki L, Czajkowska D, Witkowska-Gwiazdowska A (2002b) On ochratoxin A and fungal flora in polish cereals from conventional and ecological farms. Part 2: occurrence of ochratoxin A and fungi in cereals in 1998. Food Addit. Contam 19:1051–1057.

De Boevre, M, Di Mavungu JD, Maene P, Audenaert K, Deforce D, Haesaert G, Eeckhout M, Callebaut A, Berthiller F, Van Peteghem C, De Saeger S (2012) Development and validation of an LC-MS/MS method for the simultaneous determination of deoxynivalenol, zearalenone, T-2-toxin and some masked metabolites in different cereals and cereal-derived food. Food Addit Contam A*,* 29(5):819–835.

Deng SX, Tian LX, Liu FJ, Jin SJ, Liang GY, Yang HJ, Du ZY, Liu YJ (2010) Toxic effects and residue of aflatoxin B1 in tilapia (*Oreochromis niloticus* × *O. aureus*) during long-term dietary exposure. Aquacult 307:233–240.

Domijan AM, Peraica M, Jurjevic Z, Ivic D, Cvjetkovic B (2005) Fumonisin B1, fumonisin B2, zearalenone and ochratoxin A contamination of maize in Croatia. Food Addit Contam 22:677–680.

Dorn B, Forrer HR, Jenny E, Wettstein FE, Bucheli TD, Vogelgsang S (2011) Fusarium species complex and mycotoxins in grain maize from maize hybrid trials and from grower’s fields. J Appl Microbiol 111:693–706.

Edwards SG (2009) *Fusarium* mycotoxin content of UK organic and conventional wheat. Food Addit Contam A 26(4):496–506.

EFSA CONTAM Panel (EFSA Panel on Contaminants in the Food Chain) (2014) Scientific Opinion on the risks for human and animal health related to the presence of modified forms of certain mycotoxins in food and feed. EFSA J 12(12):3916, 107 pp. doi:10.2903/j.efsa.2014.3916.

Fapohunda SO, Anjorin TS, Sulyok M, Krska R (2018) Profile of major and emerging mycotoxins in sesame and soybean grains in the Federal Capital Territory, Abuja, Nigeria. Europ J Biol Res 8(3):121–130.

Filek G, Lindner W (1996) Determination of the mycotoxin moniliformin in cereals by high-performance liquid chromatography and fluorescence detection. J Chromatogr A 732:291–298.

Galbenu P, Damiescu L, Trif A (2011a) Zearalenone occurrence in cereal and cereal-based foodstuffs marketed in Timis county. Res J Agricult Sci 43(1):43–49.

Galbenu P, Damiescu L, Trif A (2011b) Fumonisin occurrence in cereal and cereal-based foodstuffs marketed in Timis county. Res J Agricult Sci 43(1):50–55.

Galbenu-Morvay PL, Trif A, Damiescu L, Simion G (2011) T-2 Toxin occurrence in cereals and cereal-based foods. Bulletin UASVM Agricult 68(2):274–280.

Hajšlova J, Lancova K, Sehnalova M, Krplova A, Zachariašova M, Moravcova H, Nedělnik J, Markova J, Ehrenbergerova J (2007) Occurrence of trichothecene mycotoxins in cereals harvested in the Czech Republic. Czech J Food Sci 25(6):339–350.

Ibáñez-Vea M, González-Peñas E, Lizarraga E, López de Cerain A (2012a) Co-occurrence of aflatoxins, ochratoxin A and zearalenone in barley from a northern region of Spain. Food Chem 132:35–42.

Ibáñez-Vea M, Lizarraga E, González-Peñas E, López de Cerain A (2012b) Co-occurrence of type-A and type-B trichothecenes in barley from a northern region of Spain. Food Contr 25:81–88.

Jaimez J, Fente CA, Franco CM, Cepeda A, Vazquez B (2004) A survey of the fungal contamination and presence of ochratoxin A and zearalenone on Spanish feed and raw materials. J Sci Food Agric 84:832–840.

Jajić I, Jurić V, Glamočić D, Abramović, B (2008) Occurrence of deoxynivalenol in maize and wheat in Serbia. Int J Mol Sci 9:2114–2126.

Jestoi M (2008) Emerging *Fusarium* mycotoxins fusaproliferin, beauvericin, enniatins and moliniformin – a review. Crit Rev Food Sci Nutr 48:21–49.

Jestoi M, Rokka M, Yli-Mattila T, Parikka P, Rizzo A, Peltonen K (2004) Presence and concentrations of the *Fusarium*-related mycotoxins beauvericin, enniatins and moniliformin in Finnish grain samples. Food Addit Contam 21:794–802.

Jørgensen K*,* Jacobsen JS *(*2002) Occurrence of ochratoxin A in Danish wheat and rye, 1992-99. Food Addit Contam 19:1184–1189.

Juan C, Ritieni A, Mañes J (2012) Determination of trichothecenes and zearalenones in grain cereal, flour and bread by liquid chromatography tandem mass spectrometry. Food Chem 134*:*2389–2397.

Juan C, Ritieni A, Mañes J (2013) Occurrence of Fusarium mycotoxins in Italian cereal and cereal products from organic farming. Food Chem 141:1747–1755.

Jurjevic Z, Solfrizzo M, Cvjetkovic B, De Girolamo A, Visconti A (2002) Occurrence of beauvericin in corn from Croatia. Food Technol Biotechnol 40:91–94.

Kirinčič S, Škrjanc B, Kos N, Kozolc B, Pirnat N, Tavčar-Kalcher G (2015) Mycotoxins in cereals and cereal products in Slovenia official control of foods in the years 2008-2012. Food Contr 50:157–165.

Klarić MS, Cvetnić Z, Pepeljnjak S, Kosalec I (2009) Co-occurrence of aflatoxins, ochratoxin a, fumonisins, and zearalenone in cereals and feed determined by competitive direct enzyme-linked immunosorbent assay and thin-layer chromatography. Arh Hig Rada Toksikol 60:427–434.

Kosicki R, Błajet-Kosicka A, Grajewski J, Twarużek M (2016) Multiannual mycotoxin survey in feed materials and feedingstuffs. Anim Feed Sci Technol 215:165–180.

Kostecki. M, Szczesna J, Chelkowski J, Wisniewska H (1995) Beauvericin and moniliformin production by Polish isolates of *Fusarium subglutinans* and natural coocurrence of both mycotoxins in maize samples. Microbiol Alim Nutr 13:67–70.

Kostecki M, Grabarkiewicz-Szczesna J, Golinski P (1997) Simultaneous analysis of beauvericin and moniliformin in fungal cultures and in cereal grain samples. Mycotox Res 13:17–22.

Krysinska-Traczyk W, Kiecana I, Perkowski J, Dutkiewicz J (2001) Levels of fungi and mycotoxins in samples of grain and grain dust collected on farms in Eastern Poland. Ann Agr Env Med 8(2):269–274.

Langseth W, Rundberget T (1999) The occurrence of HT-2 toxin and other trichothecenes in Norwegian cereals. Mycopathol 147(3):157–165.

Lew H, Adler A, Edinger W (1991) Moniliformin and the European corn borer (*Ostrinia nubialis*). Mycotox Res 7A:71–76.

Lew H, Chelkowski J, Pronczuk P, Edinger W (1996) Occurrence of the mycotoxin moniliformin in maize (*Zea mays* L.) ears infected by *Fusarium subglutinans* (Wollenw. & Reinking) Nelson et al. Food Addit Contam 13(3):321–324.

Lindblad M, Gidlund A, Sulyok M, Börjesson T, Krska R, Olsen M, Fredlund E (2013) Deoxynivalenol and other selected Fusarium toxins in Swedish wheat - Occurrence and correlation to specific *Fusarium* species. Int J Food Microbiol 167:284–291.

Lino CM, Silva LJG, Pena ALS, Silveira MI (2006) Determination of fumonisins B1 and B2 in Portuguese maize and maize-based samples by HPLC with fluorescence detection. Anal Bioanal Chem 384:1214–1220.

Logrieco A, Moretti A, Ritieni A, Chelkowski J, Altomare C, Bottalico A, Randazzo G (1993) Natural occurrence of beauvericin in preharvest *Fusarium subglutinans* infected corn ears in Poland. J Agricult Food Chem 41:2149–2152.

Logrieco A, Moretti A, Ritieni A, Bottalico A, Corda P (1995) Occurrence and toxigenicity of *Fusarium proliferatum* from preharvest maize ear rot, and associated mycotoxins, in Italy. Plant Dis 79:727–731.

Logrieco A, Rizzo A, Ferracane L, Ritieni A (2002) Occurrence of beauvericin and enniatins in wheat affected by *Fusarium avenaceum* head blight. Appl Environ Microbiol 68(1):82–85.

Macri AM, Miclăuş V, Dancea Z, Morar MV, Paşca I, Scurtu I, Szakacs A, Rus V (2009) Zearalenone and trichothecene content of maize and wheat samples coming from center and western Romania. Annals of RSCB XIV(2):315–318. retrieved January 10, 2018 from http://www.annalsofrscb.ro/ content/14%202%20CONTENTS.pdf.

Mankevičienė A, Butkutė B, Dabkevičius Z, Supronienė S (2007) *Fusarium* mycotoxins in Lithuanian cereals from the 2004-2005 harvests. Ann Agric Environ Med 14:103–107.

Mankevičienė A, Butkutė B, Gaurilčikienė I, Dabkevičius Z, Supronienė S (2011) Risk assessment of *Fusarium* mycotoxins in Lithuanian small cereal grains. Food Contr 22:970–976.

Manova R, Mladenova R (2009) Incidence of zearalenone and fumonisins in Bulgarian cereal production. Food Contr 20:362–365.

Marin S, Ramos AJ, Cano-Sancho G, Sanchis V (2013) Mycotoxins: Occurence, toxicology, and exposure assessment. Food Chem Toxicol 60:218–237.

Martins HM, Marques M, Almeida I, Guerra MM, Bernardo F (2008) Mycotoxins in feedstuffs in Portugal: an overview. Mycotox Res 24:19–23.

McCormick SP, Stanley AM, Stover NA, Alexander NJ (2011) Trichothecenes: From Simple to Complex Mycotoxins. Toxins 3:802–814.

Meca G, Sospedra I, Soriano JM, Ritieni A, Valero MA, Mañes J (2009) Isolation, purification and antibacterial effects of fusaproliferin produced by *Fusarium subglutinans* in submerged culture. Food Chem Toxicol 47:2539–2543.

Miraglia E, Brera C (2002) Assessment of dietary intake of ochratoxin A by the population of EU member states. Scientific Cooperation Task 3.2.7 of the European Commission. Available from http://ec.europa.eu/food/fs/scoop/index_en.print.html.

Mişcă C, Damiescu L, Jianu C, David I, Mişcă L, Mărginean O, Radoi B, Rinovetz A, Bujanca G, Velciov A, Ianovici N (2014) The incidence of the strains of Fusarium sp. And of zearalenone in cereals analyzed from the South-West of Romania. Ann West Univ Timişoara, ser. Biology XVII (2):137-144. retrieved January 1, 2018 from http://www.biologie.uvt.ro/annals/vol_XVII_2.pdf.

Moura AM, Batoreu MC, Felgueiras MI (1998) A research of deoxynivalenol in wheat flour traded in Portugal. Eur J Pharm Sci 6:S44.

Müller HM, Schwadorf K (1993) A survey of the natural occurrence of *Fusarium* toxins in wheat grown in a southwestern area of Germany. Mycopathol 121:115–121.

Nacher-Mestre J, Serrano R, Beltrán E, Pérez-Sánchez J, Silva J, Karalazos V, Hernández F, Berntssen MHG (2015) Occurrence and potential transfer of mycotoxins in gilthead sea bream and Atlantic salmon by use of novel alternative feed ingredients. Chemosphere 128:314–320.

Nesheim S, Wood GE (1995) Regulatory aspects of mycotoxins in soybean and soybean products. J Am Oil Chem Soc 72(12):1421–1423.

Nordkvist E, Häggblom P (2014) Fusarium mycotoxin contamination of cereals and bedding straw at Swedish pig farms. Anim Feed Sci Technol 198:231–237.

Noser J, Wenk P, Sutter A, Frankhauser I, Hirschi H (2001) Fumonisine B1 und B2, Moniliformin, Beauvericin und Zearalenon in Mais auf dem Schweizer Markt. Mitt Lebensmittel Hyg 92:90–103.

Palermo D, Pietrobono P, Palermo C, Rotunno T (2002) Occurrence of ochratoxin A in cereals from Puglia (Italy). Ital J Food Sci 14:447–453.

Parich A, Schuch Boeira L, Perez Castro S, Krska R (2003) Determination of moniliformin using SAX columns clean-up and HPLC/DAD-detection. Mycotox Res 19:203–206.

Peito A, Venâncio A (2004) An overview of mycotoxins and toxigenic fungi in Portugal. In: Logrieco A & Visconti A (Eds.), Overview on Toxigenic Fungi and Mycotoxins in Europe, (pp. 173-184). The Netherlands, Dordrecht: Kluwer Academic Publishers.

Perkowski J, Wiwart M, Buśko M, Laskowska M, Berthiller F, Kandler W, Krska R (2007) Fusarium toxins and total fungal biomass indicators in naturally contaminated wheat samples from north-eastern Poland in 2003. Food Addit Contam 24(11):1292–1298.

Perkowski J, Stuper K, Buśko M, Góral T, Jeleń H, Wiwart M, Suchowilska E (2012) A comparison of contents of group A and B trichothecenes and microbial counts in different cereal species. Food Addit Contam B 5(3):151–159.

Piva G, Battilani P, Pietri A (2006) Emerging issues in southern Europe: Aflatoxins in Italy. In:The Mycotoxin Factbook, Food and Feed Topics; Barug, D., Bhatnagar, D., Eds.; Wageningen Academic Publishers: Wageningen, The Netherlands, pp. 139–153.

Placinta CM, D’Mello JPF, Macdonald AMC (1999) A review of worldwide contamination of cereal grains and animal feed with *Fusarium* mycotoxins. Anim Feed Sci Technol 78:21–37.

Pleadin J, Vahčić N, Perši N, Ševelj D, Markov K, Frece J (2013) *Fusarium* mycotoxins’ occurrence in cereals harvested from Croatian fields. Food Contr 32:49–54.

Prickett AJ, MacDonald S, Wildey KB (2000) Survey of mycotoxins in stored grain from the 1999 harvest in the U.K. Home-Grown Cereals Authority (HGCA) Project report no. 230.

Prieto-Simon B, Noguer T, Campas M (2007) Emerging biotools for assessment of mycotoxins in the past decade. Trac-Trend Anal Chem 26(7):689–702.

Rafai P, Árpád B, László J, András V (2000) Evaluation of mycotoxin-contaminated cereals for their use in animal feeds in Hungary. Food Addit Contam 17 (9):799–808.

Pussemier L, Piérard J-Y, Anselme M, Tangni EK, Motte J-C, Larondelle Y (2006) Development and application of analytical methods for the determination of mycotoxins in organic and conventional wheat. Food Addit Contam 23(11):1208–1218.

Rasmussen PH, Ghorbani F, Berg T (2003) Deoxynivalenol and other *Fusarium* toxins in wheat and rye flours on the Danish market. Food Addit Contam 20(4):396–404.

Reinhold L, Reinhardt K (2011) Mycotoxins in foods in Lower Saxony (Germany): results of official control analyses performed in 2009. Mycotox Res 27:137–143.

Richard JL, Stubblefield RD, Lyon RL, Peden WM, Thurston JR, Rimler RB (1986) Distribution and clearance of aflatoxins B1 and M1 in turkeys fed diets containing 50 or 150 ppb aflatoxin from naturally contaminated corn. Avian Dis 30:788–793.

Ritieni A, Moretti A, Logrieco A, Bottalico A, Randazzo G, Monti SM, Ferracane R, Fogliano V (1997) Occurrence of fusaproliferin, fumonisin B1, and beauvericin in maize from Italy*.* J Agric Food Chem 45:4011-4016.

Rodríguez-Carrasco Y, Ruiz MJ, Font G, Berrada H (2013) Exposure estimates to *Fusarium* mycotoxins through cereals intake. Chemosphere 93:2297–2303.

Rubert J, Soriano JM, Mañes J, Soler C (2013) Occurrence of fumonisins in organic and conventional cereal-based products commercialized in France, Germany and Spain. Food Chem Toxicol 56:387–391.

Santini A, Ferracane R, Somma MC, Aragon A, Ritieni A (2009) Multitoxin extraction and detection of trichothecenes in cereals: an improved LC-MS/MS approach. Sci Food Agric 89**:**1145–1153.

Schneeweis I, Meyer K, Engelhardt G, Bauer J (2002) Occurrence of zearalenone-4-β-D glucopyranoside in wheat. J Agric Food Chem 50:1736–1738.

Schollenberger M, Jara HT, Suchy S, Drochner W, Müler HM (2002) *Fusarium* toxins in wheat flour collected in an area in southwest Germany. Int J Food Microbiol 72:85–89.

SCOOP (2003) Collection of occurrence data of *Fusarium* toxins in food and assessment of dietary intake by the population of EU member states, Directorate-general health and protection. http://europa.eu.int/consumer comm/food/fs/scoop/task3210.pdf

Scudamore KA, Hetmanski MT, Chan HK, Collins S (1997) Occurrence of mycotoxins in raw ingredients used for animal feeding stuffs in the United Kingdom in 1992. Food Addit Contam A 14:157–173.

Scudamore KA, Nawaz S, Hetmanski MT (1998) Mycotoxins in ingredients of animal feeding stuffs: II. Determination of mycotoxins in maize and maize products. Food Addit Contam 15(1):30–55.

Scudamore KA, Patel S, Edwards SG (2009) HT-2 toxin and T-2 toxin in commercial cereal processing in the United Kingdom, 2004-2007. World Mycotox J 2*:*357–365.

Škrbić B, Malachova A, Živančev J, Veprikova Z, Hajšlová J (2011) Fusarium mycotoxins in wheat samples harvested in Serbia: A preliminary survey. Food Contr 22:1261–1267.

Škrbić B, Živančev J, Mladenović ND, Godula M (2012) Principal mycotoxins in wheat flour from the Serbian market: Levels and assessment of the exposure by wheat-based products. Food Contr 25:389–396.

Šliková S, Gavurníková S, Šudyová V, Gregová E (2013) Occurrence of deoxynivalenol in wheat in Slovakia during 2010 and 2011. Toxins 5:1353–1361.

Šliková S, Gavurníková S, Mináriková M, Gregová E, Šudyová V (2014) Mycotoxin survey of wheat samples graded according to their technological quality. Agric Food Sci 23:186–193.

Srobarova A, Moretti A, Ferracane R, Ritieni A, Logrieco A (2002) Toxigenic *Fusarium* species of Liseola section in pre-harvest maize ear rot and associated mycotoxins in Slovakia. Eu J Plant Pathol 108:299–306.

Soares C, Venancio A (2011) Mycotoxins in Post-harvest maize in three Portuguese regions. ISM Conference 2011. Mendoza, Argentina, p. 160.

Sørensen JL, Nielsen KF, Thrane U (2007) Analysis of moniliformin in maize plants using hydrophilic interaction chromatography. J Agric Food Chem 55:9764−9768.

Sørensen JL, Nielsen KF, Rasmussen PH, Thrane U (2008) Development of a LC-MS/MS method for the analysis of enniatins and beauvericin in whole fresh and ensiled maize. J Agric Food Chem 56:10439−10443.

Souza MLM, Sulyok M, Freitas-Silva SS, Costa C, Brabet M, Machnski J, Sekiyama BL, Azevedo Vargas E, Krska R, Schuhmacher R (2013) Co-occurrence of mycotoxins in maize and poultry feeds from Brazil by liquid chromatography/tandem mass spectrometry. Sci World J 1:1−9.

Stankovic S, Levic J, Ivanovic D, Krnjaja V, Stankovic G, Tanci S (2012) Fumonisin B1 and its co-occurrence with other fusariotoxins in naturally-contaminated wheat grain. Food Contr 23:384−388.

Streit E, Schatzmayr G, Tassis P, Tzika E, Marin D, Taranu I, Tabuc C, Nicolau A, Aprodu I, Puel O, Oswald IP (2012) Current situation of mycotoxin contamination and co-occurrence in animal feed - Focus on Europe. Toxins 4:788−809.

Stroia C, Tabuc C, Neacşu A (2010) Incidence of *Fusarium* species and its mycotoxins in cereals from Western Romania. Res J Agricult Sci 42(2):302−309.

Thalman A, Matzenauer S, Gruber-Schley S (1985) Untersuchungen über das Vorkommen von Fusarientoxinen in Getreide. Berichte über Landwirtschaft 63:257−272.

Uhlig S, Torp M, Jarp J, Parich A, Gutleb AC, Krska R (2004) Moniliformin in Norwegian grain. Food Addit Contam 21(6):598−606.

Uhlig S, Torp M, Heier BT (2006) Beauvericin and enniatin A, A1, B and B1 in Norwegian grain: a survey. Food Chem 94:193−201

Valenta H, Dänicke S, Blüthgen A (2002) Mycotoxins in soybean feedstuffs used in Germany. Mycotox Res 18(2):208−2011.

Van Der Fels-Klerx HJ, Klemsdal S, Hietaniemi V, Lindblad M, Ioannou-Kakouri E, Van Asselt ED (2012) Mycotoxin contamination of cereal grain commodities in relation to climate in North West Europe. Food Addit Contam A 29(10):1581−1592.

Vrabcheva T, Grebler R, Usleber E, Martlbauer E (1996) First survey on the natural occurrence of *Fusarium* mycotoxins in Bulgarian wheat. Mycopathol 136(1):47−52.

Yazar S, Omurtag GZ (2008) Fumonisins, trichothecenes and zearalenone in cereals. Int J Mol Sci 9:2062−2090.

Yli-Mattila T, Rämö S, Hussien T, Rauvola M, Hietaniemi V, Kaitaranta J (2017) Different grain grinding methods affect detection of *Fusarium graminearum* DNA and mycotoxins. Phytopathol Mediterr 56(1):167−174.

Zachariasova M, Dzuman Z, Veprikova Z, Hajkova K, Jiru M, Vaclavikova M, Zachariasova A, Pospichalova M, Florian M, Hajslova J (2014) Occurrence of multiple mycotoxins in European feedingstuffs, assessment of dietary intake by farm animals. Anim Feed Sci Technol 193:124–140.
