# Supplementary material for: Risk assessment for mycotoxin contamination in fish feeds in Europe
Source: Mycotoxin Res. 2019 Jul 26;36(1):41–62. doi: 10.1007/s12550-019-00368-6 (PMC6971146; doi:10.1007/s12550-019-00368-6)
Supplement: Supplementary file 4 — (DOCX 227 kb) [file 12550_2019_368_MOESM4_ESM.docx]

Annex IV: Toxicity of Mycotoxins in Fish

**Table S11** Literature data for aflatoxin B_1_ (AFB_1_) toxicity in fish as log 10 values of the reported no effect levels (NOEL) and lowest effect levels (LOEL) reported in the studies listed in detail in Annex V, including beluga (*Huso huso*), catla (*Catla catla*) channel catfish (*Ictalurus punctatus*), common carp (*Cyprinus carpio*), gibel carp (*Carassius auratus gibelio*), guppy (*Poecilia reticulata*), matrinxã (*Brycon cephalus*), medaka (*Oryzias latipes*), mosquitofish (*Gambusia affinis*), sea bass (*Dicentrarchus labrax*), silver catfish (*Rhamdia quelen*), tilapia species (*Oreochromis niloticus*, *O. mossambicus*, and hybrids), rainbow trout (*Oncorhynchus mykiss*), red drum (*Sciaenops ocellatus*), rohu (*Labeo rohita*), Tra catfish (*Pangasius hypophthalmus*), walleye (*Sander vitreus*), yellow catfish (*Pelteobagrus fulvidraco*), and zebrafish (*Danio rerio*).

| **AFB1 toxicity** | | | | |
| --- | --- | --- | --- | --- |
| **Species** | **log LOEL** | **log NOEL** | **endpoint** | References |
| hybrid tilapia | 3.22 |  | behaviour | Deng et al. 2010 |
| sea bass | 1.26 | 3.00 |  | El-Sayed and Khalil 2009 |
| Tra catfish | 2.67 | 2.37 |  | Goncalves et al. 2018a |
| gibel carp |  |  |  | Huang et al. 2011 |
| Nile tilapia | 3.60 |  |  | Hussain et al. 2017 |
| rohu | 4.06 | 3.00 |  | Sahoo et al. 2003 |
| rainbow trout | 1.90 | 1.30 | biotransformation | Breinholt et al. 1995 |
| rainbow trout | 1.10 |  |  | Dashwood et al. 1991 |
| hybrid tilapia | 1.93 |  |  | Deng et al. 2010 |
| Nile tilapia | 3.78 |  |  | El-Barbary et al. 2018 |
| Nile tilapia | 2.30 |  |  | Mafouz 2015 |
| rainbow trout | 1 |  |  | Nixon et al. 1984 |
| rainbow trout | 0.60 |  |  | Shelton et al. 1984 |
| Mozambique tilapia | 2.57 |  |  | Varior and Philip 2012 |
| common carp | 3.78 |  | blood | Al-Faragi et al. 2014 |
| common carp | 3.60 |  |  | Al-Faragi et al. 2014 |
| Nile tilapia | 3.30 |  |  | Ayyat et al. 2018 |
| Nile tilapia | 2.40 |  |  | Ayyat et al. 2014 |
| hybrid tilapia | 2.90 |  |  | Deng et al. 2010 |
| Nile tilapia | 3.78 |  |  | El-Barbary et al. 2016 |
| Nile tilapia | 2.30 |  |  | El-Boshy et al. 2008 |
| sea bass | 1.26 |  |  | El-Sayed and Khalil 2009 |
| Tra catfish | 1.79 |  |  | Goncalves et al. 2018a |
| channel catfish | 4.08 |  |  | Jantrarotai et al. 1990 |
| Nile tilapia | 2.00 | 1.30 |  | Mafouz and Sherif 2015 |
| channel catfish |  | 2.3 |  | Manning et al. 2005 |
| rohu | 1 |  |  | Mohapatra et al. 2011 |
| Nile tilapia | 2.3 |  |  | Rahman et al. 2017 |
| rainbow trout |  | 3.0 |  | Saei et al. 2017 |
| Nile tilapia | 2.30 |  |  | Selim et al. 2014 |
| rohu | 3.10 |  |  | Sahoo and Mukherjee 2001 |
| rohu | 3.10 |  |  | Sahoo and Mukherjee 2002 |
| common carp | 1.30 |  |  | Svobodova et al. 1982 |
| Nile tilapia | 3.40 | 2.40 |  | Tuan et al. 2002 |
| yellow catfish | 3.07 |  |  | Wang et al. 2016 |
| hybrid tilapia | 2.90 |  | body coloration | Deng et al. 2010 |
| Nile tilapia | 2.30 |  |  | Hegazi et al. 2013 |
| catla | 1 |  | body composition | Andleeb et al. 2015 |
| Nile tilapia |  | 2.40 |  | Ayyat et al. 2013 |
| hybrid tilapia | 2.90 |  |  | Deng et al. 2010 |
| hybrid tilapia | 1.93 |  |  | Deng et al. 2010 |
| Tra catfish | 2.67 |  |  | Goncalves et al. 2018a |
| Nile tilapia | 3.30 |  |  | Hussain et al. 2017 |
| rainbow trout | -1.30 | -1.60 |  | Imani et al. 2017 |
| Nile tilapia | 2.18 |  |  | Magouz 2016 |
| Nile tilapia | 2.00 | 1.30 |  | Mafouz and Sherif 2015 |
| Nile tilapia | 1.30 |  |  | Marijani et al. 2017 |
| Nile tilapia |  | 3.48 |  | Zychowski et al. 2013a |
| red drum | 3.30 | 2.00 |  | Zychowski et al. 2013b |
| rainbow trout | 3.60 |  | cancer | Ayres et al. 1971 |
| rainbow trout | 1.30 |  |  | Bailey et al. 1989 |
| rainbow trout | 0.60 |  |  | Bailey et al. 1994 |
| rainbow trout | 1.30 |  |  | Bailey et al. 1994 |
| rainbow trout | 2.23 |  |  | Bailey et al. 1998 |
| rainbow trout | 3.52 |  |  | Bailey et al. 1996 |
| rainbow trout | 2.74 |  |  | Bastardo et al. 2006 |
| rainbow trout | 3.78 |  |  | Black et al. 1985 |
| rainbow trout | 1.90 |  |  | Curtis et al. 2995 |
| rainbow trout | 1.82 |  |  | Dashwood et al. 1994 |
| beluga | 4.00 |  |  | Farabi et al. 2006 |
| rainbow trout | 1.00 |  |  | Goeger et al. 1988 |
| rainbow trout | 2.70 |  |  | Goeger et al. 1988 |
| rainbow trout | -0.30 |  |  | Halver 1969 |
| medaka | 3.70 |  |  | Hatanaka et al. 1982 |
| medaka | 3.40 |  |  | Hatanaka et al. 1982 |
| Nile tilapia | 2.30 |  |  | Hegazi et al. 2013 |
| rainbow trout | 2.70 |  |  | Hendricks et al. 1980 |
| rainbow trout | 2.70 |  |  | Hendricks et al. 1994 |
| rohu | 3.00 |  |  | Madhusudhanan et al. 2006 |
| rainbow trout | 1.3 |  |  | Nixon et al. 1984 |
| rainbow trout | 2.7 |  |  | Nunez et al. 1991 |
| rainbow trout | 1.7 |  |  | Oganesian et al. 1999 |
| rainbow trout | 1.0 |  |  | Orner et al. 1993 |
| guppy | 2.78 |  |  | Sato et al 1973. |
| rainbow trout | 1.30 |  |  | Schoenhard et al. 1981 |
| rainbow trout | 1.30 |  |  | Sinnhuber et al. 1984 |
| rainbow trout | 0.60 |  |  | Sinnhuber et al. 1968 |
| rainbow trout | 2.60 |  |  | Spring and Fegan 2010 |
| rainbow trout | 2.40 |  |  | Thorgaard et al. 1999 |
| rainbow trout | 1.70 |  |  | Tilton et al. 2005 |
| rainbow trout | 2.70 |  |  | Wales et al. 1978 |
| rainbow trout | 2.00 |  |  | Zhang et al. 1992 |
| rainbow trout | 2.60 |  | genotoxicity | Carpenter et al. 1995 |
| rainbow trout | 2.7 |  |  | Abd-Allah et al. 1999 |
| channel catfish |  | 2.7 |  | Abd-Allah et al. 1999 |
| zebrafish | 1.70 |  |  | Troxel et al. 1997 |
| common carp | 3.60 |  | growth | Al-Faragi et al. 2014: |
| catla | 1 |  |  | Andleeb et al. 2015 |
| catla | 1.48 | 1.30 |  | Andleeb et al. 2015 |
| catla | 1.00 |  |  | Andleeb et al. 2015 |
| catla | 1.30 | 1.00 |  | Andleeb et al. 2015 |
| Nile tilapia | 3.30 |  |  | Ayyat 2018 |
| Nile tilapia | 2.40 |  |  | Ayyat et al. 2014. |
| rohu |  | 2.18 |  | Baglodi et al. 2015 |
| rohu | 2.18 | 2 |  | Baglodi et al. 2015 |
| matrinxã | 1.02 |  |  | Bedoya-Sernal et al. |
| Nile tilapia | 3.27 | 4.48 |  | Chavez-Sanchez et al. 1994 |
| hybrid tilapia | 3.22 | 2.39 |  | Deng et al. 2010 |
| hybrid tilapia | 2.39 | 1.93 |  | Deng et al. 2010 |
| Nile tilapia | 2.00 |  |  | El-Banna et al. 1992 |
| Tra catfish | 1.79 |  |  | Goncalves et al. 2018a |
| Tra catfish | 2.39 |  |  | Goncalves et al. 2018a |
| Tra catfish | 2.67 |  |  | Goncalves et al. 2018a |
| gibel carp | 1.25 | 1.10 |  | Han et al. 2009 |
| gibel carp |  | 1.46 |  | Han et al. 2009 |
| Nile tilapia | 2.30 |  |  | Hegazi et al. 2013 |
| gibel carp | 1.74 | 1.05 |  | Huang et al. 2011 |
| gibel carp | 1.74 | 1.31 |  | Huang et al. 2011 |
| gibel carp | 1.74 | 0.51 |  | Huang et al. 2011 |
| Nile tilapia | 3.30 |  |  | Huang et al. 2012 |
| Nile tilapia | 3.60 |  |  | Hussain et al. 2017. |
| common carp | 3.78 |  |  | Al-Faragi et al. 2014: |
| channel catfish | 4.00 | 3.33 |  | Jantrarotai and Lovell 1990 |
| Mozambique tilapia | 2.00 |  |  | Lim et al. 2001 |
| silver catfish |  | 3.07 |  | Lopes et al. 2009 |
| silver catfish | 2.31 | 1.95 |  | Lopes et al. 2009 |
| silver catfish | 2.18 |  |  | Lopes et al. 2009 |
| Nile tilapia | 2.18 |  |  | Magouz 2016 |
| Nile tilapia | 2.00 | 1.30 |  | Mafouz and Sherif 2015 |
| channel catfish |  | 2.3 |  | Manning et al. 2005 |
| channel catfish |  | 2.3 |  | Manning et al. 2005 |
| Nile tilapia | 4 |  |  | Nguyen et al. 2002 |
| rohu |  | 3.10 |  | Sahoo and Mukherjee 2002 |
| rohu | 4.06 |  |  | Sahoo et al. 2003 |
| Nile tilapia | 2.30 |  |  | Selim et al. 2014 |
| beluga | 1.70 | 1.40 |  | Sepahdari et al. 2010 |
| beluga | 1.88 |  |  | Sepahdari et al. 2010 |
| Nile tilapia | 1.30 |  |  | Sherif et al. 2013. |
| rainbow trout | 0.60 |  |  | Sinnhuber et al. 1968 |
| Nile tilapia | 3.40 | 2.40 |  | Tuan et al. 2002 |
| yellow catfish | 3.07 |  |  | Wang et al. 2016 |
| zebrafish | 2.19 |  |  | Weigt et al. 2011 |
| Nile tilapia | 3.18 |  |  | Zychowski et al. 2013a |
| red drum | 2.00 |  |  | Zychowski et al. 2013b |
| common carp | 3.60 |  | histology | Al-Faragi et al. 2014: |
| catla | 1.30 | 1.00 |  | Andleeb et al 2015 |
| rainbow trout | 1.60 |  |  | Arana et al. 2002 |
| hybrid tilapia | 2.80 | 2.39 |  | Deng et al. 2010 |
| Nile tilapia | 2.30 |  |  | El-Banna et al. 1992 |
| Nile tilapia | 3.78 |  |  | El-Barbary et al. 2018 |
| gibel carp | 1.46 | 1.25 |  | Han et al. 2009 |
| Nile tilapia | 2.30 |  |  | Hegazi et al. 2013 |
| gibel carp |  | 3.00 |  | Huang et al. 2011 |
| Nile tilapia |  | 3.60 |  | Hussain et al. 2017 |
| walleye | 1.70 |  |  | Hussain et al. 1993 |
| common carp | 3.78 |  |  | Al-Faragi. et al 2014 |
| silver catfish | 2.88 | 2.54 |  | Lopes et al. 2009 |
| Nile tilapia | 2.00 | 1.30 |  | Mafouz and Sherif 2015 |
| channel catfish |  | 2.3 |  | Manning et al. 2005 |
| Nile tilapia | 1.30 |  |  | Marijani et al. 2017 |
| rainbow trout | 2.4 | 2 |  | Nunez et al. 1990. |
| beluga | 1.88 |  |  | Sepahdari et al 2010 |
| common carp | 2.70 |  |  | Shahafve et al. 2017 |
| common carp | 1.30 |  |  | Svobodova and Piskac 1980 |
| rainbow trout | 1.70 |  |  | Tilton et al. 2005. |
| Nile tilapia | 4.00 | 3.40 |  | Tuan et al. 2002 |
| Mozambique tilapia | 3.40 |  |  | Varior and Philip 2012 |
| Nile tilapia | 3.18 |  |  | Zychowski et al. 2013a |
| red drum | 2.70 | 2.40 |  | Zychowski et al. 2013b |
| zebrafish | 1.10 |  |  | Zhou et al. 2017 |
| Nile tilapia | 3.30 |  | immune responses | Ayyat 2018 |
| Nile tilapia | 2.30 |  |  | El-Boshy et al. 2008 |
| Tra catfish | 1.79 | 2.18 |  | Goncalves et al. 2018a |
| Tra catfish | 2.37 |  |  | Goncalves et al. 2018a |
| Tra catfish | 2.37 | 1.79 |  | Goncalves et al. 2018a |
| Nile tilapia | 3.48 |  |  | Hussein et al. 2000 |
| Nile tilapia | 0.00 |  |  | Hussein et al. 2000 |
| Nile tilapia | 1.70 |  |  | Hussein et al. 2000 |
| channel catfish | 4.00 |  |  | Jantrarotai and Lovell 1990 |
| Nile tilapia | 2.00 | 1.30 |  | Mafouz and Sherif 2015 |
| Nile tilapia | 2.30 |  |  | Mafouz 2015 |
| Nile tilapia | 1.30 |  |  | Marijani et al. 2017 |
| rohu | 1 |  |  | Mohapatra et al. 2011 |
| rainbow trout | 2.7 |  |  | Ottinger et al. 1999 |
| Nile tilapia | 2.3 |  |  | Rahman et al. 2017 |
| rohu | 3.10 |  |  | Sahoo and Mukherjee 2001 |
| rohu | 3.10 |  |  | Sahoo and Mukherjee 2002 |
| rohu | 3.10 |  |  | Sahoo andMukherjee 2003 |
| rohu |  | 3.10 |  | Sahoo and Mukherjee 2003 |
| rohu | 3.10 |  |  | Sahoo and Mukherjee 2003 |
| rohu | 3.88 |  |  | Sahoo et al. 2003 |
| rohu | 4.10 |  |  | Sahoo et al. 2003 |
| Nile tilapia | 2 |  |  | Sherif et al. 2013 |
| yellow catfish | 3.07 |  |  | Wang et al. 2016 |
| Nile tilapia | 3.18 | 3.48 |  | Zychowski et al. 2013a |
| red drum | 2.40 | 2.00 |  | Zychowski et al. 2013b |
| catla | 1.60 | 1.48 | mortality | Andleeb et al. 2015 |
| rainbow trout |  | 1.90 |  | Arana et al. 2002 |
| rohu |  | 2.18 |  | Baglodi et al. 2015 |
| rainbow trout | 2.91 |  |  | Bauer et al. 1969 |
| matrinxã |  | 1.751817788 |  | Bedoya-Sernal et al. 2018 |
| Nile tilapia | 1.59 |  |  | Cagauan et al. 2004 |
| hybrid tilapia |  | 3.22 |  | Deng et al. 2010 |
| Nile tilapia | 2.00 |  |  | El-Banna et al. 1992 |
| Nile tilapia | -0.30 |  |  | El-Enbaawy et al. 1994 |
| Nile tilapia | 5.00 |  |  | El-Enbaawy et al. 1994 |
| sea bass | 2.26 | 1.26 |  | El-Sayed and Khalil 2009 |
| Nile tilapia | 2.30 |  |  | Hegazi et al. 2013 |
| gibel carp |  | 3.00 |  | Huang et al. 2011 |
| Nile tilapia |  | 3.60 |  | Hussain et al. 2017. |
| channel catfish | 4.06 |  |  | Jantrarotai et al. 1990 |
| Nile tilapia | 2.18 |  |  | Magouz 2016. |
| Nile tilapia | 2.30 |  |  | Mafouz 2015 |
| mosquitofish | 2.67 | 2 |  | McKean et al. 2006 |
| mosquitofish | 3 |  |  | McKean et al. 2006 |
| rainbow trout | 3.1 |  |  | Nomura et al. 2011 |
| rainbow trout |  | 3.0 |  | Saei et al.2017 |
| rohu | 3.10 |  |  | Sahoo and Mukherjee 2002 |
| Nile tilapia | 2.30 |  |  | Selim et al. 2014 |
| beluga |  | 2.00 |  | Sepahdari et al. 2010 |
| Nile tilapia | 5.00 |  |  | Tuan et al. 2002 |
| yellow catfish | 3.07 |  |  | Wang, et al. 2016 |
| zebrafish | 2.60 | 2.30 |  | Zhou et al. 2017 |
| zebrafish | 2.30 | 2.00 |  | Zhou et al. 2017 |
| zebrafish | 2.00 | 1.70 |  | Zhou et al. 2017 |
| Nile tilapia |  | 3.48 |  | Zychowski et al. 2013a |
| red drum | 2.00 | 1.48 |  | Zychowski et al. 2013b |
| common carp | 2 |  | oxidative stress | Akter et al. 2010 |
| Nile tilapia | 3.78 |  |  | El-Barbary 2016 |
| common carp | 1.97 |  |  | Kövesi et al. 2018 |
| Nile tilapia | 2.30 |  |  | Mafouz 2015 |

**Table S12** Literature data for zearalenone (ZEN) toxicity in fish as log 10 values of the reported no effect levels (NOEL) and lowest effect levels (LOEL) reported in the studies listed in detail in Annex V, including Atlantic salmon (*Salmo salar*), common carp (*Cyprinus carpio*), fathead minnow (*Pimephalis promelas*), rainbow trout (*Oncorhynchus mykiss*), and zebrafish (*Danio rerio*).

| **ZEN toxicity** | | | | |
| --- | --- | --- | --- | --- |
| **Species** | **log LOEL** | **log NOEL** | **endpoint** | References |
| common carp | 2.52 |  | blood | Pietsch and Junge 2016 |
| rainbow trout | 4 |  |  | Wozny et al. 2012 |
| rainbow trout |  | 4 |  | Wozny et al. 2012 |
| rainbow trout | 3.07 |  |  | Wozny et al. 2015 |
| zebrafish | 3.60 | 1.70 | body composition | Zhou et al. 2017 |
| zebrafish | -1.00 | -3.00 | endocrine | Bakos et al. 2013 |
| fathead minnow | -2.70 |  |  | Johns et al. 2009 |
| common carp | 2.52 |  |  | Pietsch and Junge 2016 |
| zebrafish | 1.70 | 1.00 |  | Chen et al. 2010 |
| zebrafish | 0.00 | -0.49 |  | Schwartz et al. 2010 |
| zebrafish | -1.00 |  |  | Schwartz et al. 2010 |
| zebrafish | -0.55 | -1.13 |  | Schwartz et al. 2011 |
| rainbow trout | 3.07 |  |  | Wozny et al. 2015 |
| Atlantic salmon | 4 | 3.00 |  | Arukwe et al. 1999 |
| Atlantic salmon | 3 | -3.00 |  | Arukwe et al. 1999 |
| zebrafish | -0.02 | -0.55 | growth | Schwartz et al. 2011 |
| fathead minnow | -2.70 |  |  | Johns et al 2009 |
| fathead minnow | -1.30 |  |  | Johns et al. 2009 |
| zebrafish |  | 0.51 | histology | Schwartz et al. 2010 |
| zebrafish | 3.30 | 3.00 |  | Zhou et al. 2017 |
| zebrafish | 3.00 | 2.88 | mortality | Bakos et al. 2013 |
| zebrafish | 3.18 | 3.10 |  | Bakos et al. 2013 |
| zebrafish | 3.10 | 3 |  | Bakos et al. 2013 |
| zebrafish | 2.70 | 2.40 |  | Bakos et al. 2013 |
| zebrafish |  | 0.51 |  | Schwartz et al. 2010 |
| zebrafish | 3.48 | 3.00 |  | Zhou et al. 2017 |
| zebrafish | 3.70 | 3.18 |  | Zhou et al. 2017 |
| zebrafish | 3.18 | 3.00 | development | Bakos et al. 2013 |
| zebrafish | 3.00 | 2.88 |  | Bakos et al. 2013 |
| zebrafish | 2.00 | 1.70 |  | Bakos et al. 2013 |
| fathead minnow | -1.30 | 3.00 |  | Johns et al. 2009 |
| zebrafish | 2.88 | 2.70 | pigmentation | Bakos et al. 2013 |
| zebrafish | 1.40 | 1.70 |  | Bakos et al. 2013 |
| zebrafish | 2.70 | 2.40 | skeleton | Bakos et al. 2013 |
| zebrafish | 2.88 | 2.70 |  | Bakos et al. 2013 |
| common carp | 2.52 |  | immune responses | Pietsch and Junge 2016 |
| common carp | 2.79 |  |  | Pietsch et al. 2015b |
| common carp | 2.90 |  |  | Pietsch et al. 2015c |
| common carp | 2.79 |  | body oxygen demand | Pietsch and Junge 2016 |

**Table S13** Literature data for deoxynivalenol (DON) toxicity in fish as log 10 values of the reported no effect levels (NOEL) and lowest effect levels (LOEL) reported in the studies listed in detail in Annex V, including Atlantic salmon (*Salmo salar*), channel catfish (*Ictalurus punctatus*), common carp (*Cyprinus carpio*), rainbow trout (*Oncorhynchus mykiss*), and zebrafish (*Danio rerio*).

| **DON toxicity** | | | | |
| --- | --- | --- | --- | --- |
| **species** | **log LOEL** | **log NOEL** | **endpoint** | References |
| rainbow trout |  | 3.30 | behaviour | Sisperova et al. 2015 |
| common carp | 2.55 |  | biotransformation | Pietsch et al. 2015d |
| zebrafish | 3.30 | 3.18 |  | Sanden et al. 2012 |
| common carp | 2.98 | 2.81 | blood | Pietsch et al. 2015d |
| common carp | 2.55 |  |  | Pietsch et al. 2014a |
| rainbow trout | 3.29 | 2.35 |  | Matejova et al. 2014 |
| rainbow trout | 4.06 | 3.67 | body composition | Goncalves et al. 2018b |
| rainbow trout | 3.67 | 1.57 |  | Goncalves et al. 2018b |
| Atlantic salmon | 3.57 |  | growth | Döll et al. 2010 |
| rainbow trout | 3.00 |  |  | Woodward et al. 1983 |
| rainbow trout |  | 3.29 |  | Matejova et al. 2014 |
| channel catfish | 3.94 | 3.52 |  | Manning et al. 2014 |
| rainbow trout | 3.52 |  |  | Ryerse et al. 2015 |
| rainbow trout | 3.67 | 1.57 |  | Goncalves et al. 2018b |
| Atlantic salmon | 3.78 |  |  | Bernhoft et al. 2017 |
| zebrafish |  | 3.48 |  | Jorgensen 2012 |
| rainbow trout | 3.29 | 2.35 | histology | Matejova et al. 2014 |
| zebrafish | 4.00 | 3.70 |  | Zhou et al. 2017 |
| common carp | 2.98 | 2.81 |  | Pietsch et al. 2015d |
| common carp | 2.55 |  |  | Pietsch et al. 2014b |
| common carp | 2.55 |  | immune responses | Pietsch et al. 2014a |
| common carp | 2.98 | 2.81 |  | Pietsch et al. 2015a |
| rainbow trout | 3.29 | 2.35 |  | Matejova et al. 2014 |
| rainbow trout | 3.58 |  |  | Ryerse et al. 2015 |
| rainbow trout |  | 4.06 | mortality | Goncalves et al. 2018b |
| rainbow trout | 3.81 |  |  | Ryerse et al. 2015 |
| rainbow trout | 3.61 |  |  | Ryerse et al. 2015 |
| rainbow trout |  | 3.30 |  | Sisperova et al. 2015. |
| zebrafish |  | 4.60 |  | Zhou et al. 2017 |
| channel catfish | 3.74 | 3.52 |  | Manning et al. 2014 |
| rainbow trout | 3.30 |  | oxidative stress | Sisperova et al. 2015 |
| common carp | 2.55 |  |  | Pietsch et al. 2014a |
| rainbow trout | 3.30 |  |  | Sisperova et al. 2015 |
| zebrafish | 3.30 |  |  | Sanden et al. 2012 |
| rainbow trout | 3.30 | 3.30 |  | Sisperova et al. 2015 |

**Table S14** Literature data for ochratoxin A (OTA) toxicity in fish as log 10 values of the reported no effect levels (NOEL) and lowest effect levels (LOEL) reported in the studies listed in detail in Annex V, including channel catfish (*Ictalurus punctatus*), rainbow trout (*Oncorhynchus mykiss*), sea bass (*Dicentrarchus labrax*), and zebrafish (*Danio rerio*).

| **OTA toxicity** | | | | |
| --- | --- | --- | --- | --- |
| **species** | **log LOEL** | **log NOEL** | **endpoint** | References |
| sea bass | 1.70 |  | behaviour | El-Sayed et al. 2009 |
| zebrafish | 1.61 | 1.31 | development | Haq et al. 2016 |
| zebrafish | 2.00 | 1.70 |  | Haq et al. 2016 |
| zebrafish | 2.31 | 1.61 |  | Haq et al. 2016 |
| zebrafish | 2.61 | 2.31 |  | Haq et al. 2016 |
| zebrafish | 1.61 |  | hatching | Haq et al. 2016 |
| zebrafish | 2.49 | 2.20 |  | Tschirren et al. 2018 |
| zebrafish | 2.80 | 2.49 |  | Tschirren et al. 2018 |
| zebrafish | 2.80 | 2.49 | heart rate | Tschirren et al. 2018 |
| rainbow trout | 3.74 |  | mortality | Doster et al. 1972 |
| rainbow trout | 3.67 |  |  | Doster et al. 1972 |
| zebrafish | 2.80 | 2.50 |  | Haq et al. 2016 |
| sea bass |  | 2.60 |  | El-Sayed et al. 2009 |
| sea bass | 2.60 | 2.54 |  | El-Sayed et al. 2009 |
| sea bass | 2.54 | 2.48 |  | El-Sayed et al. 2009 |
| sea bass | 2.48 | 2.40 |  | El-Sayed et al. 2009 |
| channel catfish | 3.90 |  |  | Manning et al. 2003 |
| channel catfish | 3.60 |  |  | Manning et al. 2005 |
| sea bass | 2.46 |  |  | El-Sayed and Khalil 2009 |
| rainbow trout | 3.90 |  | histology | Doster et al. 1972 |
| rainbow trout | 3.60 |  |  | Doster et al. 1972 |
| sea bass | 2.46 |  |  | El-Sayed and Khalil 2009 |
| channel catfish | 3.30 |  |  | Manning et al. 2003 |
| channel catfish | 3.30 | 3.90 | immune responses | Zahran et al. 2016 |
| channel catfish | 3.30 |  | growth | Zahran et al. 2016 |
| channel catfish | 3.90 |  |  | Zahran et al. 2016 |
| channel catfish | 3.00 |  |  | Manning et al. 2003 |
| channel catfish | 3.60 |  |  | Manning et al. 2003 |
| channel catfish | 3.30 |  |  | Manning et al. 2003 |
| zebrafish | 2.19 | 1.89 | oxidative stress | Tschirren et al. 2018 |

**Table S15** Literature data for fumonisin B_1_ (FB_1_) toxicity in fish as log 10 values of the reported no effect levels (NOEL) and lowest effect levels (LOEL) reported in the studies listed in detail in Annex V, including African catfish (*Clarias gariepinus*), Atlantic salmon (*Salmo salar*), channel catfish (*Ictalurus punctatus*), vundu (*Heterobranchus longifilis*), mosquitofish (*Gambusia affinis*), Nile tilapia (*Oreochromis niloticus*), rainbow trout (*Oncorhynchus mykiss*), sea bass (*Dicentrarchus labrax*), and zebrafish (*Danio rerio*).

| **FB1 toxicity** | | | | |
| --- | --- | --- | --- | --- |
| **species** | **log LOEL** | **log NOEL** | **endpoint** | References |
| vundu | 4.63 | 3.37 | blood | Adeyemo et al. 2017 |
| vundu | 4.63 | 3.37 |  | Adeyemo et al. 2017 |
| Nile tilapia | 5.18 |  |  | Tuan et a. 2003 |
| African catfish | 3.70 |  |  | Gbore et al. 2010 |
| common carp | 2.70 |  |  | Pepeljnjak et al. 2003 |
| Atlantic salmon |  | 4.30 | body composition | Carrera García 2013 |
| channel catfish | 4.60 |  |  | Li et al. 1994 |
| channel catfish | 4.00 |  |  | Goel et al. 1994 |
| Atlantic salmon |  | 4.30 | growth | Carrera García 2013 |
| Atlantic salmon | 4.30 |  |  | Carrera García 2013 |
| Nile tilapia | 4.30 |  |  | Claudino-Silva et-al. 2018 |
| rainbow trout |  | 4.30 |  | Piriionen 2016 |
| Nile tilapia | 4.60 |  |  | Tuan et al. 2003 |
| channel catfish | 4.90 |  |  | Lumlertdacha and Lovell 1995 |
| African catfish | 3.70 |  |  | Gbore et al. 2010 |
| African catfish | 4.00 |  |  | Gbore et al. 2010 |
| common carp | 2.70 |  |  | Pepeljnjak et al. 2003 |
| channel catfish | 4.90 |  |  | Yildirim et al. 2000 |
| channel catfish | 4.30 | 4.30 |  | Yildirim et al. 2000 |
| channel catfish | 4.30 |  | histology | Lumbertdacha et al. 1995 |
| common carp | 5.00 |  |  | Kovacić et al. 2009 |
| channel catfish | 4.00 |  |  | Scaff and Scussel 2008 |
| channel catfish common carp | 4.90  4.90 | 4.30 | immune responses | Lumlertdacha and Lovell 1995  Pepeljnjak et al. 2003 |
| Nile tilapia | 2.70 | 2.30 | mortality | Abu-Hassan et al. 2016 |
| mosquitofish | 3.67 | 3.00 |  | McKean et al. 2006 |
| mosquitofish | 4.00 | 3.00 |  | McKean et al. 2006 |
| channel catfish | 5.38 |  |  | Li et al. 1994 |
| common carp | 4.00 | 2.30 |  | Petrinec et al. 2004 |

**Table S16** Literature data for moniliformin (MON) toxicity in fish as log 10 values of the reported no effect levels (NOEL) and lowest effect levels (LOEL) reported in the studies listed in detail in Annex V, including channel catfish (*Ictalurus punctatus*), Nile tilapia (*Oreochromis niloticus*), and zebrafish (*Danio rerio*).

| **MON toxicity** | | | | |
| --- | --- | --- | --- | --- |
| **species** | **log LOEL** | **log NOEL** | **endpoint** | References |
| zebrafish | 2.65 |  | blood | Goncalves et al. 2018c |
| Nile tilapia |  |  |  | Tuan et al. 2003 |
| Nile tilapia |  |  |  | Tuan et al. 2003 |
| channel catfish |  |  |  | Yildirim et al. 2000 |
| Nile tilapia | 4.85 |  | growth | Tuan et al. 2003 |
| channel catfish | 4.30 |  |  | Yildirim et al. 2000 |
| channel catfish | 4.78 |  | histology | Yildirim et al. 2000 |
| zebrafish | 2.95 |  | mortality | Goncalves et al. 2018c |

**Table S17** Literature data for T-2 toxin (T2) toxicity in fish as log 10 values of the reported no effect levels (NOEL) and lowest effect levels (LOEL) reported in the studies listed in detail in Annex V, including channel catfish (*Ictalurus punctatus*), common carp (*Cyprinus carpio*), rainbow trout (*Oncorhynchus mykiss*), and zebrafish (*Danio rerio*).

| **T-2 toxicity** | | | | |
| --- | --- | --- | --- | --- |
| **species** | **log LOEL** | **log NOEL** | **endpoint** | References |
|  |  |  |  |  |
| zebrafish | 1.97 | 1.67 | behaviour | Yuan et al. 2014 |
| common carp | 2.66 |  | biotransformation | Kravchenko et al. 1989 |
| common carp | 3.73 |  | blood | Matejova et al. 2017 |
| rainbow trout | 3.00 |  |  | Modra et al. 2018 |
| rainbow trout | 3.26 | 3.00 |  | Modra et al. 2018 |
| channel catfish | 3.10 | 2.80 |  | Manning et al. 2003 |
| zebrafish | 1.97 | 1.67 | development | Yuan et al. 2014 |
| rainbow trout | 3.70 | 3.40 | growth | Poston et al. 1982 |
| rainbow trout | 2.30 |  |  | Marasas et al 1969 |
| channel catfish | 2.80 |  |  | Manning et al. 2003 |
| channel catfish | 3.70 | 3.40 |  | Manning et al. 2003 |
| rainbow trout | 4.18 |  | histology | Poston et al. 1982 |
| common carp | 3.73 |  | immune responses | Matejova et al. 2017 |
| zebrafish | 2.07 | 1.67 | mortality | Yuan et al. 2014 |
| common carp | 3.61 |  |  | Pelye et al. 2016b |
| channel catfish | 3.40 |  |  | Manning et al. 2003 |
| zebrafish | 1.67 | 1.37 | oxidative stress | Yuan et al. 2014 |
| common carp | 3.39 | 3.09 |  | Balogh et al. |
| common carp | 4.10 | 3.76 |  | Pelye et al. 2016a |
| common carp | 3.61 |  |  | Pelye et al. 2016 b |
| common carp | 3.73 |  |  | Matejova et al. 2017 |
| rainbow trout | 3.00 |  |  | Modra et al. 2018 |
| rainbow trout | 3.26 | 3.00 |  | Modra et al. 2018 |

**Table S18** SR95:5 values for each fish species and the mycotoxins AFB1, ZEN, DON, OTA, FB1, MON and T-2 toxin.

|  | ***AFB1*** | ***ZEN*** | ***DON*** | ***OTA*** | ***FB1*** | ***MON*** | ***T-2*** |
| --- | --- | --- | --- | --- | --- | --- | --- |
| **African catfish** |  |  |  |  | 1.50 |  |  |
| **Atlantic salmon** |  | 2.85 | 1.35 |  |  |  |  |
| **beluga** | 2.20 |  |  |  |  |  |  |
| **gibel carp** | 1.32 |  |  |  |  |  |  |
| **catla** | 1.19 |  |  |  |  |  |  |
| **channel catfish** | 1.41 |  | 1.34 | 1.09 | 1.08 | 1.71 |  |
| **common carp** | 1.24 | 1.19 | 2.17 |  | 1.40 |  |  |
| **fathead minnow** |  | 3.35 |  |  |  |  |  |
| **hybrid tilapia** | 1.04 |  |  |  |  |  |  |
| **rohu** | 1.23 |  |  |  |  |  |  |
| **medaka** | 1.54 |  |  |  |  |  |  |
| **mosquitofish** | 1.68 |  |  |  | 1.50 |  |  |
| **Mossambique tilapia** | 1.67 |  |  |  |  |  |  |
| **Nile tilapia** | 1.17 |  |  |  |  | 1.33 |  |
| **rainbow trout** | 1.19 | 4.51 | 1.07 | 1.05 |  |  |  |
| **red drum** | 1.34 |  |  |  |  |  |  |
| **sea bass** | 4.63 |  |  | 1.29 |  |  |  |
| **silver catfish** | 1.39 |  |  |  |  |  |  |
| **Tra catfish** | 1.51 |  |  |  |  |  |  |
| **zebrafish** | 1.29 | 1.50 | 2.24 | 1.19 | 1.42 | 1.68 |  |


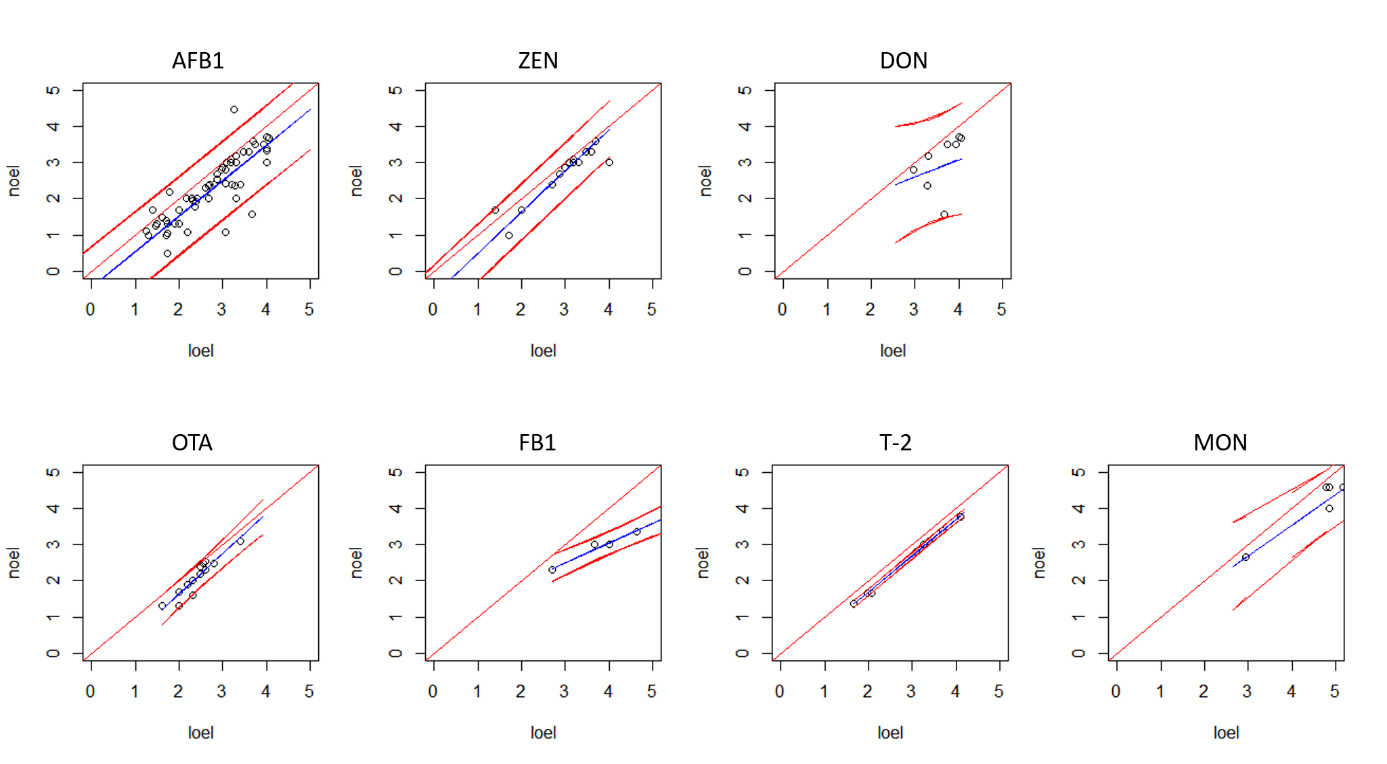


**Figure S11**. Logarithmic LOEL-NOEL plots of the individual mycotoxins used to predict the missing NOEL data by linear modelling according to the R script shown in Annex VI, the red diagonal line depicts the 1:1 correlation between the LOEL and NOEL values, whereas the blue line shows the actual relationship between these values including the intervals for each prediction in red colour.

**Table S19** Summary data for the LOEL to NOEL linear modelling for a number of studies (n) reporting both values at the same time each of the mycotoxins as shown in Figure S11.

|  | **AFB1** | **ZEN** | **DON** | **OTA** | **FB1** | **MON** | **T2** |
| --- | --- | --- | --- | --- | --- | --- | --- |
| minimum | -0.395 | -3.724 | 2.401 | 1.308 | 2.350 | 2.371 | 1.992 |
| 1st Quantile | 1.252 | -0.777 | 2.401 | 2.757 | 3.009 | 3.954 | 2.648 |
| median | 1.804 | 2.240 | 2.753 | 3.092 | 3.212 | 4.471 | 3.207 |
| mean | 1.863 | 1.122 | 2.673 | 2.984 | 3.186 | 4.110 | 3.012 |
| 3rd Quantile. | 2.422 | 2.644 | 2.879 | 3.502 | 3.535 | 4.737 | 3.431 |
| maximum | 3.970 | 3.930 | 2.989 | 3.763 | 3.792 | 4.737 | 3.882 |
| n | 47 | 30 | 16 | 20 | 9 | 8 | 12 |

**Table S20** Summary data for the logarithmic CC5 values for a number of data points (n) derived from the prediction of log NOEL by linear modelling as shown in Table S19.

|  | **AFB1** | **ZEN** | **DON** | **OTA** | **FB1** | **MON** | **T2** |
| --- | --- | --- | --- | --- | --- | --- | --- |
| minimum | 0.228 | -3.630 | 1.377 | 0.491 | 0.794 | -0.953 | -0.328 |
| 1st Quantile | 0.581 | -1.902 | 1.977 | 1.205 | 2.521 | 2.134 | 1.171 |
| median | 0.637 | -1.614 | 2.07 | 1.335 | 2.749 | 2.406 | 1.371 |
| mean | 0.635 | -1.639 | 2.06 | 1.324 | 2.704 | 2.348 | 1.340 |
| 3rd Quantile. | 0.693 | -1.340 | 2.154 | 1.459 | 2.940 | 2.624 | 1.542 |
| maximum | 0.937 | -0.276 | 2.435 | 1.892 | 3.587 | 3.301 | 2.154 |
| n | 247 | 51 | 39 | 38 | 14 | 14 | 14 |
