# Supplementary material for: Risk assessment for mycotoxin contamination in fish feeds in Europe
Source: Mycotoxin Res. 2019 Jul 26;36(1):41–62. doi: 10.1007/s12550-019-00368-6 (PMC6971146; doi:10.1007/s12550-019-00368-6)
Supplement: Supplementary file 5 — (DOCX 48 kb) [file 12550_2019_368_MOESM5_ESM.docx]

Annex V: References for Toxicity of Mycotoxins in Fish

Abd-Allah GA, El-Fayoumi RI, Smith MJ, Heckmann RA, O'Neill KL (1999) Comparative evaluation of aflatoxin B-1 genotoxicity in fish models using the Comet assay. Mutat Res/Genet Toxicol Environ Mutagen 446(2):181–188.

Abu-Hassan FAM, Khalil RH, Saad TT, Amer MT, Abdel-Latif HMR (2016) Histopathological outcomes designating the toxicological aspects of fumonisin B1 on cultured Nile tilapia, *Oreochromis niloticus.* Int J Fish Aquat Stud 4(3):52–60.

Adeyemo BT, Tiamiyu LO, Ayuba VO (2017) Serum biochemistry and lipids profiling in experimental dietary exposure of *Heterobranchus longifilis* catfish juveniles to graded concentrations of fumonisin B1. Int J Aquacult 7(5): 31–41.

Akter A, Rahman M, Hasan M (2010) Effects of aflatoxin B1 on growth and bioaccumulation in common carp fingerling in Bangladesh. Asia-Pacif J Rural Dev 20(2):1–13.

Al-Faragi JK (2014) The efficacy of prebiotic (β-Glucan) as a feed additive against toxicity of aflatoxin B1 in common carp, *Cyprinus carpio* L. J Aquac Res Development 5:4.

Andleeb S, Ashraf M, Hafeez-ur-Rehman M, Jabbar MA, Abbas F, Younus M (2015) Effect of aflatoxin B1-contaminated feed on growth and vital ogans of advanced fry of *Catla catla*. J Anim Plant Sci 25(3):816–824.

Arana S, Tabata YA, Sabino M, Rigolino MG, Hernandez-Blazquez FJ (2002) Differential effect of chronic aflatoxin B1 intoxication on the growth performance and incidence of hepatic lesions in triploid and diploid rainbow trout (*Oncorhynchus mykiss*). Arch Med Vet 34(2) Valdivia, http://dx.doi.org/10.4067/S0301-732X2002000200011.

Arukwe A, Grotmol T, Haugen TB, Knudsen FR, Goksøyr A (1999) Fish model for assessing the in vivo estrogenic potency of the mycotoxin zearalenone and its metabolites. Sci Tot Environ 236:153-161.

Ayyat MS, Ayyat AMN, Al-Sagheera AA, El-Aziz A, El-Haisc M (2018) Effect of some safe feed additives on growth performance, blood biochemistry, and bioaccumulation of aflatoxin residues of Nile tilapia fed aflatoxin-B1 contaminated diet. Aquacult 495:27–34.

Ayyat MS, Abd Rhman GA, El-Marakby HI, Hessein Amira AA (2014) Aflatoxin B1 toxicity and its reduction by using coumarin and vitamin E in Nile tilapia. Zagazig J Agricult Res 41(1).

Ayyat MS, Abd Rhman GA, El-Marakby HI, Mahmoud HK, Hessan AAA (2013) Reduction the aflatoxin toxicity in the Nile tilapia fish. Egypt J Nutr Feeds 16(2):469–479.

Ayres JL, Lee DJ, Wales JH, Sinnhuber RO (1971) Aflatoxin structure and hepatocarcinogenicity in rainbow trout (*Salmo gairdneri*). J Nat Cancer Inst 46:561–564.

Baglodi V, Jayaraj EG, Nesara KM, Abhiman PB (2015) Effect of dietaryincorporated aflatoxin (AFB1) on the survival and growth performance of *Labeo rohita*. J Exp Zool India 18(2):603–607.

Bailey GS, Goeger DE, Hendricks JD (1989) Factors influencing experimental carcinogenesis in laboratory fish models. In: Metabolism of Polynuclear Hydrocarbons in the Aquatic Environment (Varanasi U, ed). Boca Raton, FL:CRC Press 253–268.

Bailey GS, Price RL, Park DL, Hendricks JD (1994) Effect of ammoniation of aflatoxin B1-contaminated cottonseed feedstock on the aflatoxin M1 content of cows' milk and hepatocarcinogenicity in the trout bioassay. Food Chem Toxicol 32(8):707–715.

Bailey GS, Dashwood R, Loveland PM, Pereira C, Hendricks JD (1998) Molecular dosimetry in fish: quantitative target organ DNA adduction and hepatocarcinogenicity for four aflatoxins by two exposure routes in rainbow trout. Mutat Res 339:233–244.

Bailey GS, Williams DE, Hendricks JD (1996) Fish models for environmental carcinogenesis: The rainbow trout. Environ Health Perspect 104(Suppl 1): 5–21.

Bakos K, Kovacs R, Stasny A, Sipos DK, Urbanyi B, Müller F, Csenki Z, Kovacs B (2013) Developmental toxicity and estrogenic potency of zearalenone in zebrafish (*Danio rerio*). Aquat Toxicol 136-137:13–21.

Balogh K, Heincinger M, Fodor J, Mezes M (2009) Effect of long term feeding of T-2 and HT-2 toxin contaminated diet on the glutathione redox status and lipid peroxidation processes in common carp (*Cyprinus carpio* L.). Acta Biol Szeged 53:23–27.

Bastardo H, Sofia S, Nava L, Rangel C (2006) Effects of aflatoxin B1 concentration and exposure time on hepatic condition in the rainbow trout. Interciencia 31(6):437–440.

Bauer DH, Lee DJ, Sinnhuber RO (1969) Acute toxicity of aflatoxin B1 and G1 in the rainbow trout (*Salmo gairdneri*). Toxicol Appl Pharmacol 15(2):415–419.

Bedoya-Sernal CM, Michelin EC, Massocco MM, Carrion LCS, Godoy SHS, Lima CG, Ceccarelli PS, Yasui GS, Rottinghaus GE, Sousa RLM, Fernandes AM (2018) Effects of dietary aflatoxin B1 on accumulation and performance in matrinxã fish (*Brycon cephalus*). PLoS ONE 13(8):e0201812.

Bernhoft A, Høgåsen HR, Rosenlund G, Ivanova L, Berntssen MHG, Alexander J, Eriksen GS, Kruse Fæste C (2017) Tissue distribution and elimination of deoxynivalenol and ochratoxin A in dietary-exposed Atlantic salmon (*Salmo salar*). Food Addit Contam A 34(7):1211–1224.

Black JJ, Maccubbin AE, Schiffert M (1985) A reliable, efficient, microinjection apparatus and methodology for the in vivo exposure of rainbow trout and salmon embryos to chemical carcinogens. J Nat Cancer Inst 75(6):1123–1128.

Breinholt V, Hendricks J, Pereira C, Arbogast D, Bailey G (1995) Dietary chlorophyllin is a potent inhibitor of aflatoxin B1 hepatocarcinogenesis in rainbow trout. Cancer Res 55:57–62.

Carlson DB, Williams DE, Spitsbergen JM, Ross PF, Bacon CW, Meredith FI, Riley RT (2001) Fumonisin B-1 promotes aflatoxin B-1 and N-methyl-N'-nitro-nitrosoguanidine-initiated liver tumors in rainbow trout. Toxicol Appl Pharmacol 172(1):29–36.

Carrera García E (2013) Effects of fumonisin B1 on performance of juvenile Baltic salmon (*Salmo salar*). Master’s thesis. University of Jyväskylä, Faculty of Science, Department of Biological and Environmental Science, International Aquatic Masters Programme, Finnland.

Cagauan AG, Tayaban RH, Somga JR, Bartolome RM (2004) Effect of aflatoxin-contaminated feeds in Nile tilapia (*Oreochromis niloticus* L.). In: Bolivar R, Mair G, Fitzsimmons K, editors. Proceedings of the 6^th^ international symposium on tilapia in aquaculture. Philippines: Manila; 2004, 12-16.

Carpenter HM, Zhang Q, Elzahr C, Selivonchick DP, Brock DE, Curtis LR (1995) In-vitro and in-vivo temperature modulation of hepatic metabolism and DNA adduction of aflatoxin-B1 in rainbow trout. J Biochem Toxicol 10(1):1–10.

Chavez-Sanches MC, Martinez CA, Moreno IO (1994) Pathological effects of feeding young *Oreochromis niloticus* diets supplemented with different levels of aflatoxin B1. Aquacult 127:49–60.

Chen H, Hu J, Yang J, Wang Y, Xu H, Jiang Q, Gong Y, Gu Y, Song H (2010) Generation of a fluorescent transgenic zebrafish for detection of environmental estrogens. Aquat Toxicol 96:53–61.

Claudino-Silva SC, Lala B, Mora NHAP, Schamber CR, Nascimento CS, Pereira VV, Hedler DL, Gasparino E (2018) Challenge with fumonisins B1 and B2 changes IGF-1 and GHR mRNA expression in liver of Nile tilapia fingerlings. World Mycotox J 11(2):237–245.

Curtis LR, Zhang Q, El-Zahr C, Carpenter HM, Miranda CL, Buhler DR, Selivonchick DP, Arboast DN, Hendricks JD (1995) Temperature-modulated incidence of aflatoxin B1-initiated liver cancer in rainbow trout. Fundam Appl Toxicol 25(1):146–153.

Dashwood RH, Fong AT, Arbogast DN, Bjeldanes LF, Hendricks JD, Bailey GS (1994) Anticarcinogenic activity of indole-3-carbinol acid products: ultrasensitive bioassay by trout embryo microinjection. Cancer Res 54:3617–3619.

Deng SX, Tian LX, Liu FJ, Jin SJ, Liang GY, Yang HJ, Du ZY, Liu YJ (2010) Toxic effects and residue of aflatoxin B. Aquacult. 307:233–240.

Döll S, Valenta H, Baardsen G, Möller P, Koppe W, Stubhaug I, Dänicke S (2011) Effects of increasing concentrations of deoxynivalenol, zearalenone and ochratoxin A in diets for Atlantic salmon (*Salmo salar*) on performance, health and toxin residues. In Proceedings of 33^rd^ Mycotoxin Workshop, Freising, Germany, 30 May–1 June 2011.

Doster RC, Sinnhuber RO, Pawlowski NE (1974) Acute intraperitoneal toxicity of ochratoxin A and B derivatives in rainbow trout (*Salmo gairdneri*). Food Cosmet Toxicol 12:499–505.

El-Banna R, Teleb HM, Hadi MM, Fakhry FM (1992) Performance and tissue residues of tilapias fed dietary aflatoxin. Vet Med J 40:17–23.

El-Barbary MI (2016) Detoxification and antioxidant effects of garlic and curcumin in *Oreochromis niloticus* injected with aflatoxin B1 with reference to gene expression of glutathione peroxidase (GPx) by RT-PCR. Fish Physiol Biochem 42:617–629.

El-Barbary MI (2018) Impact of garlic and curcumin on the hepatic histology and cytochrome P450 gene expression of aflatoxicosis *Oreochromis niloticus* using RT-PCR. Turk J Fish Aquat Sci 18:405–415.

El-Boshy ME, El-Ashram AMM, Abd El-Ghany NA (2008) Effect of dietaty beta1,3 glucan on immunomodulation od diseased *Oreochromins niloticus* experimentally infected with aflatoxin B1. 8^th^ International Symposium on Tilapia in Aquaculture 2008, 1109–1127.

El-Enbaawy M, Adel M, Marzouk MS, Salem AA (1994) The effect of acute and chronic aflatoxicosis on the immune functions of *Oreochromis niloticus* in Egypt. Vet Med J Giza 42:47–52.

El-Sayed YS, Khalil RH (2009) Toxicity, biochemical effects and residue of aflatoxin B1 in marine water-reared sea bass (*Dicentrarchus labrax* L.). Food Chem Toxicol 47:1606–1609.

El-Sayed YS, Khalil RH, Saad TT (2009) Acute toxicity of ochratoxin-A in marine water-reared sea bass (*Dicentrarchus labrax* L.). Chemosphere 75:878–882.

Farabi SMV, Yousefian M, Hajimoradloo A (2007) Aflatoxicosis in juvenile *Huso huso* fed a contaminated diet. J Appl Ichthyol 22:234–237.

Gbore FA, Adewole AM, Oginni O, Oguntolu MF, Bada AM, Akele O (2010) Growth performance, haematology and serum biochemistry of African catfish (*Clarias gariepinus*) fingerlings fed graded levels of dietary fumonisin B1. Mycotox Res 26:221–227.

Goel S, Lenz SD, Lumlertdacha S, Lovell RT, Shelby RA, Li M, Riley RT, Kemppainen BW (1994) Sphingolipid levels in catfish consuming *Fusarium moniliforme* corn culture material containing fumonisins. Aquat Toxicol 30:285–294.

Gonçalves RA, Cam TD, Tri NN, Santos G, Encarnação P, Hung LT (2018a) Aflatoxin B1 (AFB1) reduces growth performance, physiological response, and disease resistance in Tra catfish (*Pangasius hypophthalmus*). Aquacult Int 26:921–936.

Gonçalves RA, Navarro-Guillén C, Gilannejad N, Dias J, Schatzmayr D, Bichl G, Czabany T, Moyano FJ, Rema P, Yúfera M, Mackenzie S, Martínez-Rodríguez G (2018b) Impact of deoxynivalenol on rainbow trout: Growth performance, digestibility, key gene expression regulation and metabolism. Aquacult 490:362–372.

Gonçalves RA, Tarasco M, Schatzmayr D, Gavaia P (2018c) Preliminary evaluation of moniliformin as a potential threat for teleosts. Fishes 3:4.

Hagelberg S, Hult K, Fuchs R (1989) Toxicokinetics of ochratoxin A in several species and its plasma-binding properties. J Appl Toxicol 9(2):91–96.

Halver JE (1969) Aflatoxicosis and trout hepatoma. In: Goldblatt LA (Ed.) Aflatoxin: Scientific Background, Control, and Implications. Academic Press, NewYork, pp. 265–306.

Han D, Xie S, Zhu X, Yang Y, Guo Z (2009) Growth and hepatopancreas in gibel carp fed diets containing low levels of aflatoxin B1. Aquacult Nutr 16(4):335–342.

Haq M, Gonzalez N, Mintz K, Jaja-Chimedza A, De Jesus CL, Lydon C, Welch AZ, Berry JP (2016) Teratogenicity of ochratoxin A and the degradation product, ochratoxin, in the zebrafish (*Danio rerio*) embryo model of vertebrate development. Toxins 8:40.

Hatanaka J, Doke N, Harada T, Aikawa T, Enomoto M (1982) Usefulness and rapidity of screening for the toxicity and carcinogenicity of chemicals in medaka *Oryzias latipes*. Jpn J Exp Med 52:243–253.

Hegazi SM, El-Sabagh MR, El-Keeidy A, Zein El-Dein AI (2013) Aflatoxin in feed and its effect on fish health. Kafreilsheik Vet Med J 11(2):317–329.

Hendricks JD, Wales JH, Sinnhuber RO, Nixon JE, Loveland PM, Scanlan RA (1980) Rainbow trout (*Salmo gairdneri*) embryos: a sensitive animal model for Proc Am Soc Exp Biol 39:3222–3229.

Huang Y, Han D, Zhu X, Yang Y, Jin J, Chen Y, Xie S (2011) Response and recovery of gibel carp from subchronic oral administration of aflatoxin B1. Aquacult 319:89–97.

Hussain D, Mateen A (2017) Alleviation of aflatoxin-B1 toxicity by using clay adsorbent in Nile tilapia (*Oreochromis niloticus*) diets. Pakistan J Zool 49(2):425–431.

Hussain M, Gabal MA, Wilson T, Summerfelt RC (1993) Effect of aflatoxin contaminated feed on morbidity and residues in walleye fish. Vet Human Toxicol 35(5):396–398.

Hussein SY, Mekkawy IA, Moktar ZZ, Mubarak M (2000) Protective effect of *Nigella sativa* seed against aflatoxicosis in *Oreochromis niloticus*. Mycotoxin Conf Mycotoxins Environ Poland Bydgoszez 25–27:109–130.

Imani A, Bani MS, Noori F, Farzaneh M, Mohanlou KS (2017) The effect of bentonite and yeast cell wall along with cinnamon oil on aflatoxicosis in rainbow trout (*Oncorhynchus mykiss*): Digestive enzymes, growth indices, nutritional performance and proximate body composition. Aquacult 476:160–167.

Jantrarotai W, Lovell RT (1990) Subchronic toxicity of dietary aflatoxin B1 to channel catfish. J Aquat Anim Health 2:248–254.

Jantrarotai W, Lovell RT, Grizzle JM (1990) Acute toxicity of aflatoxin B1 to channel catfish. J Aquat Anim Health 2:237–247.

Johns SM, Denslow ND, Kane MD, Watanabe KH, Orlando EF, Sepulveda MS (2009) Effects of estrogens and antiestrogens on gene expression of fathead minnow (*Pimephales promelas*) early life stages. Environ Toxicol 26:195–206.

Jorgensen S (2012) Evaluating primary (cry1Ab) and secondary effects (deoxynivalenol) of GM maize when fed to zebrafish (*Danio rerio*): Investigating growth, intestinal mRNA and white blood cell differentiation. Master Thesis, Department of Biology, University of Bergen, Norway.

Kovačić S, Pepeljnjak S, Petrinec Z, Šegvić Klarić M (2009) Fumonisin B1 neurotoxicity in young carp (*Cyprinus carpio* L.). Arh Hig Rada Toksikol 60:419–426, doi 2478/10004-1254-60-2009-1974.

Kövesi B, Pelyhe C, Zándoki E, Mézes M, Balogh K (2018) Changes of lipid peroxidation and glutathione redox system, and expression of glutathione peroxidase regulatory genes as effect of short-term aflatoxin B1 exposure in common carp. Toxicon 144:103–108.

Kravchenko LV, Galash VT, Avreneva LT, Kranauskas AE (1989) On the sensitivity of carp, *Cyprinus carpio*, to mycotoxin T-2. J Ichthyol 29:156–160.

Li MH, Raverty SA, Robinson EH 1994. Effects of dietary mycotoxins produced by the mold *Fusarium moniliforme* on channel catfish (*Ictalurus punctatus*). J. World Aquacult Soc 25(4):512–516.

Lim HA, Ng WK, Lim SL, Ibrahim CO (2001) Contamination of palm kernel meal with *Aspergillus flavus* affects its nutritive value in pelleted feed for tilapia, *Oreochromis mossambicus.* Aquacult Res 32(11):895–905.

Lovell RT (1992) Mycotoxins: hazardous to farmed fish. Feed Int 13:24–28.

Lumlertdacha S, Lovell RT, Shelby RA, Lenz SD, Kemppainen BW (1995) Growth, hematology, and histopathology of channel catfish (*Ictalurus punctatus*), fed toxins from *Fusarium moniliforme.* Aquacult 130:201–218.

Lumlertdacha S, Lovell RT (1995) Fumonisin-contaminated dietary corn reduced survival and antibody production by channel catfish challenged with *Edwardsiella ictaluri*. J Aquat Anim Health 7(1): 1–8.

Madhusudhanan N, KavithaLakshmi SN, Shanmugasundaram KR, Shanmugasundaram ERB (2004) Oxidative damage to lipids and proteins induced by aflatoxin B-1 in fish (*Labeo rohita*)-protective role of Amrita Bindu. Environ Toxicol Pharmacol 17(2):73–77.

Madhusudhanan N, Kavithalakshmi SN, Shanmugasundaram ER, Shanmugasundaram KR (2006) Aflatoxin B1-induced DNA damage in *Labeo rohita*: protective effect of an antioxidant supplement, Amrita Bindu. Basic Clin Pharmacol Toxicol 98:473–479.

Mafouz ME, Sherif AH (2015) A multiparameter investigation into adverse effects of aflatoxin on *Oreochromis niloticus* health status. J Basic Appl Zool 71:48–59.

Mafouz ME (2015) Ameliorative effect of curcumin on aflatoxin B1-induced changes in liver gene expression of *Oreochromis niloticus*. Molec Biol 49(2):275–286.

Magouz FI, Eweedah NM, Salem MFE, Amer AA (2016) Detoxification of aflatoxin contaminated ration by chemical, biological and spices methods in Nile tilapia (*Oreochromis niloticus*) diets. J. Agric. Res. Kafr El-Sheikh Univ 42(4):102–119.

Manning BB, Li MH, Robinson EH, Gaunt PS, Camus AC, Rottinghaus GE (2003) Response of channel catfish to diets containing T-2 toxin. J Aquat Anim Health 15:229–238.

Manning BB, Terhune JS, Li MH, Robinson EH, Wise DJ, Rottinghaus GE (2005) Exposure to feedborne mycotoxins T-2 toxin or ochratoxin A causes increased mortality of channel catfish challenged with *Edwardsiella ictaluri.* J Aquat Anim Health 17(2):147–152.

Manning BB, Abbas HK, Wise DJ, Greenway T (2014) The effect of feeding diets containing deoxynivalenol contaminated corn on channel catfish (*Ictalurus punctatus*) challenged with *Edwardsiella ictaluri*. Aquacult Res 45:1782–1786.

Marasas WFO, Bamburg JR, Smalley EB, Strong FM, Ragland WL, Degurse BE (1969). Toxic effect on trout, rats, and mice of T-2 toxin produced by the fungus *Fusarium tricinctum* (Cd.) Snyd. et Hans. Toxicol Appl Pharmacol 15:471–482.

Marijani E, Nasimolo J, Kigadye E, Gnonlonfin GJB, Okoth S (2017) Sex-related differences in hematological parameters and organosomatic indices of *Oreochromis niloticus* exposed to aflatoxin B1 diet. Scientifica Article ID 4268926, 7 pages, https://doi.org/10.1155/2017/4268926.

Matejova I, Modra H, Blahova J, Franc A, Fictum P, Sevcikova M, Svobodova Z (2014) The effect of mycotoxin deoxynivalenol on haematological and biochemical indicators and histopathological changes in rainbow trout (*Oncorhynchus mykiss*). BioMed Res Int, Article ID 310680, 5 pages, http://dx.doi.org/10.1155/2014/310680.

Matejova I, Vicenova M, Vojtek L, Kudlackova H, Nedbalcova K, Faldyna M, Sisperova E, Modra H, Svobodova Z (2015) Effect of the mycotoxin deoxynivalenol on the immune responses of rainbow trout (*Oncorhynchus mykiss*). Veterinarni Medicina 60(9):515–521.

Matejova I, Faldyna M, Modra H, Blahova J, Palikova M, Markova Z, Franc A, Vicenova M, Vojtek L, Bartonkova J, Sehonova P, Hostovsky M, Svobodova Z (2017) Effect of T-2 toxin-contaminated diet on common carp (*Cyprinus carpio* L.). Fish Shellfish Immunol 60:458–465.

McKean C, Tang L, Tang M, Billam M, Wang Z, Theodorakis CW, Kendall RJ, Wang J-S (2006) Comparative acute and combinative toxicity of aflatoxin B1 and fumonisin B1 in animals and human cells. Food Chem Toxicol 44:868–876.

Modra H, Sisperova E, Blahova J, Enevova V, Fictum P, Franc A, Mares J, Svobodova Z (2018) Elevated concentrations of T-2 toxin cause oxidative stress in the rainbow trout (*Oncorhynchus mykiss*). Aquacult Nutr 24:842–849.

Mohapatra S, Sahu NP, Pal AK, Prusty AK, Kumar V, Kumar S (2011) Haemato-immunology and histo-architectural changes in *Labeo rohita* fingerlings: effect of dietary aflatoxin and mould inhibitor. Fish Physiol Biochem 37:177–186.

Nguyen AT, Grizzle JM, Lovell RT, Manning BB, Rottinghaus EG (2002) Growth and hepatic lesions of Nile tilapia *Oreochromis niloticus* fed diets containing aflatoxin B1. Aquacult 212:311–319.

Nixon JE, Hendricks JD, Pawlowski NE, Pereira CB, Sinnhuber RO, Bailey GS (1984) Inhibition of aflatoxin B1 carcinogenesis in rainbow trout by flavone and indole compounds. Carcinogen 5(5):615–619.

Nomura H, Ogiso M, Yamashita M, Takaku H, Kimura A, Chikasou M, Nakamura Y, Fujii S, Watai M, Yamada H (2011) Uptake by dietary exposure and elimination of aflatoxins in muscle and liver of rainbow trout (*Oncorhynchus mykiss*). J Agric Food Chem 59(9):5150–5158.

Nunez O, Hendricks JD, Duimstra JR (1991) Ultrastructure of hepatocellular neoplasms in aflatoxin B1 (AFB1)-initiated rainbow trout (*Oncorhynchus mykiss*). Toxicol Pathol 19:11–23.

Oganesian A, Hendricks JD, Pereira CB, Orner GA, Bailey GS, Williams DE (1999) Potency of dietary indole-3-carbinol as a promoter of aflatoxin B1-initiated hepatocarcinogenesis: results from a 9000 animal tumor study. Carcinogen 20(3):453–458.

Orner GA, Hendricks JD, Williams DE (1993) Enhancement of aflatoxin B1-initiated hepatocarcinogenesis in trout by dietary administration of peroxides. Proc Amer Assoc Cancer Res 34:184.

Ottinger CA, Kaattari SL (2000) Long-term immune dysfunction in rainbow trout (*Oncorhynchus mykiss*) exposed as embryos to aflatoxin B_1_. Fish Shellfish Immunol 10:101–106.

Pelyhe C, Kövesi B, Zándoki E, Kovács B, Szabó-Fodor J, Mézes M, Balogh K (2016a) Short-term effects of T-2 toxin or deoxynivalenol on lipid peroxidation and the glutathione system in common carp. Acta Vet Hungar 64(4):449–466.

Pelyhe C, Kövesi B, Zándoki E, Kovács B, Szabó-Fodor J, Mézes M, Balogh K (2016b) Effect of 4-week feeding of deoxynivalenol or T-2-toxin-contaminated diet on lipid peroxidation and glutathione redox system in the hepatopancreas of common carp (*Cyprinus carpio* L.). Mycotoxin Res 32:77–83.

Pepeljnjak S, Petrinec Z, Kovacic S, Segvic M (2002) Screening toxicity study in young carp (*Cyprinus carpio*) on feed amended with fumonisin B1. Mycopathol 156:139–145.

Petrinec Z, Pepeljnjak S, Kovacic S, Krznaric A (2004) Fumonisin B causes multiple lesions in common carp (*Cyprinus carpio*), Dtsch Tierarztl Wschr 111:341–380.

Pietsch C, Kersten S, Valenta H, Dänicke S, Schulz C, Kloas W, Burkhardt-Holm P (2014a) In vivo effects of deoxynivalenol (DON) on innate immune responses of carp (*Cyprinus carpio* L.). Food Chem Toxicol 68:44–52.

Pietsch C, Schulz C, Rovira P, Kloas W, Burkhardt-Holm P (2014b) Organ damage and altered nutritional condition in carp (*Cyprinus carpio* L.) after food-borne exposure to the mycotoxin deoxynivalenol (DON). Toxins 6:756–778.

Pietsch C, Katzenback BA, Garcia Garcia E, Schulz C, Belosevic M, Burkhardt-Holm P (2015a) Acute and subchronic effects on immune responses of carp after exposure to deoxynivalenol (DON) in feed. Mycotoxin Res 31(3):151–164.

Pietsch C, Kersten S, Valenta H, Dänicke S, Burkhardt-Holm P, Junge R (2015b) Effects of dietary exposure to zearalenone (ZEN) on carp (*Cyprinus carpio* L.). Toxins 7:3465–3480.

Pietsch C, Junge R, Burkhardt-Holm P (2015c) Immunomodulation by zearalenone (ZEN) in carp (*Cyprinus carpio* L.). BioMed Res Int.Article ID 420702, 9 pages, doi:10.1155/2015/420702.

Pietsch C, Burkhardt-Holm P (2015d). Feed-borne exposure to deoxynivalenol (DON) leads to acute and chronic effects on liver enzymes and histology in carp (*Cyprinus carpio* L.). World Mycotoxin J 8(5):619–627.

Pietsch C, Junge R (2016) Physiological responses of carp (*Cyprinus carpio* L.) to dietary exposure to zearalenone (ZEN). Comp Biochem Physiol C 188:52–59.

Pietsch C (2017) Zearalenone (ZEN) and its influence on regulation of gene expression in carp (*Cyprinus carpio* L.) liver tissue. Toxins 9: 283.

Piiroinen LM (2016) The effects of dietary fumonisin B1 on growth and physiology of rainbow trout (*Oncorhynchus mykiss*). Master’s thesis. University of Jyväskylä, Faculty of Science, Department of Biological and Environmental Science, International Aquatic Masters Programme, Finnland.

Poston HA, Coffin JL, Combs GF (1982) Biological effects of dietary T-2 toxin on rainbow trout, *Salmo gairdneri*. Aquatic Toxicol 2(2):79–88.

Rahman ANA, Abdellatief SA, Mahboub HHH (2017) Protection of Nile tilapia, *Oreochromis niloticus* from aflatoxin B1 toxicity by dietary supplementation with Fennel essential oil and *Saccharomyces cerevisiae*. Egypt J Aquat Res 43:235–240.

Ryerse IA, Hooft JM, Bureau DP, Hayes MA, Lumsden JS (2015) Purified deoxynivalenol or feed restriction reduces mortality in rainbow trout, *Oncorhynchus mykiss* (Walbaum), with experimental bacterial coldwater disease but biologically relevant concentrations of deoxynivalenol do not impair the growth of *Flavobacterium psychrophilum*. J Fish Dis 38:809–819.

Saei MM, Taee HM, Siahpoust S, Taheri M (2017) Effects of toxin binder Biotox on growth performance survival, enzymatic activity, hematologichal and biochemical parameters of fingerlings rainbow trout (*Oncorhynchus mykiss*) fed diets-contaminated with aflatoxin. J Aquac Res Develop S2, doi 10.4172/2155-9546.S2-013.

Sahoo PK, Mukherjee SC (2001) Immunosuppressive effects of aflatoxin B1 in Indian major carp (*Labeo rohita*). Comp Immunol Microbiol Infect Dis 24: 143–149.

Sahoo PK, Mukherjee SC (2002a) Effect of dietary beta-1,3 glucan on immune responses and disease resistance of healthy and aflatoxin B-1-induced immunocompromised rohu (*Labeo rohita* Hamilton)

Sahoo PK, Mukherjee SC (2002b) The effect of dietary immunomodulation upon. *Edwardsiella tarda* vaccination in healthy and immunocompromised Indian major carp (*Labeo rohita*). Fish Shellfish Immunol 12:1–16.

Sahoo PK, Mukherjee SC (2003) Immunomodulation by dietary vitamin C in healthy and aflatoxin B1-induced immunocompromised rohu (*Labeo rohita*). Comp Immunol Microbiol Infect Dis 26: 65–76.

Sahoo PK, Mukherjee SC, Jain AK, Mukherjee A (2003) Histopathological and electron microscopic studies of gills and opisthonephros of rohu, *Labeo rohita* to acute and subchronic aflatoxin B1 toxicity. Asian Fish Sci 16:257–268.

Sanden M, Jorgensen S, Hemre G-I, Ornrud R, Sissener NH (2012) Zebrafish (*Danio rerio*) as a model for investigating dietary toxic effects of deoxynivalenol contamination in aquaculture feeds. Food Chem Toxicol 50:4441–4448.

Sato S, Matsushima T, Tanaka N, Sugimura T, Takashima F (1973) Hepatic tumors in the guppy (*Lebistes reticulatus*) induced by aflatoxin BI, dimethylnitrosamine, and 2-acetylaminofluorene. J Natl Cancer Inst 50:765–778.

Scaff RMC, Scussel VM (2008) Ultra-structural and histochemical analysis of channel catfish (*Ictalurus punctatus*) liver treated with fumonisin B1. Brazil Arch Biol Technol 51(2):333–344.

Schwartz P, Thorpe KL, Bucheli TD, Wettstein FE, Burkhardt-Holm P (2010) Short-term exposure to the environmentally relevant estrogenic mycotoxin zearalenone impairs reproduction in fish. Sci Total Environ 409:326–333.

Schoenhard GL, Hendricks JD, Nixon JE, Lee DJ, Wales JH, Sinnhuber RO, Pawlowski NE (1981) Aflatoxicol-induced hepatocellular carcinoma in rainbow trout (*Salmo gairdneri*) and the synergistic effects of cyclopropenoid fatty acids. Cancer Res (3):1011–1014.

Selim KM, El-hofy H, Khalil RH (2014) The efficacy of three mycotoxin adsorbents to alleviate aflatoxin B1-induced toxicity in *Oreochromis niloticus.* Aquacult Int 22:523–540.

Sepahdari A, Ebrahimzadeh Mosavi HA, Sharifpour I, Khosravi A, Motallebi AA, Mohseni M, Kakoolaki S, Pourali HR, Hallajian A (2010) Effects of different dietary levels of AFB1 on survival rate and growth factors of Beluga (*Huso huso*). Iran J Fish Sci 9(1):141–150.

Shahafve S, Banaee M, Haghi BN, Mohiseni M (2017) Histopathological study of common carp (*Cyprinus carpio*) fed aflatoxin-contaminated diets. Int J Aquat Biol 5(2):63–70.

Shelton DW, Hendricks JD, Coulombe RA, Bailey GS (1984) Effect of dose on the inhibition of carcinogenesis/mutagenesis by Aroclor 1254 in rainbow trout fed aflatoxin B1. J Toxicol Environ Health 13:649–657.

Sherif AH, Abdel-Maksoud SA, Shukry MM (2013) Study on toxicity of *Oreochromis niloticus* with aflatoxin B1. Egypt J Aquat Biol Fish 17(3):107–119.

Sinnhuber RO, Bailey GS (1984) Inhibition of aflatoxin B1 carcinogenesis in rainbow trout by flavone and indole compounds. Carcinogen 5:615–619.

Sinnhuber RO, Wales JH, Ayres JL, Engebrecht RH, Amend DL (1968) Dietary factors and hepatoma in rainbow trout (*Salmo gairdneri*). 1. Aflatoxins in vegetable protein feedstuffs. J Natl Cancer Inst 41:711–718.

Sisperova E, Modra H, Zikova A, Kloas W, Blahova J, Matejova I, Zivna D, Svobodova Z (2015) The effect of mycotoxin deoxynivalenol (DON) on the oxidative stress markers in rainbow trout (*Oncorhynchus mykiss*, Walbaum 1792). J Appl Ichthyol 31:855–861.

Soares Lopes PR, Fernandes Pouey JLO, Schoffen Enke DB, Mallmann CA, Kich HA, Bromberger Soquetta M (2009) Utilização de adsorvente em rações contendo aflatoxina para alevinos de jundiá. R Bras Zootec 38(4):589–595.

Spring P, Fegan DF (2010) Mycotoxins—A Rising Threat to Aquaculture; Alltech Inc.: Brentwood, TN, USA.

Srour TM (2004) Effect of ochratoxin-A with or without Biogen® on growth performance, feed utilization and carcass composition of Nile tilapia (*Oreochromis niloticus*) fingerlings. J Agric Sci Mansoura Univ Egypt 29:51–61.

Svobodova Z, Piskac A (1980) Effect of feeds with a low content of aflatoxin B1 on the health of carp *Cyprinus carpio*. Zivocisna Vyroba–UVTIZ 25(11):809–814.

Svobodova Z, Piskac A, Havlikova J, Groch L (1982) Influence of feed with different contents of B1 aflatoxin on the carp health condition. Zivocisna Vyroba–UVTIZ 27(11):811–820.

Thorgaard GH, Arbogast DN, Hendricks JD, Pereira CB, Bailey GS (1999) Tumor suppression in triploid trout. Aquat Toxicol 46:121–126.

Tilton SC, Gerwick LG, Hendricks JD, Rosato CS, Corley-Smith G, Givan SA, Bailey GS, Bayne CJ, Williams DE (2005) Use of a rainbow trout oligonucleotide microarray to determine transcriptional patterns in aflatoxin B1-induced hepatocellular carcinoma compared to adjacent liver. Toxicol Sci 88(2):319–330.

Troxel CM, Reddy AP, ONeal PE, Hendricks JD, Bailey GS (1997) In vivo aflatoxin B-1 metabolism and hepatic DNA adduction in zebrafish (*Danio rerio*). Toxicol Appl Pharmacol 143:213–220.

Tschirren L, Siebenmann S, Pietsch C (2018) Toxicity of ochratoxin to early life stages of zebrafish (*Danio rerio*). Toxins 10:264.

Tuan NA, Grizzle JM, Lovell RT, Manning BB, Rottinghaus GE (2002) Growth and hepatic lesions of Nile tilapia (*Oreochromis niloticus*) fed diets containing aflatoxin B1. Aquacult. 212:311–319.

Tuan NA, Manning BB, Lovell RT, Rottinghaus GE (2003) Responses of Nile tilapia (*Oreochromis niloticus*) fed diets containing different concentrations of moniliformin of fumonisin B1. Aquacult 217:515–528.

Varior S, Philip B (2012) Aflatoxin B1 induced alterations in the stability of the lysosomal membrane in *Oreochromis mossambicus* (Peters, 1852). Aquacult Res 43:1170–1175.

Wales JH, Sinnhuber RO, Hendricks JD, Nixon JE, Eisele TA (1978) Aflatoxin B1 induction of hepatocellular carcinoma in the embryos of rainbow trout (*Salmo gairdneri*). J Natl Cancer Inst 60(5):1133–1139.

Wang X, Wang Y, Li Y, Huang M, Gao Y, Xue X, Zhang H, Encarnação P, Santos G, Gonçalves RA (2016) Response of yellow catfish (*Pelteobagrus fulvidraco*) to different dietary concentrations of aflatoxin B1 and evaluation of an aflatoxin binder in offsetting its negative effects. Ciencias Marinas 42(1):15–29.

Weigt S, Huebler N, Strecker R, Braunbeck T, Broschard TH (2011) Zebrafish (*Danio rerio*) embryos as a model for testing proteratogens. Toxicol 281:25–36.

Woodward B, Young LG, Lun AK (1983) Vomitoxin in diets for rainbow trout (*Salmo gairdneri*). Aquacult 35:93–101.

Wozny M, Brzuzan P, Gusiatin M, Jakimiuk E, Dobosz S, Kuźmiński H (2012) Influence of zearalenone on selected biochemical parameters in juvenile rainbow trout (*Oncorhynchus mykiss*). Polish J Vet Sci 15(2):221–225.

Woźny M, Dobosz S, Obremski K, Hliwa P, Gomułka P, Łakomiak A, Różyński R, Zalewski T, Brzuzan P (2015) Feed-borne exposure to zearalenone leads to advanced ovarian development and limited histopathological changes in the liver of premarket size rainbow trout, Aquacult 448:71–81.

Yildirim M, Manning BB, Lovell RT, Grizzle JM, Rottinghaus GE. (2000) Toxicity of moniliformin and fumonisin B1 fed singly and in combination in diets for young channel catfish *Ictalurus punctatus*. J World Aquac Soc 31:599–608.

Yuan G, Wang Y, Yuan X, Zhang T, Zhao J, Huang L, Peng S (2014) T-2 toxin induces developmental toxicity and apoptosis in zebrafish embryos. J Environ Sci 26:917–925.

Zahran E, Manning B, Seo J-K, Noga EJ (2016) The effect of Ochratoxin A on antimicrobial polypeptide expression and resistance to water mold infection in channel catfish (*Ictalurus punctatus*). Fish Shellfish Immunol 57:60–67.

Zhang Q, Suorsasuper K, Curtis LR (1992) Temperature-modulated aflatoxin B1 hepatic disposition and formation and persistence of DNA adducts in rainbow trout. Toxicol Appl Pharmacol 113(2):253–259.

Zhou H, George S, Li C, Gurusamy S, Sun X, Gong Z, Qian H (2017) Combined toxicity of prevalent mycotoxins studied in fish cell line and zebrafish larvae revealed that type of interactions is dose-dependent. Aquat. Toxicol. 193:60–71.

Zychowski KE, Pohlenz C, Mays T, Romoser A, Hume M, Buentello A, Gatlin III DM, Phillips TD (2013a) The effect of NovaSil dietary supplementation on the growth and health performance of Nile tilapia (*Oreochromis niloticus*) fed aflatoxin-B1 contaminated feed. Aquacult 376-379:117–123.

Zychowski KE, Hoffmann AR, Ly HJ, Pohlenz C, Buentello A, Romoser A, Gatlin DM, Phillips TD (2013b) The effect of aflatoxin-B1 on red drum (*Sciaenops ocellatus*) and assessment of dietary supplementation of NovaSil for the prevention of aflatoxicosis. Toxins 5:1555–1573.
