# Supplementary material for: Risk assessment for mycotoxin contamination in fish feeds in Europe
Source: Mycotoxin Res. 2019 Jul 26;36(1):41–62. doi: 10.1007/s12550-019-00368-6 (PMC6971146; doi:10.1007/s12550-019-00368-6)
Supplement: Supplementary file 6 — (DOCX 18 kb) [file 12550_2019_368_MOESM6_ESM.docx]

Annex VI: R script for data prediction

data.file <- read.csv("file.csv",header=T,sep=";",row.names=NULL, dec=",", na.strings="NA", as.is=T)

head(data.file)

str(data.file)

data.file$loel <- as.numeric(data.file$loel)

data.file$noel <- as.numeric(data.file$noel)

**# Create vectors with unique level IDs for the toxins**

data.file$toxin <- "AFLA" to select individual toxins from the csv table

toxin.out <- unique(data.file$toxin)

**# Fit linear model and complement missing noel values**

model1 <- lm(noel ~ loel, data = data.file)

summary(model1)

model1.res <- resid(model1)

plot(model1.res)

**# Source function to calculate CC5 values by resampling**

source("File as indicated at the end of this document")

estimate.cc5 <- function(dat, M=10000) by a loop containing

dat.samp <- sample(dat,length(dat),replace=TRUE)

output = matrix(ncol=5, nrow=M)

for (j in 1:M) {

resamp.j = LHCp.AJ(dat.samp, gamma=c(.5, .975, .025))

CC5.j = rLHCp.AJ(1, dat.samp)

output[j,] = c(CC5.j, resamp.j, length(dat))

output = data.frame(output)

names(output) = c("one.CC5", "CC5.median", "CC5.upper", "CC5.lower", "n")

output

LHCp.AJ = function(data, p = 5, gamma = 0.50) {n = length(data)kappa = qt(gamma,n - 1,sqrt(n) * qnorm(1 - p/100)) / sqrt(n)

mean(data) - sd(data) * kappa

**# Infer CC-quantiles**

dat.select <- data.file[data.file$toxin == toxin.out[i],"noel.pred"]

dat.out <- estimate.cc5(dat.select,M=10000)

dat.collect[[toxin.out[i]]] <- quantile(dat.out$one.CC5, probs=c(.5, .025, .975))

**# Display the box plots**

boxplot(dat.out$one.CC5)

boxplot(data.file$noel.pred)

points(1,dat.out$one.CC5)

points(rep(1:nrow(dat.out)),dat.out$one.CC5)

points(rep(1,nrow(dat.out)),dat.out$one.CC5)

points(rep(1,nrow(dat.out)),dat.out$one.CC5, col ="red")

quantile(data.file$noel.pred,probs = 0.05)

**# Display the kernel density plots and summary tables**

plot(density(dat.out$one.CC5))

plot(density(dat.out$one.CC5), main = "ZEN", xlab = "CC5 estimate", ylab = "Probability", col = "red")

summary(dat.out)

**# Contents of the Source File**

## Function to calculate CC5 +/- using normal dist model of Aldenberg & Jaworska (2000)

LHCp.AJ = function(data, p = 5, gamma = 0.50) {

n = length(data)

kappa = qt(

gamma,

n - 1,

sqrt(n) * qnorm(1 - p/100)) / sqrt(n)

mean(data) - sd(data) * kappa

}

## Function to sample one or more CC5 values from posterior using

## normal dist model of Aldenberg & Jaworska (2000)

rLHCp.AJ = function(n.sample, data, p=5) {

n = length(data)

zstar = qnorm(1 - p/100)

T = rt(

n.sample,

n - 1,

-sqrt(n) * zstar

)

mean(data) + T * sd(data)/sqrt(n)

}

do.sample = function(data, reg.fit, M=10000) {

output = matrix(ncol=5, nrow=M)

## Calculate variables needed from LOEL-NOEL regression model to

## sample y-values given x-values

betahat = coef(reg.fit)

p.reg = length(betahat)

n.reg = nrow(model.frame(reg.fit))

df.reg = n.reg - p.reg

sse = sum(resid(reg.fit)^2)

XTXinv = summary(reg.fit)$cov.unscaled

log.noel = subset(data, !is.na(log.noel))$log.noel

x.log.loel = subset(data, is.na(log.noel))$log.loel

n.x = length(x.log.loel)

n = nrow(data)

for (j in 1:M){

tau = rgamma(1, df.reg/2, sse/2)

sigma = 1/sqrt(tau)

beta = rmvnorm(1, betahat, sigma^2*XTXinv)

y.log.noel.j = beta[1] + beta[2]*x.log.loel + rnorm(n.x, 0, sigma)

log.noel.j = c(log.noel, y.log.noel.j)

resamp.j = LHCp.AJ(log.noel.j, gamma=c(.5, .975, .025))

CC5.j = rLHCp.AJ(1, log.noel.j)

output[j,] = c(CC5.j, resamp.j, n)

}

output = data.frame(output)

names(output) = c("one.CC5", "CC5.median", "CC5.upper", "CC5.lower", "n")

output

}

CC5.summary = function(logdata) {

result = LHCp.AJ(logdata, gamma=c(.5, .975, .025))

names(result) = c("CC5.median", "CC5.lower", "CC5.upper")

c(result, n=length(logdata))

}

# Function to calculate CV

co.var <- function(x,na.rm=TRUE) 100*(sd(x,na.rm=na.rm)/mean(x,na.rm=na.rm))
